# A new basal bird from China with implications for morphological diversity in early birds

**DOI:** 10.1038/srep19700

**Published:** 2016-01-25

**Authors:** Min Wang, Xiaoli Wang, Yan Wang, Zhonghe Zhou

**Affiliations:** 1 Key Laboratory of Vertebrate Evolution and Human Origins of Chinese Academy of Sciences, Institute of Vertebrate Paleontology and Paleoanthropology, Chinese Academy of Sciences, Beijing 100044, China; 2 State Key Laboratory of Palaeobiology and Stratigraphy, Nanjing Institute of Geology and Palaeontology, Chinese Academy of Sciences, Nanjing, 210008, China; 3 Institute of Geology and Paleontology, Linyi University, Linyi, Shandong 276000, China; 4 Shandong Tianyu Museum of Nature, Pingyi, Shandong 273300, China

## Abstract

The Chinese Lower Cretaceous Jehol Group is the second oldest fossil bird-bearing deposit, only surpassed by *Archaeopteryx* from the German Upper Jurassic Solnhofen Limestones. Here we report a new bird, *Chongmingia zhengi* gen. et sp. nov., from the Jehol Biota. Phylogenetic analyses indicate that *Chongmingia zhengi* is basal to the dominant Mesozoic avian clades Enantiornithes and Ornithuromorpha, and represents a new basal avialan lineage. This new discovery adds to our knowledge regarding the phylogenetic differentiation and morphological diversity in early avian evolution. The furcula of *Chongmingia* is rigid (reducing its efficiency), consequently requiring more power for flight. However, the elongated forelimb and the large deltopectoral crest on the humerus might indicate that the power was available. The unique combination of features present in this species demonstrates that numerous evolutionary experimentations took place in the early evolution of powered flight. The occurrence of gastroliths further confirms that herbivory was common among basal birds. The Jehol birds faced competition with pterosaurs, and occupied sympatric habitats with non-avian theropods, some of which consumed birds. Thus, avialan herbivory may have reduced ecological competition from carnivorous close relatives and other volant vertebrates early in their evolutionary history.

Over the past three decades, numerous feathered non-avian dinosaurs and birds have been collected from the Early Cretaceous Jehol Biota of northeastern China, making it arguably the most important avifauna for the study of the early evolution of birds^1^,^2^. Representatives of all major clades of Mesozoic avian groups have been reported from those deposits, including the long bony tailed Jeholornithiformes, the basal pygostylians Sapeornithiformes and Confuciusornithiformes, Enantiornithes and Ornithuromorpha^3^. Enantiornithes are the most specious Mesozoic avian clade and form the sister group to Ornithuromorpha, the clade that includes all living birds^4^. This wealth of exquisitely preserved avian specimens helps to bridge the considerably large morphological and biological gaps between *Archaeopteryx* and crown birds^5^, showing a more gradual change in some aspects of avian evolution as well as demonstrating some unusual morphologies and homoplasy. A new species, represented by a single new skeleton from the Jiufotang Formation of the Jehol Group, sheds light on the early evolution of birds. Phylogenetic analysis places this new specimen in a position basal to Ornithothoraces—the clade including Enantiornithes and Ornithuromorpha. Although our analysis suggests that the new specimen may represent the most phylogenetically basal Cretaceous bird known to date, this phylogenetic hypothesis should be treated with caution given the incomplete preservation of the skeleton and low phylogenetic support values. Histological studies indicate *Chongmingia* had a moderately elevated growth rate relative to the long-tailed *Archaeopteryx* and *Jeholornis*. Furthermore, other morphological features, along with the evolutionary pattern drawn from other basal birds, reveal mosaic evolution and numerous evolutionary experiments relating to powered flight early in the evolution of birds.

## Results

### Systematic paleontology

Aves Linnaeus, 1758 *Chongmingia zhengi* gen. et sp. nov.

#### Etymology

The generic name is from the Mandarin word *Chongming*, referring to a Chinese mythological bird. The specific epithet is in honour of Mr. Xiaoting Zheng for his generous contribution in the establishment of the Shandong Tianyu Museum of Nature.

#### Holotype

**Figure 1:**
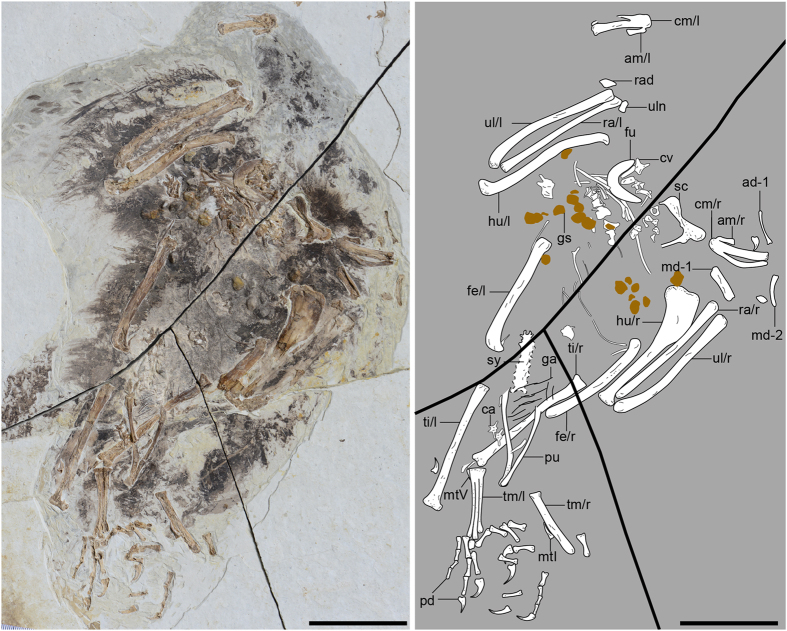
Photograph and line drawing of the holotype of *Chongmingia zhengi* gen. et sp. nov. (STM9-9). Abbreviations: ad-1, proximal phalanx of the alular digit; am, alular metacarpal; ca, caudal vertebra; cm, carpometacarpus; cv, cervical vertebra; fe, femur; fu, furcula; ga, gastralia; gs, gastroliths; hu, humerus; md-1, 2, phalanx 1, 2 of the major digit; mtI, metatarsal I; mtV, metatarsal V; pd, pedal digit; pu, pubis; ra, radius; rad, radiale; sc, scapulocoracoid; sy, synsacrum; ti, tibiotarsus; tm, tarsometatarsus; ul, ulna; uln, ulnare; l/r, left or right side. Scale bars, 50 mm. (The photograph and line drawing were taken by Min Wang).

STM (Shandong Tianyu Museum of Nature) 9-9, a partial skeleton with associated soft tissues and gastroliths, missing the skull and most of the caudal vertebrae (Fig. 1).

#### Locality and horizon

Dapingfang, Liaoning Province, China; Jiufotang Formation, Early Cretaceous (Aptian).

#### Diagnosis

A large non-ornithothoracine bird distinguishable from the known basal avialans by the following combination of features: the furcula is boomerang-shaped and robust, with an interclavicular angle of 68° (compared to 83° in *Archaeopteryx*, 84° in *Confuciusornis*, and 108° in *Sapeornis*); the coracoid and scapula are fused and form a scapulocoracoid; the proximal margin of humerus is concave centrally and bears an expanded deltopectoral crest; the alular metacarpal is long relative to the length of the carpometacarpus, with a length ratio of 0.32 (compared to 0.21 in *Jeholornis* and 0.23 in *Sapeornis*); the minor metacarpal is strongly bowed caudally, creating a wide intermetacarpal space with the major metacarpal; the proximal tarsals are fused to the tibia; the forelimb is short relative to the hindlimb (length ratio is 1.07, compared to 1.30 in *Jeholornis*, and 1.52 in *Sapeornis*); and pedal digit II is longer than IV.

#### Anatomical description and comparisons

Most pre-synsacral vertebrae are missing (Fig. 1). The synsacrum is estimated to comprise 7–8 vertebrae (Supplementary Fig. S1). Two free caudal vertebrae can be recognized. The transverse processes are caudolaterally directed, exceeding the width of the centrum in length (Supplementary Fig. S1). At least five pairs of gastralia are present (Fig. 1; see Supplementary Information for additional description).

**Figure 2:**
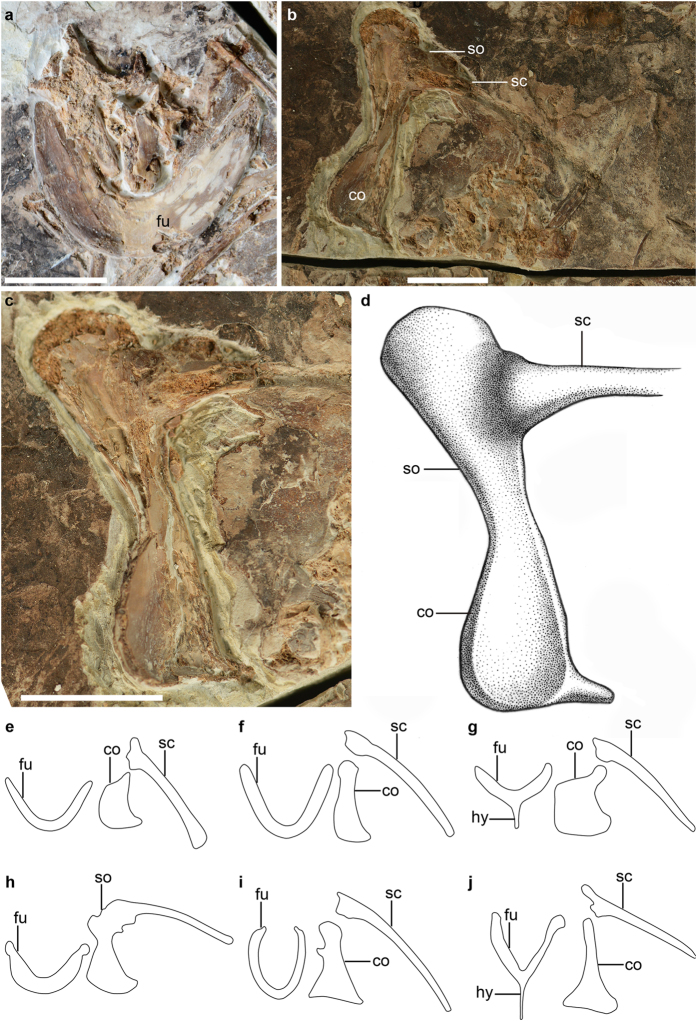
Pectoral girdle of *Chongmingia zhengi* gen. et sp. nov. (STM9-9), in comparison with other basal avialans. (**a**) Furcula of STM9-9 in cranial view; (**b**) the right scapulocoracoid of STM9-9 in medial view; (**c**) photograph and (**d**) line drawing of the right scapulocoracoid of STM9-9 in close-up view; (**e–j**) line drawing of the pectoral girdles of other basal avialans (not scaled): (**e**) *Archaeopteryx* (after ref. 7); (**f**) *Jeholornis prima*; (**g**) *Sapeornis chaoyangensis*; (**h**) *Confuciusornis sanctus*; (**i**) *Archaeorhynchus spathula*; (**j**) *Parabohaiornis martini*. Abbreviations: co, coracoid; fu, furcula; hy, hypocleidium; sc, scapula; so, scapulocoracoid. Scale bars, 10 mm (**a–c**). (The photographs and line drawings were taken by Min Wang).

The furcula is more robust than in many other basal avialans and more derived taxa such as enantiornithines and ornithuromorphs (Fig. 2a). As in *Archaeopteryx*, *Confuciusornis* and the non-avian theropod *Xiaotingia*^6^,^7^,^8^, the furcula is boomerang-shaped without a hypocleidium (Fig. 2a,e–j), but the interclavicular angle is smaller (68°, compared to 83° in *Archaeopteryx*, 84° in *Confuciusornis*, and 75° in *Xiaotingia*, respectively). The furcula is morphologically similar to that of *Jixiangornis orentalis*, a poorly known taxon considered as the junior synonym of *Jeholornis prima*^3^, but not by everyone^9^. The bone is weakly curved craniocaudally. The ramus constricts at its omal end without the caudal projection reported in *Confuciusornis*^4^. The furcular symphysis is broad and excavated cranially by a shallow depression, and the depression is bounded laterally by a pair of knobs located on the ventral margin of the furcula. Both features are absent in *Archaeopteryx*, *Sapeornis* and *Confuciusornis*. As in *Confuciusornis* and some non-avian theropods^6^, the scapula and coracoid are fused into a scapulocoracoid (Fig. 2b–d), a primitive feature absent in *Archaeopteryx*, *Sapeornis*, *Jeholornis*, and more derived birds (Fig. 2e–g,i,j)^7^,^10^,^11^,^12^. The coracoid and scapula are exposed in medial view and form an angle of 90° (Fig. 2c). As in Enantiornithes and more basal avialans such as *Jeholornis prima* and *Sapeornis*, the procoracoid process is absent^10^,^11^,^12^. In contrast, a procoracoid process is developed in *Jeholornis curvipes*^9^. The omal half of the coracoid widens craniocaudally, and rapidly narrows, becoming craniocaudally compressed over its sternal half (Fig. 2c). The lateral margin of the coracoid is concave. The sternolateral corner is pointed as in *Jeholornis*^9^, in contrast to the blunt condition present in *Sapeornis*. The scapula is poorly preserved, and its impression indicates that the blade is straight and shorter than the humerus.

**Figure 3:**
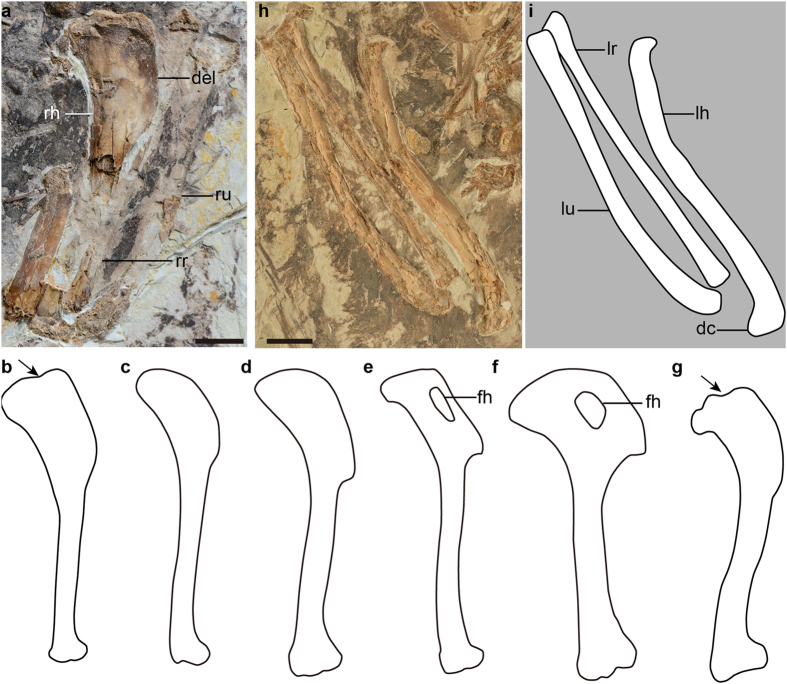
Forelimb of *Chongmingia zhengi* gen. et sp. nov. (STM9-9), in comparison with other basal avialans. (**a**) Right humerus of STM9-9 in caudal view; (**b–g**) line drawing of the humerus of other basal avialans (not scaled): (**b**) STM9-9; (**c**) *Archaeopteryx* (the Bürgermeister-Müller specimen); (**d**) *Jeholornis prima*; (**e**) *Sapeornis chaoyangensis*; (**f**) *Confuciusornis sanctus*; (**g**) *Pterygornis dapingfangensis*; (**h**) photograph and (**i**) line drawing of the left ulna and radius of STM9-9. Abbreviations: dc, dorsal condyle of the humerus; del, deltopectoral crest; fh, fenestra of the humerus; lh, left humerus; lr, left radius; lu, left ulna; rh, right humerus; rr, right radius; ru, right ulna. The arrows in (**b,g**) indicate the concave proximal margin of the humerus. Scale bars, 10 mm (**a,h**). (The photographs and line drawings were taken by Min Wang).

As in *Confuciusornis*, the forelimb is slightly longer than the hindlimb, with an intermembral index (humerus + ulna + major metacarpus/femur + tibia + metatarsal III) of ~1.07, considerably shorter than in *Jeholornis* (1.30 in *Jeholornis prima*, 1.23 in *Jeholornis curvipes*), *Sapeornis* (1.52), and more derived ornithothoracines (Supplementary Table S1 and S2). In contrast to the condition of non-avian theropods such as *Xiaotingia*^8^, the humerus is longer than the femur like that of most Mesozoic birds^13^. The central part of the humeral head is concave and bounded by elevated dorsal and ventral regions (Fig. 3a,b), a feature present in enantiornithines^12^. However, the corresponding margin is convex in *Archaeopteryx*, *Jeholornis*, *Sapeornis* and *Confuciusornis* (Fig. 3c–g). The proximocaudal surface lacks any evidence of a fossa characteristic of more derived birds. The width of the deltopectoral crest approaches that of the mid-shaft of the humerus, and is considerably wider than that of *Archaeopteryx* (Fig. 3b,c). The crest is rectangular in outline with a straight dorsal margin, lacking the fenestra and the concave distal margin characteristic of *Sapeornis* and *Confuciusornis* (Fig. 3e,f)^6^,^11^.

**Figure 4:**
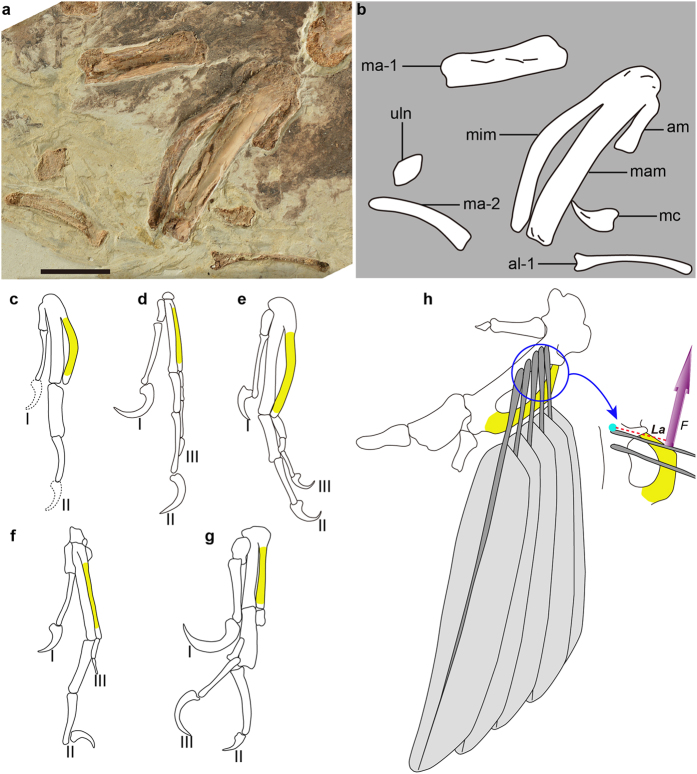
Hands of *Chongmingia zhengi* gen. et sp. nov. (STM9-9), in comparison with other basal avialans. (**a**) Photograph and (**b**) line drawing of the right hand of STM9-9; (**c–g**) line drawings of hands of other basal avialans (not scaled): (**c**) reconstructed hand of STM9-9; (**d**) *Archaeopteryx* (after ref. 7); (**e**) *Jeholornis prima*; (**f**) *Sapeornis chaoyangensis* (after ref. 11); (**g**) *Confuciusornis sanctus*; (**h**) interpretative drawing of modern bird’s hand with primary remiges in dorsal view. The remiges are attached to the major metacarpal and supported by the minor metacarpal; the lift force (upward purple vector, ***F***) provided by the minor metacarpal is increased by the increased the lever arm (red dashed line, ***La***) when the minor metacarpal is caudally bowed. Abbreviations: I-III, alular, major, minor digit; al-1, the proximal phalanx of the alular digit; am, alular metacarpal; ma-1, 2, phalanges 1, 2 of the major digit; mam, major metacarpal; mc, manual claw; mim, minor metacarpal; uln, ulnare. Scale bars, 10 mm (**a**). (The photographs and line drawings were taken by Min Wang).

**Figure 5:**
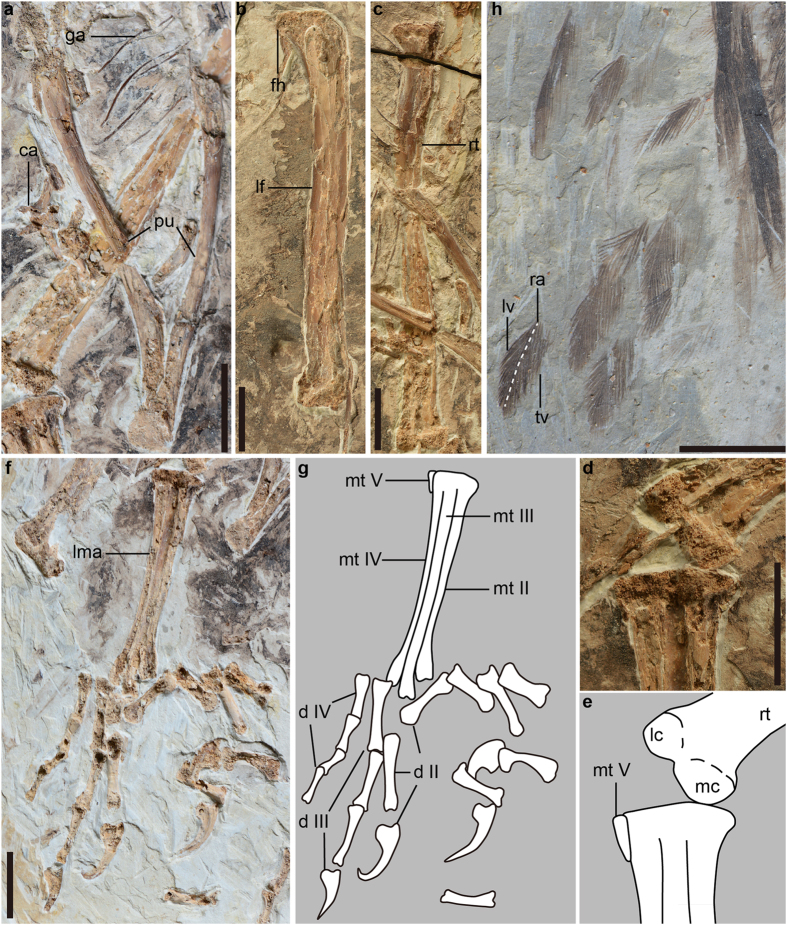
Hindlimb and feathers of *Chongmingia zhengi* gen. et sp. nov. (STM9-9). (**a**) Pubis; (**b**) left femur; (**c**) right tibiotarsus; (**d**) photograph and (**e**) line drawing of the distal right tibiotarsus and the proximal left tarsometatarsus; (**f**) photograph and (**g**) line drawing of the left foot; (**h**) preserved distal portions of the left wing (the white dash line indicating the rachis). Abbreviations: ca, caudal vertebrae; d II–IV, digit II, III and IV; fh, femoral head; ga, gastralia; lc lateral condyle; lf, left femur; lma, left tarsometatarsus; lv, leading vane; mc, medial condyle; mt II–V, metatarsal II, III, IV and V; pu, pubis; ra, rachis; rt, right tibiotarsus; tv, trailing vane. Scale bars, 10 mm (**a–d,f,h**). (The photographs and line drawings were taken by Min Wang).

The ulna is approximately the same length as the humerus (Fig. 3h,i). The radius is straight and robust, and its midshaft width is approximately 83% that of the ulna, proportionally stouter than the state in *Jeholornis* (0.64 in *Jeholornis prima*, 0.54 in *Jeholornis curvipes*), *Sapeornis* (0.42), and *Confuciusornis* (0.61). The major and minor metacarpals, although ankylosed proximally to each other and to the semilunate carpal, are not fused distally (Fig. 4a,b), a condition commonly present among basal avialans except *Jeholornis curvipes* (Fig. 4c–g)^6^,^9^,^10^,^11^. The alular metacarpal is fused only proximally to the major metacarpal, and an extensor process is not present. The alular metacarpal is long relative to the major metacarpal (length ratio of 0.32), compared to *Jeholornis* (0.25 in *Jeholornis prima*, 0.17 in *Jeholornis curvipes*), and *Sapeornis* (0.23; Supplementary Table S2). The minor metacarpal is strongly bowed caudally, forming a wide intermetacarpal space with the straight major metacarpal (Fig. 4a), resembling that of *Jeholornis* (Fig. 4e). By contrast, in other basal avialans including enantiornithines, the minor metacarpal is straight and contacts the major metacarpal tightly, rendering the intermetacarpal space indiscernible (Fig. 4d,f,g)^6^,^7^,^8^,^11^. The minor and major metacarpals are subequal in distal projection, as in *Archaeopteryx*, *Sapeornis*, *Jeholornis* and *Confuciusornis*^6^,^7^,^9^,^10^,^11^. The first phalanx of the alular digit is slender and gently curved (Fig. 4a,b). Although disarticulated, the preserved length indicates that this phalanx reaches the distal end of the major metacarpal as in the basalmost enantiornithine *Protopteryx* (Fig. 4c)^14^, but is shorter than that in *Archaeopteryx* and *Confuciusornis* (Fig. 4d,g). By contrast, that phalanx terminates proximal to the major metacarpal in *Jeholornis* and *Sapeornis* (Fig. 4e,f). As in *Sapeornis* and more derived birds, the penultimate phalanx of the major digit is shorter than the more proximal phalanx (Fig. 4c,f)^11^. That differs from the condition in most non-avian theropods and basal avialans, such as scansoriopterygids, *Archaeopteryx*, *Jeholornis* and *Confuciusornis* (Fig. 4d,e,g)^8^,^15^,^16^. The pubes are retroverted and have an oval cross section. Distally, their shafts converge and form a symphysis (Fig. 5a).

The femur is approximately 88% the length of the tibiotarsus, and is proportionally longer than in *Archaeopteryx* (0.67; Supplementary Table S1 and S2). The greater and lesser trochanters are fused and form a trochanteric crest that extends as far proximally as the femoral head (Fig. 5b). As in *Confuciusornis*, *Sapeornis* and more derived birds^6^,^11^, the calcaneum and astragalus are fused to the tibia in *Chongmingia*, forming a tibiotarsus (Fig. 5c,d). That fusion is absent in non-avian dinosaurs, *Archaeopteryx* and *Jeholornis*^7^,^10^,^17^. Cnemial crests are absent as in most enantiornithines and more basal avialans. The distal condyles have an hourglass form, and taper medially towards each other (Fig. 5d,e). In non-avian theropods, *Chongmingia* and *Confuciusornis*^6^, the medial condyle is mediolaterally wider than the lateral one (Fig. 5e), but they are of equal width in *Sapeornis*^11^. The fibula is more than 70% the length of the tibiotarsus, and it fails to contact the proximal tarsals, as in other non-ornithothoracine birds except *Archaeopteryx*.

The distal tarsals are fused to the proximal ends of metatarsals II–IV, but the three major metatarsals are unfused distally (Fig. 5d–g). As in *Archaeopteryx*, *Jeholornis*, *Confuciusornis* and *Sapeornis*, the fifth metatarsal is present (Fig. 5d,e)^6^,^9^,^10^,^11^; metatarsal V measures ~10% of the length of metatarsal III, considerably shorter than that in *Jeholornis curvipes* (0.37). Metatarsal III is the longest, closely followed by metatarsal IV, and the distal end of metatarsal II is proximal to the trochlea of metatarsal III (Fig. 5f,g). Metatarsals II and IV are of equal width, and both are narrower than metatarsal III. The trochlea of metatarsal II is poorly ginglymoid, and metatarsal IV is reduced to a single articular condyle distally. The right metatarsal I is exposed in dorsolateral view, exhibiting a concave proximolateral surface for articulation with metatarsal II. The distal half of metatarsal I is plantarly deflected, and the articulation of the hallux is nearly perpendicular to the articulation of metatarsal II. The third pedal digit is the longest and approximately the same length as the tarsometatarsus (Fig. 5f). The second digit is slightly longer than the fourth digit as in some enantiornithines^18^, and differs from the state in *Archaeopteryx*, *Confuciusornis* and *Jeholornis*^6^,^7^,^10^. The longest non-ungual phalanx is phalanx II-2 in *Chongmingia*, but the longest is phalanx I-1 in *Sapeornis*^11^, and phalanx III-1 in *Archaeopteryx* and *Jeholornis curvipes*^7^,^9^. Unlike *Archaeopteryx* and *Jeholornis*, the phalanges do not decrease in length distally, suggesting less cursorial adaptation (and perhaps more arboreal) in this species.

Feathers are preserved as carbonized traces and surround the whole skeleton (Figs 1 and 5h). Given their dense and poor preservation, only two major types of feathers can be recognized, the soft coverts and the pennaceous remiges. The coverts are rachis-less, soft, hair-like, and occur all over the body (Fig. 1). On the left wing, at least seven remiges (primaries or secondaries) are recognized based on their preserved distal ends (Fig. 5h). They are highly asymmetrical, with the trialing vane approximately twice as wide as the leading vane. The preserved rachis is narrow and light in color, as in *Archaeopteryx*^19^, but the feathers lack the longitudinal dark medial strip widely present in other Jehol birds^4^,^20^.

#### Histological description

**Figure 6:**
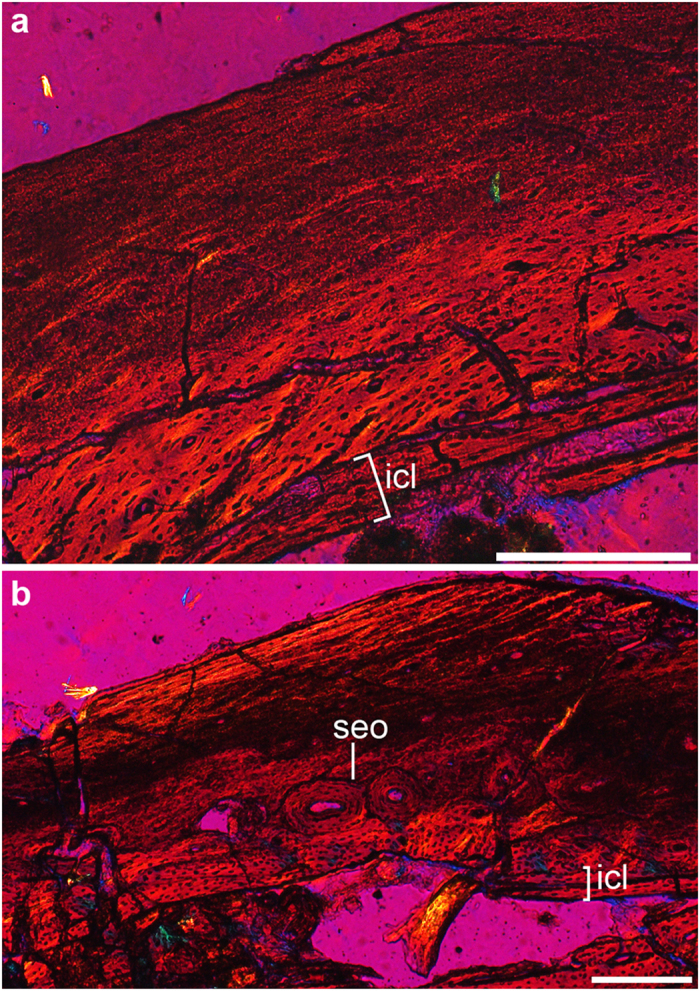
Long bone histology of *Chongmingia zhengi* gen. et sp. nov. (STM9-9). Cross sections of the right humerus (**a**) and right femur (**b**) viewed under polarized light microscopy. Abbreviations: icl, inner circumferential layer; seo, secondary osteon. Scale bars, 100 μm (**a**), 200 μm (**b**). (The photographs were taken by Min Wang).

We prepared histological cross sections of the humerus and femur from STM9-9. The humeral cross section is mainly composed of fibro-lamellar bone tissue without lines of arrested growth (Fig. 6a). The medullary cavity is rimmed by a thin layer of avascular lamellar bone (the inner circumferential layer, ICL). The cortex external to the ICL is well vascularized by longitudinally oriented canals with local occurrences of radial and circumferential anastomoses. The fibrillar organization grades into a parallel orientation towards the periosteum in the outer quarter of the cortex, attested to the parallel organized and flattened osteocyte lacunae. The cortex of the femur is histologically similar to that of the humerus, except that the vascularization is lower and simpler, and secondary osteons are developed (Fig. 6b). The ICL, the secondary osteons and the increasingly well-organized fibers and osteocyte lacunae close to the periosteum suggest that the growth rate had slowed substantially by the time of death^18^. Combined with the osteological marks, particularly the complete fusion of the compound bones, the holotype of *Chongmingia* is inferred to be an adult or near adult individual.

When compared to the femoral histology of *Archaeopteryx* and *Jeholornis*^21^, that of STM9-9 is more vascularized, and the main cortex is composed of fibro-lamellar bone tissues, indicating a faster growth rate. The body mass of STM9-9 is estimated to be 290.2 g (using the equation in ref. 22), nearly equivalent to that of *Jeholornis prima* IVPP (Institute of Vertebrate Paleontology and Paleoanthropology) V13353 (285.4 g) sampled in a previous study^21^. Therefore, the differences among histological microstructures likely are not the result of disparity in body size, and that the growth rate was relatively elevated in STM9-9. However, it appears that STM9-9 grew slower than *Confuciusornis* and *Sapeornis*, who exhibit richly vascularized fibro-lamellar bone^23^,^24^.

## Discussion

*Chongmingia* is referable to non-ornithothoracine birds in having following primitive features: the furcula is robust and boomerang-shaped; the scapula and coracoid are fused; the coracoid lacks the procoracoid process; the alular metacarpal and digit are relative long; and metatarsal V is present. *Chongmingia* is distinguishable from the known non-ornithothoracine avialans by the unique combined morphologies of pectoral girdle, forelimb, foot and limb proportions. More specifically, the coracoid and scapula are fused in *Chongmingia*, a feature otherwise reported only in *Confuciusornis* among non-ornithothoracine avialans (Fig. 2); the proximal end of the humerus is concave centrally, in contrast to the convex condition in other non-ornithothoracine avialans (Fig. 3); the alular metacarpal is proportionally longer than in *Archaeopteryx*, *Jeholornis* and *Sapeornis* (Supplementary Table S2); as in *Jeholornis*, the minor metacarpal is strongly bowed, a feature absent in *Archaeopteryx*, *Sapeornis* and *Confuciusornis* (Fig. 4); as in *Sapeornis*, the penultimate phalanx of the major digit is shorter than its preceding phalanx, but the opposite is true in *Archaeopteryx*, *Jeholornis* and *Confuciusornis* (Fig. 4); compared with *Jeholornis* and *Sapeornis*, *Chongmingia* has a proportionally shorter forelimb (Supplementary Table S2), and the pedal phalangeal proportions are also different from other non-ornithothoracine avialans (see Supplementary Information for complete differential diagnosis of *Chongmingia* compared with other basal avialans).

In order to investigate the systematic position of *Chongmingia*, we performed two separate phylogenetic analyses. First, we added *Chongmingia* into the largest published coelurosaurian matrix (104 taxa and 991 characters; Supplementary Information)^9^, and the results from analysis of that matrix place *Chongmingia* within the Avialae (Supplementary Fig. S2). In that first analysis, *Chongmingia*, *Confuciusornis*, *Sapeornis* form the consecutive outgroups to Ornithothoraces. Given that only 18 widely accepted Mesozoic avialans are included in that coelurosaurian matrix, we also use a recently published comprehensive data matrix that targets the phylogeny of Mesozoic birds in order to refine the position of *Chongmingia* among birds (59 taxa and 262 characters, 58 Mesozoic avialans; Supplementary Information)^4^. The result of analysis of that second matrix places *Chongmingia* as the most basal bird other than the iconic ‘Urvogel’ *Archaeopteryx* (Supplementary Figs S3 and S4). The inconsistent (but broadly similar) systematic positions of *Chongmingia* in these two analyses likely result from the different taxa and characters sampled in those data matrices. In the coelurosaurian matrix, non-avian dinosaurs are the main component, and the non-ornithothoracine avialans (such as Jeholornithiformes) are extensively sampled. The smaller Mesozoic avian matrix includes more ornithothoracine taxa. Furthermore, the holotype and the only known specimen of *Chongmingia* is incomplete. In particular, the skull and the most caudal vertebrae are missing, and many phylogenetically important characters cannot be scored or discussed relative to the phylogenetic position of STM9-9 within the Avialae. Therefore, the phylogenetic hypothesis that *Chongmingia* is the most basal Cretaceous bird should be treated with caution, but clearly it is one of the most primitive birds known. Furthermore, homoplasy characterizing early avian evolution^5^,^8^,^25^, given the low support value Bremer and Bootstrap values (Supplementary Fig. S4), likely also played a role in the conflicting phylogenetic hypotheses of *Chongmingia.* For example, the manual digits are reconstructed as having been reduced independently in different avialan lineages^26^; and metatarsal V was secondarily developed in the derived ornithothoracines *Pengornis* and *Vorona*^27^,^28^. Consequently, when different taxa and characters are considered, these homoplasies bring different effects to the resultant phylogenetic tree.

**Figure 7:**
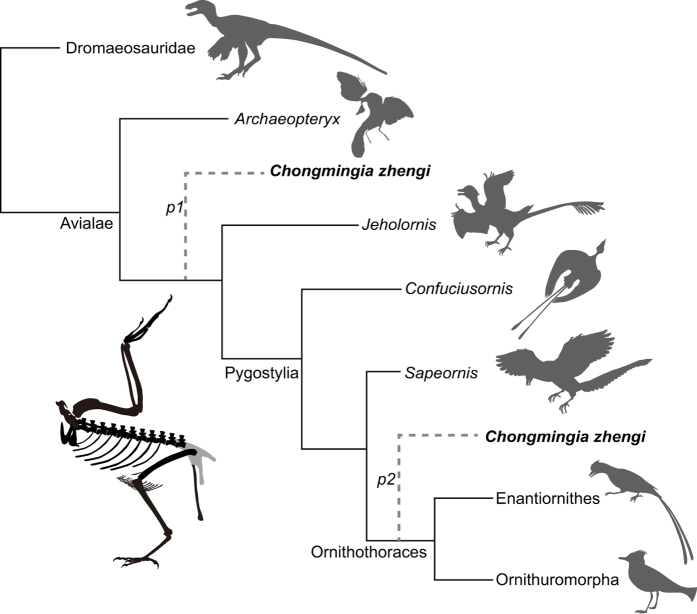
Simplified Mesozoic avian cladogram showing the possible phylogenetic positions of *Chongmingia zhengi*. Analysis using the coelurosaurian matrix places *Chongmingia* within basal avialans and as the sister group to Ornithothoraces (*p1*), and analysis using the Mesozoic avian matrix resolves *Chongmingia* as the most basal avialan, except for *Archaeopteryx* (*p2*). See Supplementary Figs 2–4 for complete results. (The skeletal drawing and silhouettes were drawn by Min Wang).

Despite those conflicts and absences of data, both phylogenetic analyses support non-ornithothoracine avialan affinity for *Chongmingia* (Fig. 7), and thus further increase the taxonomic diversity of basal avialans. The reconstructed basal position of *Chongmingia* relative to other Jehol birds is unsurprising given the suit of primitive features it exhibits, including the fused coracoid and scapula, the absence of procoracoid and extensor processes, the elongate alular digit and the presence of metatarsal V. However, *Chongmingia* exhibits several derived features, particularly the small interclavicular angle that is intermediate between Ornithothoraces and basalmost birds such as *Archaeopteryx*. The skeleton of *Chongmingia* highlights the mosaic evolution in early avian history, and demonstrates that the early evolution of birds was complex and homoplastic.

The Early Cretaceous is a critical temporal interval that records the early diversification of birds with numerous morphological changes pertaining to the demands of powered flight^29^,^30^. The fused scapula and coracoid (scapulocoracoid) is widely distributed in non-avian dinosaurs (e.g., dromaeosaurids, oviraptorids^31^), pterosaurs and amphibians^32^, but it has been reported previously only in *Confuciusornis* among Mesozoic birds (Fig. 2b–d). The co-occurrence of a scapulocoracoid in *Chongmingia* and *Confuciusornis* likely is the result of convergence. Among living birds, the scapulocoracoid is present in the flightless ratite paleognathous birds such as kiwi, ostrich and rhea. However in those large bodied flightless birds (i.e., the ratites), the scapular blade is generally in line with the axis of the coracoid, unlike the 90° present in *Chongmingia*, that is more similar to the state present in extant volant birds that fuse the scapula and coracoid. Generally the fusion between scapula and coracoid has been considered as related to a reduction of flight capability^33^,^34^. However, all pterosaurs were competent flyers with a fused scapulocoracoid. The strikingly different musculoskeletal configuration and kinematics between birds and pterosaurs prevent direct comparison^32^, and future mechanical study is needed to clarify to what degree (and even if) the fused scapulocoracoid is related to reduced flight capability in birds.

The furcula in STM9-9 is considerably more robust than in some non-avian dinosaurs (e.g., *Velociraptor* and *Microraptor*), *Archaeopteryx* and more advanced birds where this bone is delicate and U-shaped (Fig. 2a,e–j)^4^,^7^,^35^. X-ray cineradiographic studies of living birds reveal that the furcula, acting as a spring, experiences dramatic deformation during both the downstroke and upstroke^36^. The heavily built furcula in *Chongmingia* should have increased its rigidity. Along with the absence of an extensor process and a procoracoid process, and the fused scapula and coracoid, the robust furcula could suggest an overall lessened flight capability for *Chongmingia*.

However, refined flight-related characters also are present in *Chongmingia*, including the elongation of the forelimb, and the expanded deltopectoral crest which provides the attachment of flight muscles (the M. pectoralis and cranial head of the M. deltoideus major^37^). Those features are also present in other basal and presumably volant birds such as *Jeholornis*, *Sapeornis* and *Confuciusornis*. Moreover, the strongly caudally bowed minor metacarpal is known only in *Jeholornis* among basal avialans, but is widely distributed among extant birds (Fig. 4c–g)^10^,^37^. The bowed minor metacarpal provides better support for the primary feathers (that are attached to the major metacarpal), allowing them to sustain higher lift forces in high speed flight without structural failure (i.e., the bowed minor metacarpal increases the metacarpal’s lever arm, and in turn increases the lift force; Fig. 4h)^38^. All of these morphologies may have compensated for the lack of other flight-related characters, highlighting the evolutionary experimentations that likely occurred during the early evolution of flight among birds and their closest relatives^39^,^40^.

Approximately 17 stones (interpreted as gastroliths) are preserved in STM9-9 (Fig. 1, Supplementary Fig. S1), and are scattered and distributed in the chest region, rather than being aggregated in the belly. All of the stones are restricted to the body cavity, making it unlikely to be the preservational artefact (no stones are present outside of the skeleton on the whole slab). The disarticulation of the skeleton indicates that this individual underwent transportation prior to burial, and thus the gastroliths were likely displaced after the gizzard had been ruptured or decomposed. The stones are composed of quartz and vary greatly in size (4.6–7.6 mm in diameter), and vary in shape from subspherical to oblong. Stomach contents reveal that *Jeholornis* and *Sapeornis* were herbivorous^10^,^41^, and the diets of *Archaeopteryx* and *Confuciusornis* are at best speculative^5^. In herbivorous birds that consume seeds, the gizzard is muscular and contains numerous gastroliths responsible for grinding the hard food items^42^. However in carnivorous, insectivorous or frugivorous birds, the gizzard is less muscular and rarely contains gastroliths^42^,^43^. In specialized diving birds such as cormorants and penguins, gastroliths help regulate balance and buoyancy during swimming and diving^44^. Skeletal morphologies indicative of swimming or diving adaption are absent in *Chongmingia*, and likely mean that the gastroliths are only related to diet. Gastroliths have been reported in many non-avian dinosaurs and fossil birds, and are generally considered an indication of herbivory^45^,^46^,^47^,^48^,^49^,^50^. In addition, the general morphology of gastroliths of *Chongmingia* is consistent with that reported in the Jehol ornithuromorph *Hongshanornis*^48^, who was an herbivore given that one specimen preserves seeds in its crop^41^. If our interpretation is correct, the discovery of *Chongmingia* further confirms that herbivory (or at least a gizzard containing gastroliths) was very common among early birds^3^,^41^. In addition to pterosaurs^51^, a few small body-sized non-avian theropods were arboreal and even consumed early birds^15^,^52^,^53^. Therefore, the herbivory may have reduced competition with contemporaneous non-avian theropods and pterosaurs (both carnivorous), allowing early birds to diversify in a niche distinguishable from that of carnivorous contemporaries.

## Methods

### Histological preparation of *Chongmingia zhengi* (STM9-9)

Cross sections were prepared using the standard methods^54^. Bone samples near the mid-diaphysis of the right humerus and femur of STM9-9 were removed. Samples were embedded in one-component resin (EXAKT Technovit 7200), and hardened in a light polymerization device (EXAKT 520). Histological sections were cut transversely using an accurate circular saw (EXAKT 300CP). Sections were ground using the EXAKT 400CS grinding system until the desired optical contrast was obtained. The histological sections were examined by light microscopy under both normal and polarized light (ZEISS AX10). Images of each slice were captured using a digital camera (ZEISS AxioCam MRc5).

### Phylogenetic analysis

We performed two separate numerical phylogenetic analyses to investigate the systematic position of *Chongmingia*. First, to confirm its avialan affinity, we added *Chongmingia* to a recently published data matrix on coelurosaurian phylogeny^9^,^55^. The revised data matrix consists of 104 taxa and 991 characters (Supplementary Information). Secondly, we added *Chongmingia* to a comprehensive data matrix that targets Mesozoic bird phylogeny^4^, and the revised matrix consists of 59 taxa and 262 characters (Supplementary Information).

Phylogenetic analyses were performed using the TNT software package^56^, with following settings: space for 10000 trees was set in the memory as the maxtrees; all characters were equally weighted; unconstrained heuristic search starting with Wagner trees were used; 1000 replicates of random stepwise addition (branch swapping: tree-bisection-reconnection, TBR) were performed and ten trees were kept at each step. Branches were collapsed to create polytomies if the minimum branch length equals zero. Bremer and Bootstrap values were calculated as indices of support. Bremer values were calculated by using Bremer scripts embedded in TNT. Bootstrap value was retained by 1000 replicates using the same setting in the primary search.

For the first analysis using the coelurosaurian matrix, the analysis produced 630 most parsimonious trees of 4523 steps (Consistency index = 0.266, Retention index = 0.578). The strict consensus tree placed *Chongmingia* within basal Avialae, and *Chongmingia* is the sister taxon of Ornithothoraces (Supplementary Fig. S2).

For the second analysis focusing on phylogeny of Mesozoic birds, the analysis produced four most parsimonious trees of 1009 steps (Consistency index = 0.363, Retention index = 0.683). The four most parsimonious trees are in Supplementary Fig. S3. The new topology is well resolved and largely consistent with recent results with regards to the placements of major clades^4^,^18^,^57^. The strict consensus tree places *Chongmingia* as the sister to all avialans except for *Archaeopteryx*, and thus *Chongmingia* represents the most primitive bird from the Jehol Biota uncovered to date and one of the most primitive Cretaceous birds known. However, this phylogenetic hypothesis was weakly supported by both Bremer and Bootstrap values (Supplementary Fig. S4).

### Data archiving

Additional anatomical description, differential diagnosis of *Chongmingia* compared with other basal avialans, skeletal measurements, complete character scorings for STM9-9, and the resultant phylogenetic topologies are provided in the Supplementary Information.

## Additional Information

**How to cite this article**: Wang, M. *et al.* A new basal bird from China with implications for morphological diversity in early birds. *Sci. Rep.* **6**, 19700; doi: 10.1038/srep19700 (2016).

## Supplementary Material

Supplementary Information
