# Supplementary material for: A new basal bird from China with implications for morphological diversity in early birds
Source: Sci Rep. 2016 Jan 25;6:19700. doi: 10.1038/srep19700 (PMC4726217; doi:10.1038/srep19700)
Supplement: Supplementary Information [file srep19700-s1.pdf]

Supplementary Information for:

A new basal bird from China with implications for morphological diversity in early birds

Min Wang, Xiaoli Wang, Yan Wang & Zhonghe Zhou

Supplementary information includes following files:

1. Phylogenetic nomenclature

2. Additional anatomical description of STM9-9

3. Differential diagnosis of STM9-9

4. Supplementary Table S1

5. Supplementary Table S2

6. Supplementary Figures

7. Phylogenetic analyses

8. Supplementary references

1. Phylogenetic nomenclature

In this paper, definitions of higher-level theropod taxa follows ref.1,2,3

-**Avialae**, the most inclusive clade containing *Passer domesticus* Linnaeus, 1758 but not *Dromaeosaurus albertensis* Matthew and Brown, 1922 or *Troodon formosus* Leidy, 1956.

-**Scansoriopterygidae**, the least inclusive clade including *Epidendrosaurus ninchengensis* Zhang, Zhou and Xu, 2002 and *Epidexipteryx hui* Zhang, Zhou, Xu, Wang and Sullivan, 2008.

-**Pygostylia**, the most inclusive clade containing *Confuciusornis sanctus* Hou, Zhou, Gu and Zhang, 1995 but not *Jeholornis prima* Zhou and Zhang, 2002.

-**Ornithothoraces**, the most inclusive clade containing *Iberomesornis romerali* Sanz and Bonaparte, 1992 but not *Sapeornis chaoyangensis* Zhou and Zhang, 2002 or *Confuciusornis sanctus* Hou, Zhou, Gu and Zhang, 1995.

-**Enantiornithes**, the most inclusive clade containing *Sinornis santensis* Sereno and Rao, 1992 but not *Passer domesticus* Linnaeus, 1758.

-**Ornithuromorpha**, the most inclusive clade containing *Passer domesticus* Linnaeus, 1758 but not *Sinornis santensis* Sereno and Rao, 1992.

2. Additional anatomical description of STM9-9

The vertebral column is poorly preserved and incomplete. The neural spine is fused to the centrum (Supplementary Fig. S1a). The cervical vertebral foramen is more than half the vertical height of the centrum. The synsacrum is estimated to comprise 7–8

vertebrae. The prezygapophyses of the first sacral vertebra projects further cranially than its centrum (Supplementary Fig. S1b). Two free caudal vertebrae are recognized (Supplementary Fig. S1c). The caudal vertebrae are considerably shorter in proportional terms than in *Archaeopteryx* and *Jeholornis*. The transverse processes are caudolaterally directed, exceeding the width of the centrum in length. The prezygapophyses project beyond the cranial articular facet by a distance approximately half the centrum length. At least five pairs of gastralia are present. The bones are slender than the preserved ribs, curved, and taper at their cranial and caudal ends.

The furcula is more robust than in many other basal avialans and more advanced taxa such as enantiornithines and ornithuromorphs. As in *Archaeopteryx*, *Xiaotingia* and *Confuciusornis*[1,4,5], the furcula is boomerang-shaped without a hypocleidium, but the interclavicular angle is smaller. The bone is weakly curved craniocaudally. As in other basal birds, the acromion process is absent[4]. The left coracoid and scapula is exposed in medial view and jointed at an angle of 90 °. The scapula is fused to the shoulder end of the coracoid. Whether the coracoid is strut-like cannot be determined.

The forelimb is slightly longer than the hindlimb as in *Confuciusornis*, but proportionally shorter than in *Jeholornis* and *Sapeornis* (Supplementary Table S1 and S2)[6]. The humerus is stouter than in *Archaeopteryx*. In contrast to the condition of non-avian theropods such as *Xiaotingia*[1,7], the humerus is longer than the femur as in most Mesozoic birds[4,8,9]. The humerus is curved over its proximal half and straight distally, without the sigmoid shape present in enantiornithines and ornithuromorphs.

The deltopectoral crest approaches the width of the mid-shaft of the humerus. The ventral tubercle is not developed. The left humerus is exposed in dorsal aspect, reveling that the dorsal condyle is primarily located on the cranial surface.

The ulna is approximately of the same length as the humerus. The shaft is bowed over its proximal fourth length and straight distally. An olecranon is absent. As in other basal birds, no quill knobs for attachment of the second remiges are developed[4,5,10]. The radius is straight and robust, and its midshaft width is approximately 83% that of the ulna, proportionally wider than in *Jeholornis*, *Sapeornis* and *Confuciusornis*. The ulnare is rectangular with littler differentiation into the ventral and dorsal rami.

The major and minor metacarpals are fused proximally to each other and to the semilunate carpal. The alular metacarpal is only proximally fused to the major metacarpal. As in *Archaeopteryx* and *Sapeornis*, the cranial margin of the alular metacarpal is slightly concave over its central portion, but the proximal and distal parts of the cranial margin are level in cranial projection. Whereas, the proximocranial margin surpasses the distal articular facet in cranial extend in *Jeholornis* and *Confuciusornis*[5,11]. The extensor process is not developed. The alular metacarpal is longer relative to the carpometacarpus than in other basal birds. The major metacarpal is robust and straight. The minor metacarpal is strongly bowed caudally, forming a wide intermetacarpal space with the major metacarpal, resembling that of *Jeholornis*. The first phalanx of the alular digit is slender and gently curved as in *Jeholornis*. Although disarticulated, the preserved length indicates that this phalanx reaches the

distal end of the major metacarpal as in the basalmost enantiornithine *Protopteryx*[12], but shorter than in *Archaeopteryx* and *Confuciusornis*[4,5]; by contrast, it terminates proximal to the major metacarpal in *Jeholornis* and *Sapeornis*[6,11,13]. The first phalanx of the major digit is straight and robust, close to the craniocaudal width of the major metacarpal. As in *Sapeornis* and more advanced birds such as enantiornithines and ornithuromorphs[14], the penultimate phalanx of the major digit is shorter than the proximal phalanx; by contrast, the proximal phalanx is longer in most non-avian theropods and basal avialans, including *Archaeopteryx*, *Jeholornis* and *Confuciusornis*[1,15]. One manual claw is preserved closed to the major metacarpal. The bone is recurved with well developed flexor process. Its affinity cannot be ascertained.

The pubes are retroverted and have an oval cross section. Distally, the shafts converge and form the symphysis; although the preserved symphysis is short, the braded medial surface indicates that the symphysis is longer during life. A pubic boot is absent.

The femur is approximately 88% the length of the tibiotarsus. The bone is gently bowed craniocaudally. Proximally, the femoral head protrudes medially, and a distinct neck is not developed. The femoral head is badly preserved, preventing the observation of the pit-shape fossa for the capital ligament. The patellar groove is lacking.

Contrary to non-avian dinosaurs and basal avialans such as *Archaeopteryx* and *Jeholornis*[11,16], the calcaneum and astragalus are fused to the tibia, forming a true

tibiotarsus as in *Confuciusornis*, *Sapeornis* and more advanced birds. The fibula is more than 70% the length of the tibiotarsus, failing to contact with the proximal tarsals as in non-ornithothoracine birds except *Archaeopteryx*[17]. The proximal end is expanded and the shaft rapidly narrows distally to a splint form.

As in *Jeholornis*, *Sapeornis*, *Confuciusornis* and enantiornithines[5,6,11,18], metatarsals II–IV are fused proximal to each other and to the distal tarsals, but the three major metatarsals are separated along their distal lengths. As in *Archaeopteryx*, *Jeholornis*, *Confuciusornis* and *Sapeornis*, metatarsal V is present. The proximal margin of the tarsometatarsus is straight, and an intercondylar eminence is absent. The proximal articular surface of the tarsometatarsus is wider than the combined width of metatarsals II–IV at the proximal end. The tarsometatarsus narrows bellow the proximal margin, and reach its narrowest point one fourth of the distance from the distal end; at this point, the shaft begins to widen. Metatarsal III is the longest, closely followed by metatarsal IV, which projects distally well beyond the proximal margin of the metatarsal III trochlea. As in *Archaeopteryx* and *Confuciusornis*, metatarsal II is shorter than metatarsal IV. Whereas in *Jeholornis* and *Sapeornis*[6,14], these two bones are subequal in length.

The left foot is complete and remains in loose articulation. The third pedal digit is the longest and approximately of the same length as the tarsometatarsus. The second digit is slightly longer than the fourth digit as in some enantiornithines[10], contrary to *Archaeopteryx*, *Jeholornis* and *Confuciusornis*[5,11,13]. The non-ungual phalanges are spool-shaped with well formed distal trochlea. The non-ungual phalanges of the

fourth digit are shorter than these of other digits. Phalanx IV-1 is longer than its following three phalanges, which are subequal in length. The unguals are recurved, with well developed flexor tubercles, and excavated laterally by the neurovascular sulci.

3. Differential diagnosis of *Chongmingia*

Chongmingia differs from *Archaeopteryx* in having a robust furcula with a smaller interclavicular angle (68 ° compared to 83 °), a fused scapulocoracoid, a robust humerus with a large deltopectoral crest, an alular metacarpal proportionally longer, a strongly bowed minor metacarpal, a proximal phalanx of the alular digit that is subequal to the major metacarpal in distal projection, a penultimate phalanx of the major digit that is shorter than its preceding phalanx, the calcaneum and astragalus that are fused to the tibia, and the distal tarsals that are fused to the proximal ends of metatarsals II–IV.

Chongmingia differs from Jeholornithiformes in following features: the scapula and coracoid are fused; the procoracoid process is lacking, but present in *Jeholornis curvipes*[13]; the furcula is robust than in *Jeholornis prima* and *J. curvipes*; the proximal margin of the humerus is concave, a feature absent in *J. prima* and *J. curvipes*; the mid-shaft width ratio of radius and ulna is 0.83, compared to 0.64 in *J. prima* and 0.54 in *J. curvipes*; the alular metacarpal is proximally fused with the major metacarpal, but completely unfused in *J. prima* and *J. curvipes*; the alular metacarpal is long relative to the major metacarpal, with a length ratio of 0.32, compared to 0.25 in *J.*

prima and 0.17 in *J. curvipes*, respectively; the proximal alular digit extends to the distal end of the major metacarpal, which terminates proximally to the distal end of the major metacarpal in *J. prima* and *J. curvipes*; the proximal phalanx of the major digit is longer than the penultimate phalanx of the same digit, but the opposite is true in *J. prima* and *J. curvipes*; the proximal tarsals are fused with the tibia, but are unfused in *J. prima*; the second pedal digit is longer than the fourth digit, in contrast to *J. prima* and *J. curvipes*; in *J. curvipes* the penultimate phalanx is longer than other phalanx, but this feature is absent in *Chongmingia*; the ratio of forelimb (humerus + ulna + the major metacarpus length) to hindlimb (femur + tibiotarsus + metatarsal III) is 1.07, compared to *J. prima* (1.3), *J. curvipes* (1.23), and *J. palmapenis* (1.2[19]).

Chongmingia is distinguishable from *Jixiangornis orientalis* in following features: the forelimb is proportionally shorter than in *J. orientalis* (the length ratio of forelimb to hindlimb is about 1.34); the proximal margin of the humerus is concave in *Chongmingia*, but is convex in *J. orientalis*; the proximal phalanx of the alular digit fails to reach the distal end of the major metacarpus in *J. orientalis*; the penultimate phalanx of the major digit is longer than its preceding one in *Chongmingia*, but the opposite is true in *J. orientalis*; the alular metacarpal is proximally fused with the major metacarpal in *Chongmingia* but not in *J. orientalis*; the pedal digit II is longer than pedal digit IV, whereas the fourth digit is longer in *J. orientalis*.

Chongmingia differs from *Sapeornis* in following features: the furcula lacks the hypocleidium; the interclavicular angle is smaller (68° versus 108°); the coracoid and scapula are fused; the humerus lacks the fenestra in the deltopectoral crest; the distal

margin of the deltopectoral crest is concave in *Sapeornis*, but not in *Chongmingia*; the forelimb is proportionally shorter (the forelimb are about 1.5 times the length of the hindlimb in *Sapeornis*); the minor metacarpal is strongly bowed in *Chongmingia*.

Chongmingia is distinguishable from *Confuciusornis* in having a robust furcula with a smaller interclavicular angle, a smaller humeral deltopectoral crest that is imperforated, a minor metacarpal that is strongly bowed caudally, a proximal phalanx of the alular digit that terminates to the distal end of the major metacarpal, and a proximal phalanx of the major digit that is longer than its following phalanx.

4. Supplementary Table S1. Selected measurements of *Chongmingia zhengi* gen. et sp. nov. holotype (STM9-9). Lengths are measured in millimetres.

Element	Length
Scapula	44.6
Humerus	72.6
Ulna	77.8
Radius	74.2
Alular metacarpal	9.6
Carpometacarpus	30.4
Alular digit-1	17.0
Major digit-1, 2	18.9, 15.7
Femur length	61.6
Tibiotarsus	69.4
Fibula	54.1
Metatarsal II	33.3
Metatarsal III	37.3
Metatarsal IV	35.6
Metatarsal V	4.1
Digit I-1, 2	9.5, -
Digit II-1, 2, 3	11.5, 12.1, 10.9
Digit III-1, 2, 3, 4	11.2, 9.6, 9.5, 9.3
Digit IV-1, 2, 3, 4, 5	7.3, 5.4, 4.8, 5.7, 7.6

5. Supplementary Table S2. Comparative measurements of *Chongmingia zhengi* gen. et sp. nov. and other basal avialans. Lengths are measured in millimetres.

Element	*Chongmingia zhengi* STM 9-9	*Archaeopteryx siemensii* WDC-CSG-100	*Jeholornis prima* IVPP V13353	*Jeholornis curvipes* YFGP-yb2	*Sapeornis chaoyangensis* IVPP V13276	*Confuciusornis sanctus* IVPP V13156
Hu	72.6	57.8	88.6	102	121.5	61.3
Ul	77.8	51.6	90.2	108	123.5	55.6
Al	9.6	6.9	9.6	10	13.6	8.4
Ma	30.4	25.1	46.2	59	59.3	30.5
Fe	61.6	51.4	63.4	76	73.2	53.4
Ti	69.4	76.5	73.7	94	85.1	60.4
Mt	37.3	40.7	36.1	48	41.2	31.3
A/M	0.32	0.27	0.21	0.17	0.23	0.28
F/H	1.07	0.80	1.30	1.23	1.52	1.01

Data of *Archaeopteryx siemensii* (WDC-CSG-100) is based on illustration in ref.4; data of *Jeholornis curvipes* (YFGP-yb2) is from ref.13, and the alular metacarpal is based on image therein; the rest data is measured directly from the corresponding specimens. Abbreviations: Al, alular metacarpal; Fe, femur; Hu, humerus; Ma, major metacarpal; Mt, metatarsal III; Ti, tibiotarsus; Ul, ulna; A/M, alular metacarpal length/ major metacarpal length; F/H, humerus + ulna + major metacarpal length/ femur + tibiotarsus + metatarsal III length.

6. Supplementary Figures

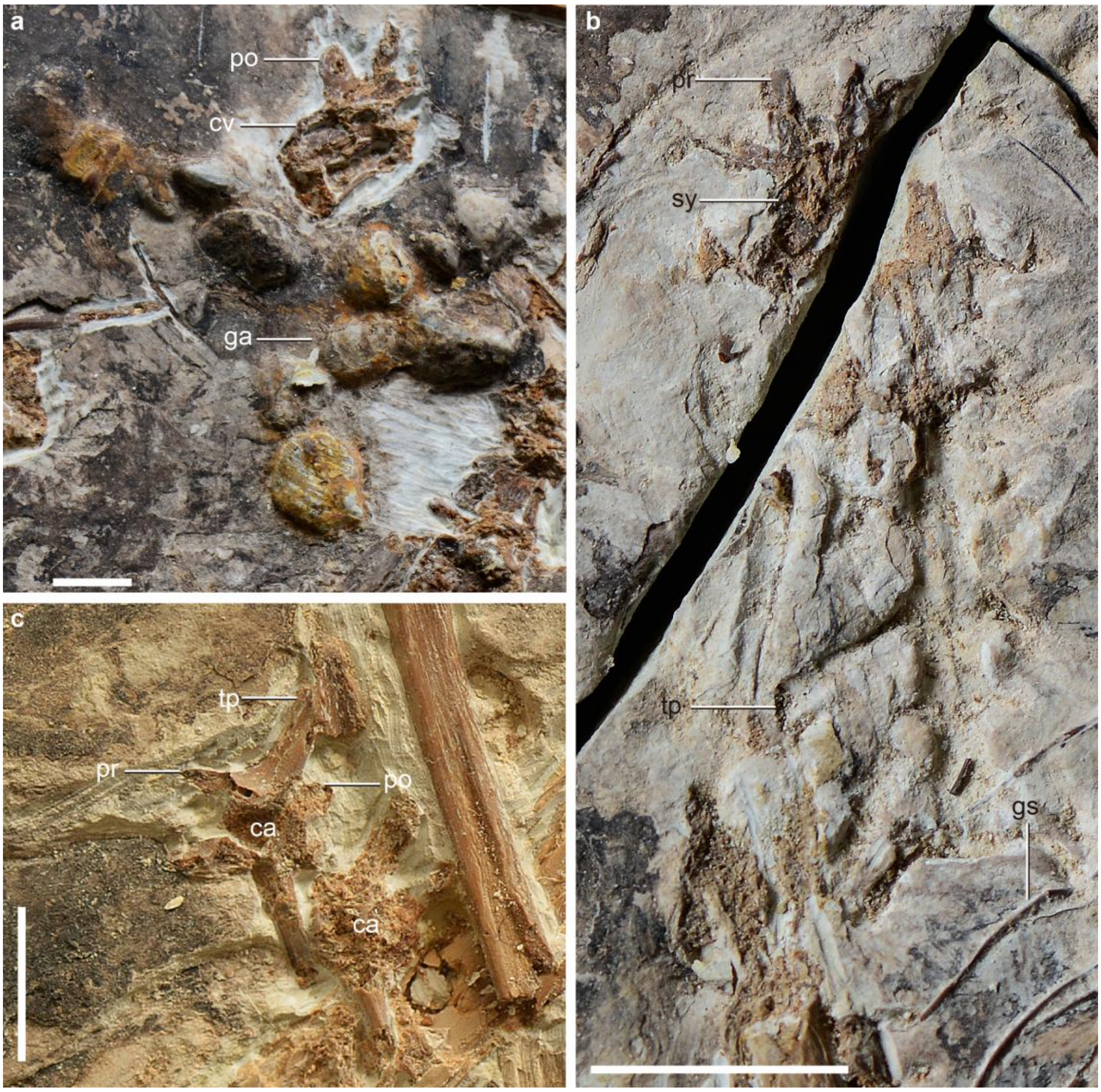

Figure S1. Axial skeletons of *Chongmingia zhengi* (STM9-9). (**a**) Cervical vertebra and gastroliths; (**b**) synsacrum; (**c**) caudal vertebrae. Abbreviations: ca, caudal vertebra; cv, cervical vertebra; ga, gastroliths; gs, gastralia; po, postzygapophysis; pr, prezygapophysis; sy, synsacrum; tp, transverse process. Scale bars, 5 mm (**a,c**), 10 mm (**b**).

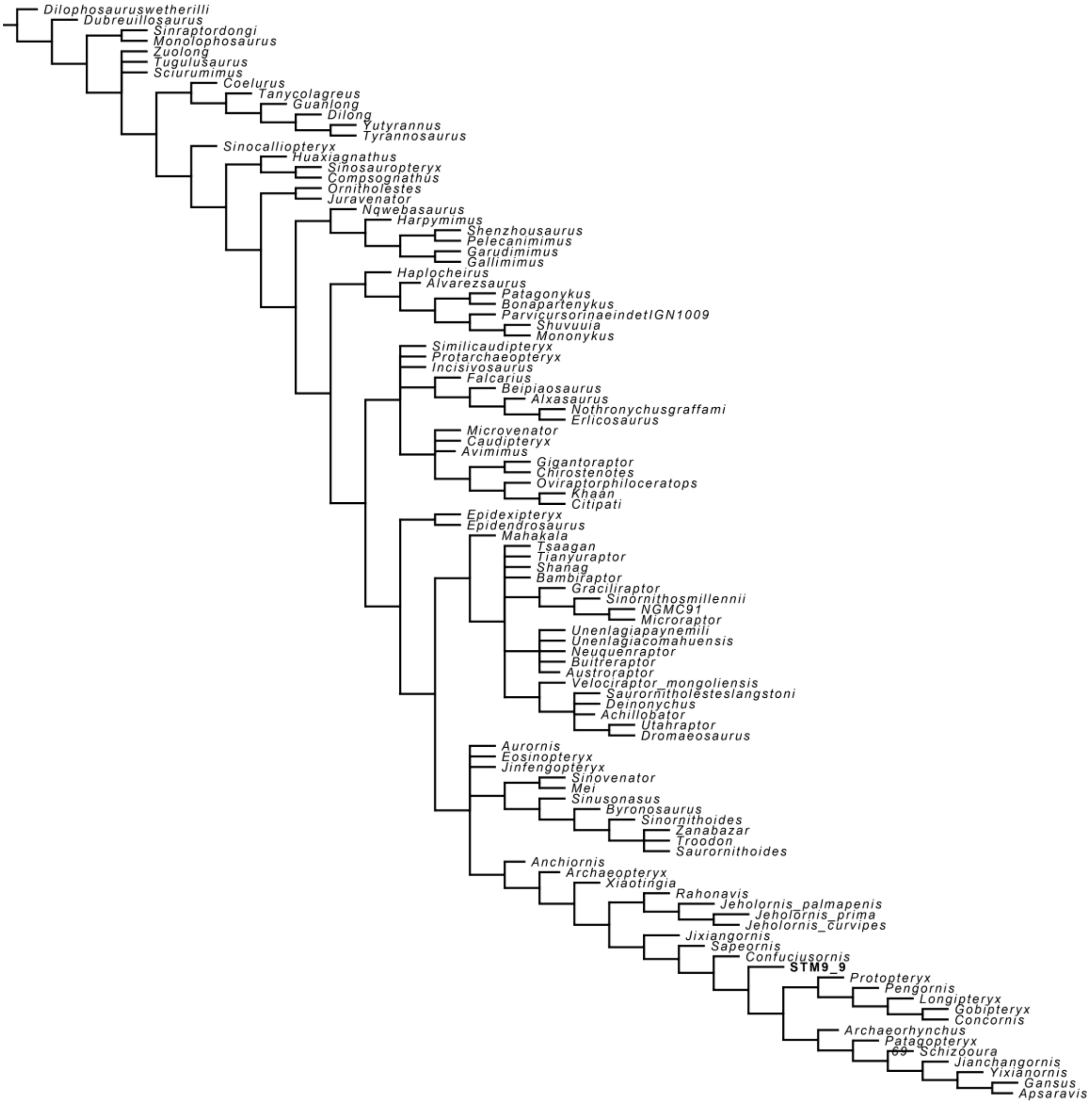

Supplementary Figure S2. The strict consensus tree of 630 most parsimonious trees of 4523 steps (Consistency index = 0.266, Retention index = 0.578) resulted from analysis with addition of STM9-9 to a matrix on coelurosaurian phylogeny[13].

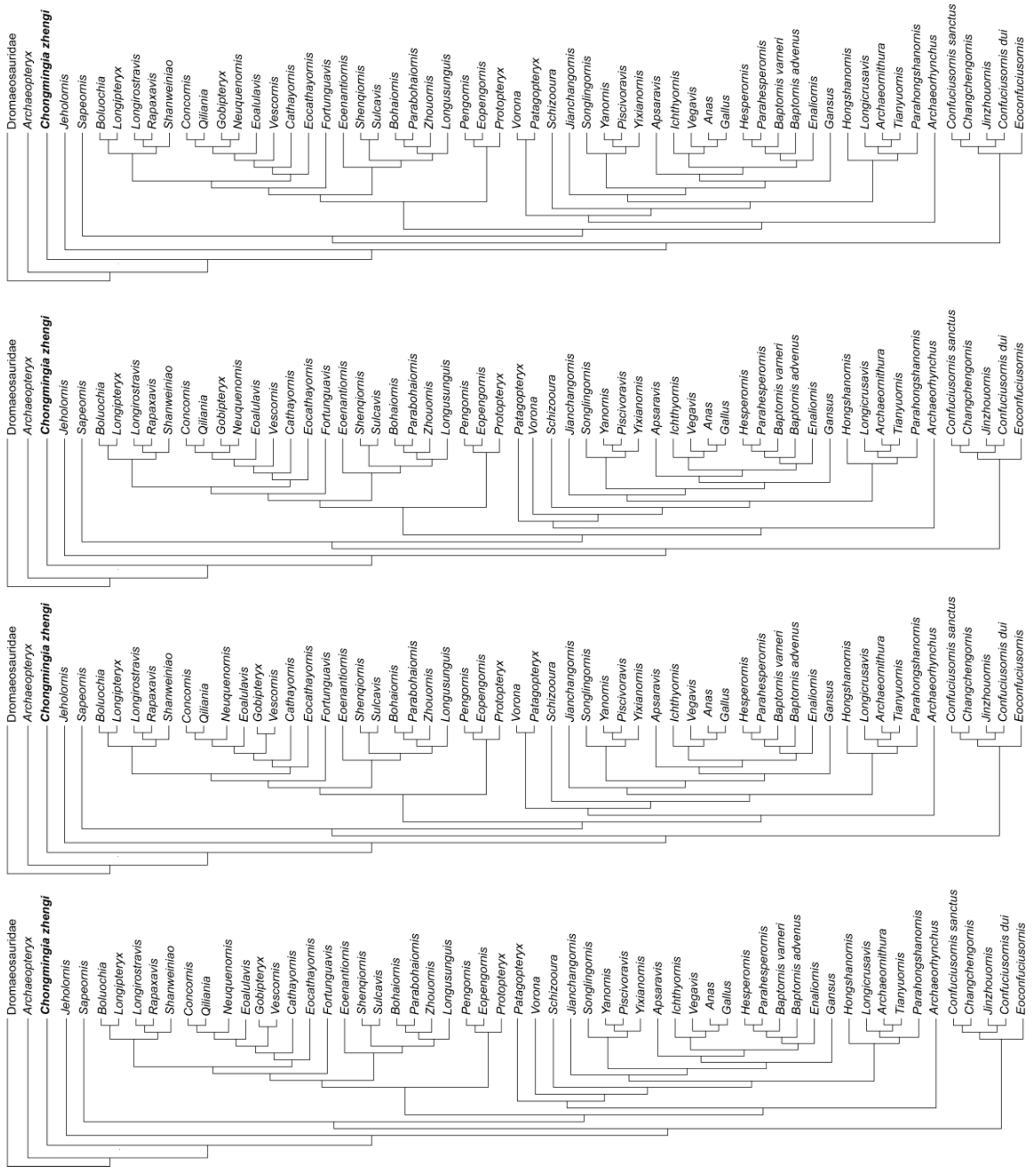

Supplementary Figure S3. The four most parsimonious trees (Tree length = 1009 steps; Consistency index = 0.363; Retention index = 0.683) resulted from analysis with addition of STM9-9 in a matrix on Mesozoic avian phylogeny[19].

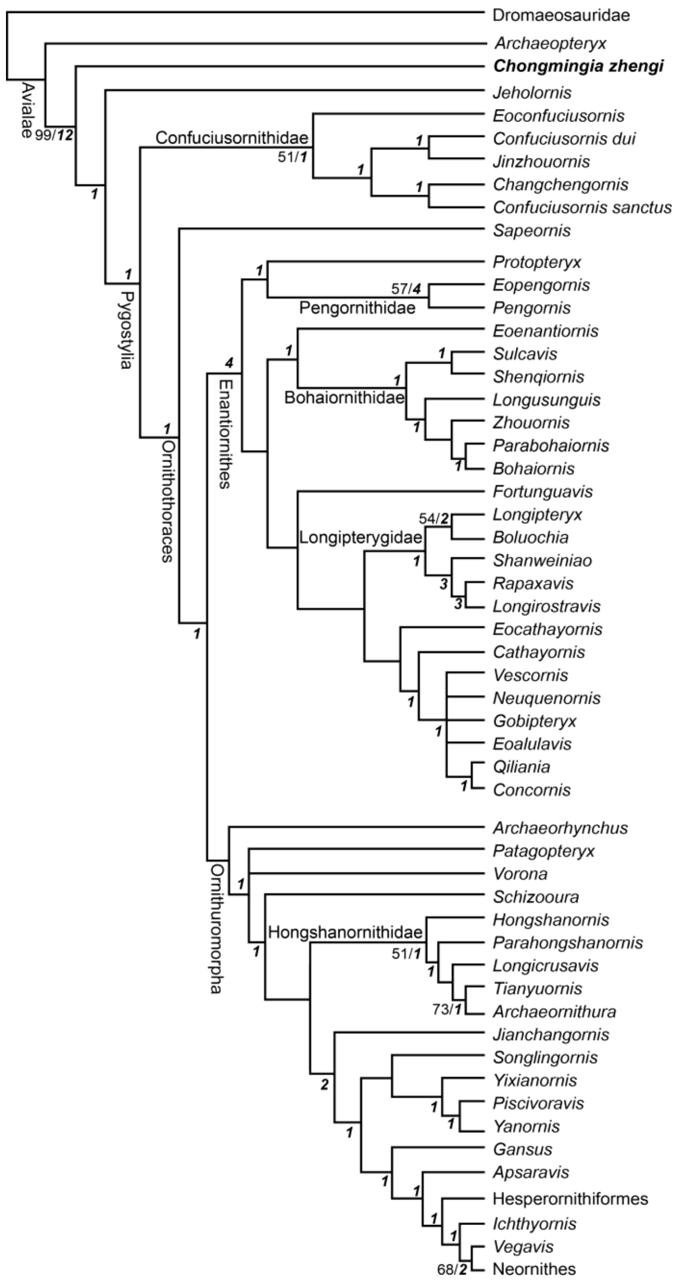

Supplementary Figure S4. The strict consensus tree of four most parsimonious trees of 1009 steps (Consistency index = 0.363, Retention index = 0.683) resulted from analysis with addition of *Chongmingia* to a matrix on Mesozoic avian phylogeny[19]. Bootstrap and Bremer values are spelled out near the corresponding nodes in normal and bold italic formats, respectively.

7. Phylogenetic analyses

Matrix on coelurosaurian phylogeny after ref.13.

Dilophosauruswetherilli
0010000100000101000100001000000000011002000??0?0?00101100[01]110101100??0???????1
0100110100010?0001[12]01000000011000?10000000002????0???1000?0000100001000000 1?01
1000????00001?0100??00000200112000000001?00?01021?00001010??10??0?0111000100000000
0010000000200000310000000 10??100001011??000000000110000000020100 1??????[23]000000
0?0?000010010010[01]?00?01111023?1??200100000?011?1000000001?001010000110010100 20
00000011100 12001110[01]00??01100000000000000 10?1??1100?0110????????0?00?001?000?100
00000?1000?0100?00000?000000?00?0?01?001?1000000000000000 10?0?0?00000?00?00?1101[
01]??001000100??200000000100???1?000110000000001?0000000?001012 1111100000111?10?01
00?0000?0000?10010100???000010?01?00000000??00?1100000200000?000??000?00001???00?
000???1000??01000?00???00000?0????0?0???11000?10??11?0?000?100?000001???0?0?01112?
100000000000?000?????0???????10??000??????1?00000001?00???0?0?10001?0?0000?0??0????
0?????00000000??0???000??100??100000000????0?0??00????00?????00????0????0000?0?0?0?
000?00?00?000001000?0???000??0?

Achillobator
0??????????????0???11?0????010[12]00011?0???
???10???00000??????????????????????????????
0?0??????????0020?1120010???00121??20???1???11?????2[12]?1001001110020010 00??????????
???????0???????????????000??1???0?????10??????????12??????????????0???4????00???1?0??1?101
00002110001 2010?210?12?10001?2200010101[01]01001102200110?0100?0?0?01???0???123??
0?00??01?1?11?00?0???????1?1?????????????0?01?????1??1???0???1?1????????0?10?0??0?1??1?0
010?0?1?????20??????0000?????1????0???00??2110????0??????0???1??2???????????????0??1?0
1??0?0?0?0???????????0??????????000??0????????????000??0?1??01?1?0????0???0??0??????0??0???
??00????0??0????00?0?????????1?????????0??00?0?0???0???????00????????00???0???????????????
????0?????????000?0?0?1????????????2?1????????00??0?0100???????????10100?0??????01?0????0
??1???0?0??????????0??0??????????????????????????00??????????????????11???????0?????00?0????00
00????????0????2????0?10????0??0?100?0?1?????2?00????0??????0?

Alvarezsaurus ??
???
????????????0?0????????????10000[12]100??0????11????????????????????2?100???????0?00????0
???001??????32000??0[12]?1?????000
110?0?001?0100000010?00[01]01??1?1111?00????????????????????????[12]20001??????0????????
0???10?[12]??00?[12]?010??0010?01000????100?01???????????1?????????????????????????0??????
???0?10?????????00???0?1????1?01?????0??0???0?0???????10?0???0??????0???????0????????2?????
??????0????????0??0??????????????????00???????????0????????????????????????????????1??0?1?????
???????00???????????????????????001????????????????????????0?????????????????1??0?0??????101????
0?1???????????0???1?????1??10??00??????????????????2???0?0???????0?0?010000?????????????11???
??????0????????????1?0?????????????0???????????????????????00????????????????0???0???????1???
???????????0?10??????????????1????000?????????00????????????????1??????0??????

Alxasaurus ??

??00101000?01011??011??????
0?????????????0?0???????????1???0?1?????????[12]1?01100001??10??0?01211?????????????1?
0?00102?0010?1???0??????010?011010??0??1???????01112?????210???????3101??0?????1???0
1???0?0????????0???1?????????1????0?0???????????????????1??????2??????10??000?0??0????
??????????0?00?0?????????0????????????????????0?????????????????0??????????0???????????
0????????????1?0?0???0???0???0?2??????????0?????????0????00?00??0?????01???02????????2
1?010?????????0????????1?0??????010???????????0???11???01????0???0????00???1????????
1?00???????1???????????????????000?????1???00?????????0???0?0????0???0?0??????????????
??????????0????0?????0?????0???0???????????1??????0?010?000?00???010?????????????????
?????1??1?1?????????1??1????????????????????0???????????????0????0?????0?????????0????0
?00??1??00????12???0??0?????????????????????0?????0?????????0?
Anchiornis
10101?0??0???2??????1???1?????1??1??0?11?????????????????1???1??????????????????????????
??0???0??000??001?2?2???????????????1????????1????0?
????????????????????????????????????0????0?0??????????20???????11??2???????11?0??0??1????0?0
00100[12]1?11221???0101001212?0?121?????2?1??????0????????????3???1?11?0???0???00010
1110?0?020011?11?11?10????????????012????1????????1?????????????????2????????011???1?01
011???0??10?1?01?101?11110112?????01????00????10???00?1??????????1?0?????????????1???
?????0?2??0???00???1??1??00???0???????????????????????2001???0??????????????0?0??[02]??
????????????1??????????????1??????????0????0??1?1???1?????00100????????0?0??00??????????
??????0???????10?????1??????0?00????0?????????0?????????0[12]????0??????????????0???????
???????0??????????0????????1?????0????111???01?0??0???1???0????0???????1?1?0?????????????
??????????????????1??0??????????0??0???????????????0????0?????????????101??0??0000??00?00
?????2?0??0????????0???1011?????0?0?2??11????0????0?
Apsaravis
0???
??1???000??12????????22???0?????0?????????????0?1
??????????100????????1?????1????????????????2????21111111?1101210202011110?11010 11111[0
1]1010?1021?11302????????1???1?????2???????2???101???8?20011?41??30?????0???0110??1100
00?[01]?????[01]?????01011?0?000?0221?2111000?1?010[12][12]00?12???2??0????????????????
?12??1110?10011?0111?????1001001??????????11?1???0?0??????0??000????????????0?0?????
?11???1????1??21?1???0?0???0??????0???100??????????????????????????2?01???????1?????????
2?000?020?????????0???0?0???2????????????????????????1??????????????0??1?????????????1???1
0?1??????????????????????????????0????1011????????????????0?1?1?????01?1??????1?????1?0??
0??????1111?????211??111?????0???2?1????10????0?0?1??1?1111?1???01?11???????????????0?
0?????????????????????????????1???????????????01?1??010???????0????????????1????????????1110?
???1?????????1????0????????1?111????????????0???1?????0??????
Archaeopteryx
001000010000?200?001100111??1020000110110001010011010000011111 00?1?000?0100101?0
?0???2001000?001?01[01]001100111010?0010?01012?1??010??0100100 20212?0?00000???11?1
00100?0001?0001??0??????0?010?102??10001?0?11??[01]??00110????0?0012011?111?1100200
101001101000101110001000100 21?112010?0010100120210?1011101?2001?????03??00??03122
30??10?1?0?000021000100 2000?01201112120111?0001?02100100[01]2[12]10110102[12]?0[01]
10??10?000???11012?01?23010 1100?1110011?0110120 1110100110110100120??1?0?000?000?0

?1011?001100[02]01??0?01?0100???0?1??001011??201002??11000002?110110000?002100010
?00?00?00?1000?2001?0000?0?????00?00?00000200000?0?0???101000?0?????[01]11101??0???
00000?0010111?0011000110 1100?0100??00001101?10211??00001?110?001??0000?001????100
001?0000?1110001000?0????00200000?001????00?0100000010?001???00?000?1??01?0?00112
01???0100?1?00000?11110??01??1???0????1000?1?1?00?00?1???00?????1?0??0??010?00?1101
121??1???0000000??0?0000??00001?0???0001????1101?00??10001010?000?1001?0?000100?0?
0???1011??1101?0120?11?0?00???000
Archaeorhynchus
0?1??2010110????????1???11??10??00?????[12]00??1????????0??????????????0???????????????
?????0?0???0??0?[01]?????????0??????????????????1????000??12????????0?????????????0?1????
?????????????????????????????010???????????????1??????0??201??111?1??02??2?1011101??010
111??0?010??021?1130????????1????11?0?121?????2???111[12]?15???01??4?????????????????1?
???????????????????????2?????????0?1??0?2???200???????????????????????????2?111????[12]3??1?
0???0?0?011?01?0???????0???1?1111?0???????1???0?0??????0??0?0?1???1???0????1?0????????
???????????10?2????????0????????1???0?????????????????0?????002?01????0??????????????0?0?
?2?0?00???0?????10?0????????0??1??????????0??????????????????0???11???0??????0?0?10?????
?1????1?????1??????????????????1??100??????????0???0???0?????01????????1?????0????0?????
??00?????????????1??????0??????1????1???1???????????1?????????????????????????????????????
????1????0????0????1?????????????1??1?1?00?????????0???????????????????????1?11??????10???
???????2??0????????1???1?1??????????02??1??0?????????
Austroraptor
20??????????????????1?0???????1121000?10?1??????????1011?1010?????0???121????01?????0100
???010010002202?1?[01]000???????10?????????
???0?0??????????10101102?0???0?01?11??????????????????1????????????????????????000?1??0??3?
???????1??12??
??1?????????????????[01]????2??00?1???1????
??????????????1?1???????????????1???0???????0?10?0???????????0??0??0????00?????????0????????
????2????????00???????0??10??????????????????????0?????????????1????0?????2??0???0??0??0?????
???????00?????????????????0??????????0?0??????????0????01??0?0???????????????11??????????0??
???0?????????20????????????????????????0???????????????????????????00?1???????????1?????????0???
???????11???????????????0?00?10?0?????????0????????1?0?10????01?????0???????????????????1????
0????0?????????????????0???0?????????????????0???1???????0????????0??????????????????????????????
0???????????????01?????????12???0???0???????
Avimimus
1?1?????101?????1?0????????10??????????????000????0???????????????0???00?11111101??00?02
1??0???0?00?0101001011210??10?1???02?2?????????????1?12???????????1??00010?0??2???11?0
10?0?1?01?101?0001011020???1?1?11[12]1?000??001??1??10?0021?00?????????1??1????0??[12
]?00113?100[01]??10???2????1??????????1???????0?????????????????????6100??1??0??????????????
00?0??11?0001010?0000100100?1011000112210000102000100012201120??10????1?01101200?
0231111100121100111011002???01000110???????????11?1??00?????0?00?0?00?????0??0???00?
1?????00???1??0?1?020??0??0?00??0???0?00?1?000?0????0????00???0????????210???????0???1
????010????????????????0????????0?0????010???????1?????????????0??????0????011?0110????0???
10???????????????1??0????????1??0?10????0??1??????0??0?0??0??0????????0??0???2?0????0?????
???????0??0?1?0??1?0????00???????????????2?1???0???1????????????????????????10?01????????0?

???0??0?110010?1????00?????0?0100??????????????1?0??????????????000???11?00??1??0????
1?11??????????????00002????????????????1????0????1??????1???00??????
Bambiraptor
000000010001?0?0?2?1101000101020?00010110001???01101000110111100110020101??10100
?0001200000??10010020??101?110?110?001110???????1???002000000[01]0000??000????10?0
11????0011??10?????1?1???1?2??1?21?10?01??11110[01]?0?011???????0022010?11111?0?2??[1
2]0100010000010211000[01]0000?0211112010??010100120210?1112?01?2112?0100031?1???0
21242??????1?2?0?0021?10000?10010120100210?12?10022?02100101021001001122001100 11
0000?0?01?0?2???023??010?1?01?1011?0100?001?01112010????????00?1?01001?010?000010?
021110001??0?00?0?00101001???010?1?0200001?000000002?1001?0000001?2111??0?00?00?0
001?1??2001?000011?0????01?01010002000?0???00?0??1000?00????0000?1??00??00000?0?00
0?0??011000111110?01????0?100110?00?101??0001???0?00110100?100?????11?00000?0??0?
10??10?0?????10010??00?00110???0?010???0001?001????1?0?0?1???01??0???201????1????0?0
0???????0????????1?1?????10000100?0?????1???????????????01?010????1????????????000000?
?0???000?1?0???????0?00????0010??00????00??00?00?1?????0?000100???0?00?10???1110??12
0??????00???000
Beipiaosaurus ??
??00101000?0??1???0?1???????
???????????????????????????????????02?00????????1??1?????1?????????2?10?????0?0?20?1?000
010????102?10?0?00?1?010?01101???001100020210?11211?2?2?02?????0????????20?????????
0?0001??0?0[12]0000010000011100?11?01000??????1?????????0????1?0??2????0??10?1?1?01
1????2??0010?0?0?????????01??1??10?????10??0?0?0?0??????????0?????????0???????2??????0??
????0???0???0?0????02000?0??00?00??2?0????000?0????01???0?????0??0?1???????1?1??0????
????2????0?????????0??????0?01????????????????????????????01???00????01???0?0???10?1????
?????10??????????????????0???????????0????????0?000???00????????0?00??????0?0???????????
???????????0?1??????????0?0?1???????0???1??????1????????????????0???????0??????0?1????0?0
???????1??110?????1????0???10????1?0?0??????1??0?0?0??????????????0????0?????00?????????
??????00??0??00??????0?0????????????????????0?0??0???????????????0?
Bonapartenykus ??
??
????????????0?????????????10?102?1?????0??1??0?00011????1??1?0????0010010???00?210???
???0????2????????????0?010?1????
?0?????????????????????0??????0??01?0?2??10????????????????????1?????????11??????????????
????????????????????????????0???0????????????????1???
???????0?????????????????????????0?0???????????????????????????0????0?0?????????????????
????????????????0??0?1??????????????????1??
???00????????????????????1??0?????????????????????0?21??????????????1??????????????????????????
???????????????????????????????10??0?0????????????????????1???????????????????????????????????
??????????????????????????0??????????????????????????00?????????????????????????????????????0???
??111?????0????????????????????????????????????
Buitreraptor
20??0???0????????????10????101?2[12]????200?00010?00?????????????1??0?1?12??0?1010??0??
?0120?100??????0??0?????????????????????????????????010100022202????000????????10???1???
?????0???1????1??1?10110?????1???1?11??0???01???1??0??01201111101110?2???2?1??01?0??0

?0211?00??11??02??????????????0?????????????????????????0?1?0?????1232???10???1?0?0011
0?01?02?0????????1?1??12?10?2?????00100121001001102001 1?0??0??0??????0?[12]????2??10
1?00?1111011?0??01???1???1?????????????01??01?0???0??00???1?12??????1?100???0?0??1???
???00?????0[12]00??1???0?1?0?????0??0???0?1?210?????0?1???????????20????0???10?????2??
0???0?0???0????????????0??????????????????????00????1???11???1?00??11110?01??????0??
00??1?????????11????00???10????0?????1??????0??0???????010?????1??2????0?0?????0?0???
?0????0????1????0?????1??01????1??2??????????11??1?01001?0001????100?10??1??1???110??
10?1?????0????1????0??0?0???????????????0??????????????????00????000??00??????00??0???
00????00????????1?0?0???0?????????1001????0???12???0????0?????0
Byronosaurus ?0011001000112001200 10?10110122000?20100001?000110100001?1????0
?????1??????????????????????????????01110???11?0??1?111012?????????010?00010212?0000
0000?????11????????1????0????????1????1???0[12]1??0????1?10?0?????0???11????????????????
??0????????
?????1?????0??10??0????1???
??????????????0????????????????????101????????????????????0???001000???????????0?1???????
10???????????0?0??0?21??00?????????0???????0??????????????????????????0?????????0?????????
?2?1??????????00?0???0???????0??????1??100???????????1??????????0?0???0?????????0?010?1???
?0?????00?10????0?????0?001????0??000??????????????????0??0?????????11??0???????1??0??????
?????????????????????0????????????????????????????1??0??????????????????10????????110???1??
0????????????????1????0??????????????????????0?????????????0???0??1?0????00????1?00??00?
?????1????????????????0???0???????????00???????002????????????????
Caudipteryx
1110?1111001?200??1011??11??1???0001001?001011?0110?100?0?1?1000???0???1110?0?000?
0012?0??0??10010010?????????????????????????????????0011?12????????11?1010???0200????1??
0??100?010????1????????1??0??0????0?[01]1??[01]0??001111??1?001211?0111?1?????01?0??0?
?1?????3???000000?0021?0?101?0?010100110210?111[01]?0??20010??00031?0?0??30121???00
1000?0010000100000110000201 1??11?11?10001?2210010 11200?1001?2?01?1???10??00?????0
1[12]?0??230001100?1110011?011002?1110000110110100100?01?010?0?00????1000?10?11?00
1??0??0?0?00????0???0?10????2000????00?00011?0????12000?0?210???0?00????00?110??200
1?0000????????????0?00??01?00?0???00?0?010?0???????01010???0????000??1??00?1?0?110001
00110???????????001000????11??00001?????001??????????01???1?000000??0????0??10?0?0????
0010???0?0?1???00?0?100?00010?????????0?0?0?1100?????0??2?1???01???0??10001000?0???0?
?010?0?00?10???1???0?????1?????????????????2?110????1?001?????????10000?0?????0?0??01??
??0??[01]001000??00111000?0?00??00?00?????2?0??0?00????0??0100???1????0010?11?0??0??
?00?
Chirostenotes ?????1??????????????0110???1110??0000001?2??????????????????????????????????
???????????????????????????????0101?2????10?11111 2?2????0???0000??120??????012101011
10200?1[01]?011?11100?0??????????0???110???10?1??11211 1??????????11????????????????10002
0?1000????????????????????1???1??0?21000??0??01?021??????1???2101??????41010011??????
??01??0?0???10?0100000110000200012121 11?10001?2?100?011200010010[12]201?1?1?1000??
???11??100??23000110011110011001100 2001??????1??????????0??????0???0???00???0?10????2
11???10???00????00?0?0?1????0200?0??0000?0??2?0??1?10000???21??????0???00???110??2?0
1?0??11??0?0??11?0?0???0?0?0?01?1?0?1?01??????1??01??0?????????0000?0?0?00???010??00?
0???0??????????0?010?????????????0?????0?0???????0????01?1??0?0???0?00?0??0?01??0?0??????

100????0?1?????????00???0?????1?10???0?0?1???????????2???????????0??????????????????1?
?0??0??????1??0?001111????0??????1??1???01001 1????????????????00??????????????20?011?1
11100111???????1????00?????00?0?002??????00????0???1?0????1?0??0??0??0???0????0?

Citipati
11020111111 20??11??01102101111??0001001020211000110110020111000000000011010101100
000010110000110100000010012100?100?1?01[01]?2110000110101??12???????1211010011020
00100112?10100?1?0???????1110?1010?10110?1??1?100??0??111111?1012110011?110002001?
0?001010?0002110?????00?021?11201???0101001201 10?10100?2?210?[01]?????41?1????2012
00??10?0?000001000100002110100201 01?011101100 11?2210010111000100002 20???????????
???????2???????????0?001??0011?01?002??????????1?????????0101?11000?00010?0000?0001?00
01??01??0000???0??0?10110?10?1??00??200??00???1?011100?0???200000???1???00?1?1???20
01?0000????????????0?0?0?00?00?01?111?0?01??0?????????10010???00?1?010?00?1001?0001?00
00?0010???00????0?01000????101?0?0?1???0?011?00011???01????0?000?0????0?000??0?0??1
00??01?0000001?11?000??11010000????1?1??0?0???11?0?10000???1?????????0??1100100000?
?????0????10?110?0?????00000??????????????11???????1100???????????????10???????????1?0110
1?00111?0101?10000010?010?0?00??01000?????????????00?????????00?????0???01???0?0?????
?0??

Coelurus ???
??100??0????????????????????
1??????????????0?0??????????011000 02001??0?0011110000?0?1??1???0?0021?????????0?????????
00001?0010311??000011?0[12]1??????0?????10??202??????0??????????????????????????0?0???0?00
01000?0??0??0???????????0????????????????12311200110???????????01[12]10120?000?111?0??10
?101??12??0?2???0?????10?1??0???????????????????????????01?????0?????????0??????1??????0?10?
?0????1??011?0????1??10???0???????0?????????????????0????00????0??0???1???????????0????1???
???????0???00?1????????????0???0??0??1110?????1?0????0???0???0????????00?00???11????????
??????01??????????01?0?000?????????????????????????????0???0?0?????????10?0????00?0??0?????????
?????0??????????0??0??0???0????????0???0?????1?????00??0??0???????????????01???0?0?????????
0????0????01???1?????1?0???010????001??100000????0?????????????????0????0?????00?????????
?????????????????????0?????????????????????????0?0????0?????????????????

Compsognathus
0010?002?0?0?2??100?1000110?11[12]0010100??00?0??0??10100011?11???0????00??11??0???
?0000?0????????0??0?00?0??????0??10????????????????????010000000[12]0?00000000??????000?
?1?0001???0?0?0??1??????10?00?0?10001???10??00??1??01???0?0002010?000?00??10?1?0?00
??2??0??3????2?0011???0?012100??110?0?010210?1112?0??2001?????0????????[01]0111?0?01
?0?0?0?00?0?1??????1????2?????????11??0?23?[01]??0?1011100000100???????????????????00
11???12?001100?0??0011?011??201110000110?0???0??1?????1??0??00?01?010?10?11??0???00
?0?0?0???????0???0??????1??110???0??0001????0?0[34]000?0?210????????1???10?1?0??2001?20
??????????0??[01]??0??01??0?0???????1?010?01?????0?0011???1????000?00?100?1?000000001
10000?0?0????000?0?0?00?10??0?0?1?0001??0???????0001???000?000????01?1?0???000??????
001????0?0?????00?0?000?0110????0????0?0?0?10???????00112?1???0100?1???0?0000000???0?
?01???010???????1?0?00000?0?0????????1????0??0?0?0?0001?0???000??100000??????000?110?
???00??0?00???0000?000??0?00???0?000???0???1000?00?0?0?0?0000??110??0020?00?0??0??0
000

Concornis ???

???
???00??0??0???????0201?1????11012?1202101
1011101011110?0101??21???3?????2?011[02]10000??1[01]0?????20?1111211?????1??????????
??20??01?1[12]??1?010????1?0????????????1???
2???????1111???0?2?111?11?1??101?01??11????????????1????????????????0?0??0?12?????????1?
???????????11????1????0??????0??20??????0??3100???????????0?????????1??112??1?0?00?1???
???????2??0?021??0??1???????10??????????0????1??????0???0????00?????0???0???11?????????
?????1??????????????????????????????????????1??0?00??01????????0???0?????01??????0?1????
??????0?1???11?1????????1?01111?????1?????1???01???????0????????1???????0??????11????????
????????????????????????????????1??????????????11111111????????????????????1???????????1
0?000??11100????????1??0???????12?01??????????????0?1????0???????
Confuciusornis
001002010110???01??0110111011??11010012000?11?1110?0002?011110001?000?1???101000
00002000?1?0?1110?????1??????????????????????????????0001?12????????1110010?1?1300??0?1
0010??00?0?0?????????0???10?1????0??0?11?110??0111101?0?00120110111110002??2?1001102
00010111?10101010021?112010?0210110121100?1110000?20011110005?2?0??0412?30?????0?
0?0110??11?0?0?000??120??1?12011??0?00?0220?2010221?10010[12]??011???11???0?1???111
?0????0111[01]11?0??1111?01100210110100110110110100?1?01?00?000?0?00?01?0?10011???
0??1?010????00??11010?1??21000??0?00001???0?0??10000?0?2?????0?00??00?0?1?01?2011?0
?00?1??????????0?001?0200?00?0?00????10?0?2???101012?1??00??00??001??001??0011000???
11????00????110111?0???11??0?111???0100101???????0?????100000?0011??10???0?0?0???1?0
1?00???001???000?0100000010100 1????011100011?1???00??2?1???0100?1?0?00111100 11?011
?101?1?0??1?????????0???001????0?????????????2?010????1101121??1???1120000?0???0?0?100?
???????0011????0?111100?0?00??110001????1?0??0?10????11101?1???1??0??020?1110?00???0
0?
Deinonychus
000000010001??001??1100000??10200001?001001?0?10110?00001????1[01]0????2??0?????10
?10101????????????10020?0001?1????1?1????1?02?1??01???0001000001000??000???111?011?
01?001?0??010011111201112001121010101 0?11101200001 1??11??10122110010011100100 1000
001010001021100000011?0211011010000111000[12]12101111111?2112???????4??????0212420
00101102?0000210100000?1000120211210012110??1?1210??0112000101102220111?0?00000?
0?1101100112 20[01]01001101110110000011011010120 10????????0?0100100110 1??000010?02?
?1?001010200?01000?10010?001001?0200000000010000201 0?1000000?10211?000?00?00100?
101??20010000000010?001?01000?02?00?00?100?0?010000?0?1001000?1?0???0000??0?0011
001?011?00?0?1110?0100?10010110??00210??0?0010000?0000?????100?????10?000???001111
0001010001011000?0100?1?1000000?0?000?01010001010?00000?1???0?1100112?10000110 11?
00020100??00010021101?0?0??000010000?00?01?0?000????0???0011010?0??110?1211111010
000?0??????000?1000?0?1???0?00???0?00??00??0000?????000??102??0000100?000?000100?0
11?0??1100000??00?01?0?
Dilong
0001000100?2?020?20110 1100??10200001201010200000100111101 1111?10101020001?000102
?000120?0?1?1?00010?01??01?100??20?10?????2?????10??0000000000000?000000???210101?
110?0?1??11?0????1???00?10?00222??00??0111?00??00?10????????0[01]10000000?0000???1?0
00?0?2??0?0310????????????0???10?0??0?01001?0210112201?2?2001???????????????0?2????0?

?0???0?0???01????201[12]?????????10?11?1??03?1??0??011100?0010022????0??0???1????????
1?1??????0?1001000001100 1?0?1????0?0??1?????????01?1?00?00?0001000?00?10?0?1?00?010
?0?0?000?1??0???011????100001?0???00?0?000?0?000?0???2?1??1?0?0???00?0??0??2001?[01]
00?1???????0?01?????00?00?0??000????1001?????10010111??10???10??00?000?0?00101?0000
1??000100?????010?0?00?11????0?????0?010?0??001??????10??000????????1001?0?0??????00
1?0?00?00100?01?????0????0??????0??0?0?0?1???????00??201???0?0??1???????????0????????
???010???0??1???11000????????????????0???10???0?????????????00?000???????11??00????0?
?00?0?????0000001????00??0??0001?1?1???0???001110???10000?11?0?001??00?10?0??0?00
Dromaeosaurus ?0?0???100?1101?10?1??0?00?0??2000111??????????0?10??00?1?11110011
012010??01?1?01???1200000000000010020?00010[01]1000100100000020??1?1001000100000000
000000001?11100110010001100 1???
???
???
???0???0?0?????00000?????????0?0????????0?
01?0?????????0???????????000??????0???????00???????????01???0???1??0?0????0????0????????
?00?0??????????00?00?0?0?1????0???????????0????????0?0?????????????0???????0?0???0?01?01??
??0?????00?101???0?1??????0010100000000??1?????????????1??000????????????????000?0????
??????0?0???0??????1????????????1?110?0??????????????0?????2????????????????????????????1?
?000?1?????????????????0????0?0??????????????0?00???????????0?1100?00????0000????00000
?00??????01??????0?1????00???00?0??????100????0???1????0????????10?0
Dubreuillosaurus ?01000010001?010000110001100012001012 1?[12]????0?????011100?11001?1
000020000000000000000??????????00?000??0000?100?0?01????0102?????1??100000000000001
000000?????0101??10???1???21????????????1??????????0????01110?00?0?00??1???????1?0?00??
????????????0??001??????[23]???00??
?00000010?1?0002?001??1?10??0??0??010
00???00????1?????????????????????????[12]??????????1???????????1???00?????00?000???????????
0???0???????000????????0?0????10??000???????01???0??0?0????0??0?10???01???0?1?10???0??
000???????????0??1???????????1?0???0?11???????0??????????21???0?11???????1????????0??1??0???
??0?0?0110???0???????011??????1?0???011?000?1??000?0??00??????????????0?00????00????01???
???0????????10?1???????1?0???????1??0??????????????00??????????????1????????????????????????
0?????????0???????????????????101????0????0?0????????????????0?0???????????00?010?0??0??000
0????00000?0?????00????????????0????0???00????????0?0???????001??????????1?0??0
Epidendrosaurus ?????????0??11?0???0?0?
00101???0???????????0??1????12????????????????1??????????????????0?????????2????0[01]21?00??
?1??0????0?0?0???0???????????????????0????0?0?????????10??0????????0???1[12]00?0???11?0???
?1?10010000?0?01????0?0000?021?11100?0?010100110210?1000000?2001??????3?0?00??[12]
12230?????1?1???0?2??10?0000000?0?010??12?11??0?00?[01]????1011000???0?0???0?????1??
????????011?????????011?1?0?00011?0110?2001101?011?0????0??01?1??1??0?0???????110?01?0
1?0????0??0?00????????1????1???????00?2?00??000???0??0??0000?0?211???0???0?????01?????2
?01?0?01?????????0????000?02????0??????????10??????????0???????????????????????00????011?00?
1?10????????????0?1011?????????0???????0??????????0??000?0??0??000????1?????????0?0????????0
10?????0?1???????????0????????????????????????0?0??01???0?002?0???01?????0??????????0????????0?
??????????????0????0??????????????????????????0?0?????1???????????????00??????????????????????????0?
???????0??1????????00?????00??????????????????????0???1????????0??????????????0?0??0?

Epidexipteryx
01???10??0???????11????1??????300???001?0???0??????????????????0???0?1?1??010?????????
?????????????0??????????????????????????????001?00110?0??1?????10???0?????????0?????0
?????????????????0?????10???????????????????0?0?[01]200???0??1??????????????0??1??1???
?0?000??????????????????????????????????2??101????5?0?00??41??30?????0?0??01???00????
?0?0????0?0???201?0????0?0?10?0011000?1?1?0???????????????????[01]?2????????011???0??
??11?????????1??????11?0?010000????????????????01?0????????0???????????????????0?1????
??????????????01??0????0???0?????0??????0???????????????1?0?0??????????2???????0?02?0??0?
???0???????1?????????????????????????????0????????0?????1????0???????????????????????????
??????????????100????1??????0???????????0?0???????1??0????????????0?????????????????????
?????0100?????0002?0?????????0???0????????0???????0????????????????0??0??????????????1????
?????0????1?????????????0??????????????0??00?????????????????001??0?0??????????????????1???
????0?????????????1?????????01??0????????0????
Erlicosaurus
100001010101???11???000111101122100000100001110010010002001111001100001001010100
00000210??0001001001000001?01010000?1?1?2?211011?111001011000010110101101?101000
0111000200000?1???0010
2?0010?120??
???2??????????????????????????????000??0?
???0000?0?10??00001[01]000?0?????????????0100????010000?????0?00?0??????00?0010?0???
???0?0?21?????00?0?????0????00010??0??0?????0????0?1?0??0000???01???0??????????2?????
00??2?00?00??00?0???0?0?????????1120????0??10????1????1?00???01?0?00??0?1100?????????
?10?110???0?1???0?00010??110?000????????????0?0?00?0?????????????0?????000????10????0
00????1?00??0?????????????0???000?????????1????1??????????????????????????0????????????000?0
????????????1????0????0?0??????????????1?1????????????0?0??00?00????0000????00?00?01????
??????????00?0????01????0??????????00????0???02???0?0?????00?0
Falcarius ?????????????????????????1???10??[12]20??0?0?2????0????????????????????0???
0?01???01?????0?01???100?010??????00100011?0?100?0101??1?????????00011000021001??01
0???????0??????????????0?1??????????01101102??1?111111[12]11?[01]???0????10????0[01]2110
0000100002001?00001010001031200000001100[12]000110101101110002021011120102?2112??
???0?1?10?0?[12]01211??10?0?0??000110101?0?110?000001?2012111001[02]1?2110010111000
100?01201110?0?0??100?0110?010?11200010?00000001010010???11010?01??????????00011?1
??0?0??010?0?1?00?1??101?01000?000?00???0??0?1??1?02000?0??0?00000200?0??00?00?0?2
10?0?1??0?00?0??10???2?0111?0010?0????21?0?0?0?02????0?0????0??10??????10?0???1?1????
??0??0?1?1000????01??00?001100?1????????0?1000?10????11???????0??????0001?000??11?1000
00???00?0??0???000????01?0000????0?1?????0?0??0???101000001?0????0?1????????0????2?11?
?0?000????0????????0?????????1???1???1????0?1000?00011110011???1????01?010????1?????????
?1???0000??0????????????0011000???00????????????????001??1000??0?12???0000?????0?000?0?
?011?01???0000????0?1??0?
Gallimimus
1000100001000??01???10100010 11??000?1000000101000001000111111?00??10011000000010
000002001?000?00200100100010111010010101?02??0?00110??0??12?????????0010???1010000
10??00010000?1?010?11?011101002101111100110100010?010010000?01201000000010020010
00000000?1002000000 1010?100??102001001010010021011010001?2000??????4100??01?0121

0??00100110?002001010121100?12010020111101100 2112[01]00001210010010012010221100 0
01100011002?00123?00?200121100 11001100????1100?11?????????100 1101?0010001111100?10
00?0010?100?000000?11000??0010101110000???000000?100100 0101000??201?110?00?000010
??0??20210100000 0????0?110 1100000000?00?000?00010?0000110000000?0000?000000001100
?0?0010001100100???1001??0001010????0000000010?00?000?0?100?????01?10?000?0?0?10?1
0001000?0??00?020000??001???000?0?000??1010000?00???0?0?1??0011000112?1?110?00110?
00100000000 0?0??010?0111???0??1?0000010111?00010????1120???010?0?01????2????????100
000??0??00?0?100000?0?000010???00?00000??00000????0010?000??0?00000??00?001000?111
00??020001000?0000010
Gansus ??
???
?????????????0????????????1???????????????1?0???0???0??0????2?1?1201?111??10102?02?1011
101??0101??1101010?1021?11302???210?12101001?121?????20001012018?2?1??34??????????
?????11???1100000010????0????0001[01]00??21?0?2??2010201???0???????????2??11????????2?2?
2????????121111???1?011?0?00??110100?001??1???01???1????????0???????????0?00?0?1??0????????0
?1???????10??11?1?????1111?????0??20???0??????10?0??????????????????????????002?01?0?00?1??
???????0??00??20??1????????????10???2??????????????????????????????????0??0????11?????????
?10?????1?????????????????????????????????????1111101?1?0?????????0?1?1?????01??????0?1????
??????0??????1?00????????211???1???????0???2?1??????1??????????????1????????11??????0?????????
?????????????????????????????????1????????????????11?1?001?0???????????????????????1????????????1
1????????0????????????2??0????????11111????????????????0?1?????0?0????
Garudimimus
1000100001000??01??110?0001011??0000100000 0101?00001000010111100100001100 0000?01
00001201110?0100200100???0?0?1???0?1?1???????????1?0?001??12?????????001000?10 1100010
10000??000???0??0?100??10?10?1?111???0110?000?0?0?0?10??0?01?????????????????????????
???410?000??????00?0?????????
0??01010121100202000 020111101000 2112000[01]???????????012010211000?0 1100111001?0?1
2300011001111001100 1?0020101?0??110??????????0?1?01?001?00?101?????0??10?1????????0?0
01?11000??00101?1110??0???00?0????00?00?1?0000?0?1?001??00????0101????200???0???????
?0???1101?0?0??000?00?000?0?????0???1100?0000???0???00???0?1??00?0??00?0100??????10
????0?0??????????000?0?001???0?00010000????0???0????????0?0010?100010?00???0???1?1?01??
??????0????000???0?0??0????0?????????0??1??0??201????10??1????????????0????0???10?010????
????????00010???0?00?1???????????????0??????????????????????10???????????0?0??00????00?0001????
00000?00??????????????1??000??0????0?????????1000???1????02???1?0??????0??0
Gigantoraptor
1??
???0001??12????????1221?1011?020?????111?11?0??
???2201??1??????????????????10?01??0??21???010
00????11?1201???01?10?0????????????0???2101???????????????1[23]0120?1?00100000?01000????
???????????????????????????1????10?1?????????????022?0?11?011???0????2?0?10???23100?[01]00?[0
1]??1??????????02???????00?01????????????????????????0???????00?????1??10????[01]???0????0??0???
1????????21?0???0?0?????????????????0?0?01??0???0??????0????1??????1????????????????????????0?0?
?0????01???0??????1??????????????????????????????????10???00????1????0????1??????????????????????????
?????????01?????????0???????01???1?????????0?????0???0?01???????0??0?????????????????????????????

????????????0?????????0???2???????????????0???1???????
?????????????0011?1????????????????????????????????1????11?1111?111????????????00?????00?
??????????????????0?????????????0??0???????????????0?

Gobipteryx ?01002?10110???01??001011110?0??11?1001100???0?1???????????????????
??0?????????????0?011????00?1?12????????020????
????0???0??????????????????????1?????????????????????????????????????11111??11012112?
200?10???010????1?10?0??02???13????????????????0?100?????2????????17??????????????????
?????1????1??????????????????????????1???000?20????????????????????0????????????1?2?2?????2
3?111011?0001111?11111?1?0??????1??????????????????????????????1?1?????1??????????0???
?01??1?0?0??0???0?0????0?????????????1?1?0????????????????????1?01?2?01??????1?????????
?2??0???00??0?1?0????????0????????012?1???????????????1?????????0?0????????????????????
?0???11???011????0??0??????0????????10??00???00????0???0???0??????1?????????1????00????
??????1??????????1?????1???????0??????????????0????????????1????????????1???????????????????
????????????????????0??????????????????11?1?1?????1??????0??????????01????????????1???0???
?111??????????1??0????????1??01?1??????????02????????0???????

Graciliraptor ??
???0???02000???????????????
???00
??010?????010101?021?112010??0101001112?0?0011112?2111????????????????1?4?????0?1?2?
0?0??1??0???0??23??[0
1]???1?[12]??1111?0??0?????00111??0?????????0???????????0????0?0?????2?1??2?????[01]?1????
?????0?0?0????1?????0?0???????00?2????1?00??0?????1???0?????0??????????2??1?0?00?????0??
?????0?0??????????????????10??????????0????????????????0???0?????01??00???11???0?????1?1?
111??00??????0?????1????????????1???????????000???0?????????1010????????0??????????????????
??????????0?1??????0?0???001??12?????????????????1?????
??????????????1????0???0
0?????11????????????????????????1??????????0????????????????????

Guanlong
0001?00100020020???1101200??10200101201021210000110111110??111?1010002000??010012
0000020?0?0????0110100001?100??20?10?????2?????10??0000000?00000?00???00?12101???
??00000??00?0????????00?100002?0??10??0100?00???0?10?1??0???0211??00??00??1??1???00
002??01?3????0?0011?02010111010??0101000202101111101?122001??????30?00????0?2100?011
?0??0??0???10111011210120000?21001111110311??0?1010100??010012?11201?00??1????11001
11?12???011???0?0??11?0110?2[01]?10?00011?????????0??1?00??0?00010000?0?10?1?1000?0
1?1??0?00??1??00?0011????[12]??002100??00101?001??00000?0?[01]1??0101?0????0?01?0???
000?0000?????????0??0????100?10?0???00??1?1000??1??00?10201???1?1?101?001?00?010?101
?0?1011??00100?????0?01??00?10??0?1?????11010?00?001??0????0??000???00????01??0?0???
????0100??0?0??00?01?????0????????????0????0?0?1????????00??2?1????10??1?????????????0????
???1????00???????1?0?000001??0??????????0?1?00???0???00??????????????0000????????011??00?
???0??0??0?????000?000????001????00000??10?10???010010???100010?1?0?002??00?10?0?0?
?0?

Haplocheirus
000?1??100?[01]1200100110111?1100022010?2011001000?00001???010111??0101000???????1?
000?0?10??0?????0?1010???11?1?????102????????2???10?10?010?000[01]01010001?0000?001?1

00??100000??00?????????????1??10[12]1???0???1100?010???1?0?11?0?0?12110000??000?1??1
?0??00?2??0??3?10?110001??11??1111???1101000[12]021?11[01]201?1?2001??????30?00????0
[12]1???????0?0?0??01?0??1????1?0002?0??10??1??0??02?[01]?[01]?000101000011012?002??
?00??1????110?2???1?[23]?00?1?0?0??0??1?0?1??2?????????1?????????1????01?00?100?0010?
0?1??1?110?1?11??0010?0?1?????00???1?010?100?0???00000???0?000000?0?21????0??0??020
???????20?1??010???0????2110?0?0??0??0?00???0????100????????0??0??????????0??00??00?0
??11?020??11000101?0?????010?0?1??1???????1????0??0???000?000???00??000???1??01?1???
0???????0?11?1??0?????????1?1??1???1??1????11??0?????1???1????1????1???0????1??0?0?????
?0???????????110???????????0???0??1??100?0?1?10???0??10????0??????????????00??????????0
??0?????0????????????????????0????00??????????????????????????????????0???????0??021100???????
??0?
Harpymimus ?000100001000??0???110?000??1???0000[12]000000101?00101000010?1?
??01000???0000?0?000000[01]20???????00?00???????????????????????????????????100111020?2?
??0000?000??010000???000010?10?1?0??1?????110?0020??011000?20??00??0?10????0???1211
00000?0?00200?0?000000?0100?10000010100100?0110100?010100110210?1000001?2000????
??4??0??11[01]0121????0100010?00100[12]0?0?0110??0?0???20??11??1?0????0???01?[01]???0
????120?????????????????10???0?12??00??00111100110011 00????100001?0?????????10?1??100?
?0?0??1?1????010??01????1?0???0???100???001010?1?0?000?0???000?100??0?10?00????11???
0??0??0?01??00??2001010020???0???1??0?010??0?00?0???0??0??10?0??????001000????????0?
??00??00????0100001????0??0??????0?01010????00????0?0000??000???????000????100000???
??????0??10??0???00?01??????????????0????000??????????0?????0?0????00???00??2?????0?0??1
????000000000??????1???????????????0?0??0?????0001??????????0???10????0??????????????100??
??????????0??00????00?0?0??????????0??00??0?00?????00???0?00?00?????????????1?00?????????0
20????????0??0?1?
Huaxiagnathus
0001000100?1??00?20?10?[12]00??1?20110110100000?0????010001?1111??0?????0?0????????
??00?00?00?010???0?0????????0????????
???????0?0???????1??10?00?0??000?????????0???1??01??10?00021100001?000??0?1?0000002??0
??3????[01]?002?0020?01101???111000020210??12[01]1?1?2011?????0????????[01]01000???0?
0?????0?10?100?02?101?020010210?11?11?02?01?0?1011100?00100?????????????????1?001?
???120?011?0?0?00011?011002?11100?011??????????10???0??00?00????0010?10?11?000??0??0
?0?000????????0?100????00000???0000001?0??0?00000?0?210???????0??0?1??1?0??2?01?0000?
???????0????0?0?00?1??0???0?????10001??????0002????????????0??0?0?00?0???1?00001?000??
0????????001010?00?10????1???????10????????0????????000000????0????0???000???????0010???0
?0?0????000???0?0??0????0????0???0?10??????00??2?1???0?0??1???0?0000000?????????????0??
?0????1???0??0???????0???????1????00???0????0??????????????00?0????????????0??00????0????0??
??????0??0???0?00??00?00??????1???0???????????0??????0????0????02??0????????0??0?
Incisivosaurus ?1100111001112101120 10?1101011200001001000 1000?0110010021[01]011?0
000002??111?1010000001 2111???0?00?00??01101?2????10??1??????01011111?000111000212?
001211100??010000001?0?1110??
???
???
???0??????0000?0??????????0?1??????????1
00??????????0?0????????0000???????1????0?10?????????????1????0??????0?00???0????0??????????

?1?0?????????00?0???00?0????00????????0110???00???0????0?????0?????0??1?0???0?11??????
?0?????10?1?0???0?????0?001???1?1??000??????????????0??1????????????0??????0????????0
????0?1???1??????0??0?????????0?????0?????????????0??0?0??????????????????????????????0
00?0?????????????1????0????011?????????????????00??????????0?0??00?0?????0000?00??001??0
1??????10?????00?1?????1???00?0??????00?????0??001???1?0??????0?0
Jianchangornis
001?????????????????????????????????????0????????????0????????????????????0?????????????
???010?1000?212?0?0??????????????????????0?0
???????????????????10???????????????0??????0??201?111??1?1???02?10?11?2??01011???0?0
10?2021??120????010100101101?111?0???2?0012110 17???1?1?????????????????1????????????
???????????????????0?02?0??????????????????????????0??????????2?[12]???????1210?????????1
1????0????????0?0?1?1?????????????1?????0??????0??0?0?10?10???0??0?1????????1??11????1???
??0?2???????00???????????100?0????0???????????0??????02?01?0?02?????????0????000??0???11??
???????10??????????0??????????????????????0???????1???????11???0???????????0?????????????????
??????????????0?1?????1???100??????????0???0??????????01???????????????????????0????1???????????
??0??11??????0?????1???0?????????????????????????0??
????0???1???1?11??????10????????????????
??????????????????????????1????????????
Jinfengopteryx
0100???1?000?2?0?00010???1??1?2000??0010??0?0???1?0?????0??1???0?1?0???0010?0????00
0?20???0?????????????????[01]??????????????????????????0101?001021200????0????????1?1????
?????00????????????????????????0?????1???????1??01??11?0?[01]21110111?1?0????1?1?0000?0
?0??2????0101010021?01[12]01????1010012?210?111?[01]?1?2?1???????????????31233????1?
110?000?2100??????????????????1??1?????0????10?20??????????????????????????????012?????
??????1???[01]????11???0???????????????1??10??200??????????000???101?????1??0????0????01
00????????????0???????????????0000????????00???????10?????????????????0??2??1??0 00???????
?0??0?0????0??0?????????????1??0????????1??????????????????????1???1?0?0??011???0????????
??101????1?????0?1?????0??????????000?????0?0?00??????01?????01?????????01????0?????????0?
??????0??1?????????????????0?1????00??2?1???0?0??????????1????0??????????????????????????
??????????????????????????0????1?????????????0??0???????????????????????????????????1?1?????0
?00??0??00??1???????????????????1?1??????1??02??11????????????
Juravenator
0000000100?1?100?00010?000??00200100[12]00100010000??01100[01]11?11?0010?0?0?00???
0???00000??0???????0?00??????????????????????????????????000?000001010?000?????????000?
??????1??????????????????????00????000????1????????1??0???10?00?211??00??0????????0?00?0
1??1??3????0?0011???0??1[12]01???110?00?20210???[12]01?2?2001?????03[01]?0????00001??
????0?0?0?001??1?1??00?00??20????0[01]?11?11?????[12]?????????????????0????11????????1???
???001??????2?0011???000??11?0??1?2011??0?111?0????????0????01?0??000???001???0?11?0??
??1??0?0?000????????0?10????10?10???????000??0????000?0?0???0???????0????1?01?0??2001?
2000?????????0????00???0?00?0???0?0??01000?0?????01101???1?????0???1??00?01?01?0?0?1?
?0?0?0?????????00?0???00?00????1?????0100????????0??1????0??00?????0??????????000??????001
0?????0?????10001??000??0??????????0???0??????????00??2?1???010??0???0????????0??????????
??0???0???0?0?0?????????????????????????0??0?0????0?????????????000000??????0?0??10????????
??0?????000?000????00??00?000???0????00000????0????000??1??0?002??00??????0????

Khaan
11100110 11?20??11???11?210 1111??0000001000101000 110110020[01]111000000000?10101?1
00?00012011????0?000000?????????????????????????????????0101??12???????12 110100??0200?1
??111010100?1?0?1111?0?11??1020?10101????1??1??000?11??01?0012 110010?1100020?1?000
0?01?00003???00?00000021?011010??0101000[12]0210?1110002?2101?????04???0??1?????0??
??????????????0100000010??0001???01111??001???2??010111000100?0220?01?1?10??0????1?0
11??0????001000?01?0011?0120?20 1100000010????????0?01?11???1000???0000?000110101??
0??0?0?00??0?????0110?1??20000??1000000?1?0000?10000?0?21????0??0??0?0001?0??2001?
0000????????????0?000?00?00?0???01???010?0???????11010???0??0?0100?0?100?10001100001
0010????0????0001000????10??0?0?0???0?011???1?????0????100001?0?0010?10???0????????
000???0?011?1?000???1010?00?????????0?0?0?1??01???00??2?1???0100?0??110???????0??????
11???100010???1???0?????11??????????????1?????1100?110?12 11111??1000?0???????0?0??01???
111101010100?0010000????00??0??00????0??0??00000???0???100???1??0??010?00?0??0?0???
?
Longipteryx
201??????0?0?200??0????2?????????????0???0???1?
??011?002?0????0??0??????????????????????????0?
???????????1???10????010???????????????11?????0??20111????1?0???12??0011011?0?011???0
?01011021?11[23]0????210102?01110?1102????20011112116????1??4?2??0???????????11??????
?????????????????2????????21?0?20?[12]?10120?100??????????????????????211???????111[12]1
??0?0?011?01?0??101??????1?1?????1???0????????0?????10??2?1?10?0????0????1?0????0????
???0?1????00?2???????10????????12100????????????????????1??110??1?0?00????????1????0?0?
?20???0?1???????1??????????????2??????????????????00?????0?0?0???11???????????????1??0????
1????1????????????????????00?????1?100?0??01?????????0??????????0?????????1????0??0?????????
???????????????1??????11??????????????1???????????1???????0?????????1??????????????????????
????????0????0????1?????????????111111?11?00?????00??????????????????????????110???????11100
???????1??????????????01????????????02??1????????????
Mahakala ??00
0?????0???????????????1???????????001?0121?????????01?????????????????1???1?2????????????????
???????????????????????????1?1???????????????????????????????????????0?????????????2?1????????????????????
?????????[34]???1010?0?102??0???????????????????????1??????1?[12]??????4?00??1??1132???1
0???0?0?0?1?010000?1??0?020101?10?11100???????????????????????11?0011????????0????1?112
?0??23??01201?0?0101??????0??????1?1[12]??0???????????0?????????????0????????2????????????
??00???????????0?1??????201??????0??0??????0?0???????0?0??10?01??00????????1??????????????????
????????????????11???????????0???0100????????????
1?????????????????????1?1????????????????????1?????????0??1???0???00????????1?????????????
????????????1???????????????????0??????????2?????????0???0??????????000100??011???????????1???
????0??????00??????1???0???0???????????????????
?????01????????????????????????????1?1????????0????????????????0??????
Mei
1?11100100?1?200?0???0?111??1021110??01?000?11?0110000?01111110011?000?0010101??00
???2001????10120100??????[01]??????????????????????????????01[02]?00010212?0?1000????11?11?
????00?1???0???0????2?01?10?10?1??010??1?10??????11??11110?0012111?1???110020?1?10?0
??0??0??31???0?010??021???[12]01???01??00?2??????????????20????????03?000???[12]12320??

11?1?0?000021?0?0?01??????????1?1?0??1???0??[01]??0?10?0211?1001??2011???1?0?00???2
?0?2?0??231?0110?11????11?21?012??11??11?1??????????01??1???????0?0?101???2??10??1??
0??1?010?????????????0?1??200?02???0???00????01?0?000?1??????????0???????1????2?01??0?
???????????????1???1?00?0???0?????????0????????0102??????????0????0?1??1???????0001?0110??
0???????????01?1??10??0?1?????01?011??????00??1??10???????010?0??????000?????1?02?1???
?001????0?????1?0??1?0??1????????????0?01????0?????????0????1?100?010000000100201010 0?
??1??1?????0????????0?00?????1??????????0????1?????????????0????????????????00????0???????
????1?01?0???00????010??0????210??0?10????0???1011??1????002??11?0????0???0
Microraptor
000000010000?2?0?00?101001??1???100?001?00??0??0??01?0?1??0??????????????????????????
?????????1??002?000[01]0[12]1100??001???????01?????
???????0??????????????10?10[12]???01??0011???0???01?11?1???001[12]0110111112??2??[12]?1
001100000102???00101000021?01201???010100111210?1112110?21?1101000[34]???001?3124
30?????1?2?00002100000001000012011 1?001?2110?2[01]?021001010211?1001?0[12]01110??1
???00???1?01[12]?0??231101000111000110211010011[01][01]111010111101110?11?0???0?0???
??1011?02?11?001??01?1?0?0?1??00???0010?1??20000[02]??0101000?01??1?00000?1?2110??0
?0??00?0??1????1011?0?00????????0??0?110?02?000????0??0?010?0???????101?????????0????
?00000?1??0110?0?1?1110?0?????1011111???0?10?????0???????0????????000??1??1?000000001
????0??1010?0?????0200????0?1????00????0?00????0?1???????0?0?111?0???00???2?1???010??1?
0?0?0100110?0100201000 0?0??0???1?1?0??0??1???00?????1????0??010????1??1?????????0?00
?0??????1?0???0???100?????1????000?00?0?0100?110?110??0?2?0??0?1?????0?001?0???1??0?
??20?1????00?0??0?
Microvenator ???
???1011?12????????02?10?0?????
??????????????0???0111?1?01?10?102?01????00?2111000?00???11????0??????????1000200100
0001010000021100 0?000?????1?11????????0????????????????????10[12]??????????0???1?0?????0
00?0?00???00?010000?1??000201??2??1???1?001?[12]210?10???????????12?0101000100000?00
11??[12]?0??23???1??0?0???????01??????0??????????0??
0?????0???????0?0?0?1?11?0200???????????0?1?0??????????????10?????0??0???0??1?????????0??
???????????0???0????????0???????0?0?0??????00???????1?0????0??????0??????????1?0??0?11????
??????????????????????00?0?00????????????????01????0???????0?1???0??10?01???00????0?????
?1????????0???????0?0?001??0???0????????1???0????????0???001?????????????0000???010??1?0???
???1?0??????????0000?0????00?????1?0????110???1??1?????0??0????????????????111???010???
????????????00??????????????2??????0?????0??0????????0????0?????????????0??0?
Monolophosaurus
00000001000110?0000?1001110000200100201221200000 10110111011011111000 20?100020101
0011000100010?00200001001112????10?10???0????1?????100[02]0000000000000000001???100
110?1000010?01?0100111110001 1?1?2001??0100111001000 0110010??0?0[12]????????????????
???30000????????0110?
1?0?????0??0111110110020210 0?1000110010 01?0000100101000?0?0????????????????????????
???0???00?00??010000??0?1?0??1?00????????
0000?1??????0100??0?????0?000?0??0??000??0?????????0???11?0?1????000?00???0????0??1????
???0??1????00??00?0???10???0??011????00?1121???0001?0????110??0?10??01??000???1?0101
?????0?????00?100???1?0???010100?100?0000???0?????????????10000??????????0??1?0101?0

????00????1?0???0??????1????????1???0????0112??1????1011????0?????????????????00????0
??1???000?0???0?0?0??00???1?0????0?????????????????1000?????????0?0??10???100?0000????
?0100?00????????????????100??00????0?????????0000??0??001???0?0???1?0??0
Mononykus ??
?????????????????????????????????????1[01]??0??????????????????????????1????20???????????
??????????????????2????????[01]?01?[01]01102?11010001100?001000100001?0??1[01]2110000
?010000001000001120001141000210002??020?0?010011111?110????1?????????????10000??2?0
???????????0000?10?0??01?0?010000???000020?2?????????1????2???21??????0???0220001000 1
10?00110212?20001130101200121100110011 00?01111000110??????????111?????11??????0????
0?01?1???10?10?01?????0??100????1??2111?????0??01?0????0????1110??1??????0??0???0?1??
??2?21?0?1??00100???1?110?00??0??1????????????0???0010100????00?????????0????0?????0?1
?21???00?????????100??00???1????012?2???????????????????????00??1?0??00?0?????01???0??
10?1??1????0?1?????0?00?????1?100011?11?00000010?0???0???????000010???0??0?010?00000
000?000?0???????????????0?1????10?001???1??2????01?????000???10?0?????0000??0?????????
?1?10000????1????????????110??11????????????????000?????0[34]101????0?????????11??0???0?
1??1?
Neuquenraptor ??
???
???
???
???1?????1?????????????10??????23??01?00?11
1101110??01?01101011??0??????????????????????????????????2??1????????1?0????????????0?0????
??????????????????????1????00?1?????????????????????????2?0???????????10?????????1????????
???0??????????????1??????????????????????
??????????????????????????????????0????????0???????????1??????????????????????0??????????????
??0?????????????????????0?????????????????????????????0????????????0?1???1????????????????????
???
???????????1???????????????????????0??????
NGMC91
0?10?0??00?1?2?0?20?10?010??10??00???00?00????????0???0[01]1???????0???0???0??????????0
001??012?0001010100?10???????????1????????
??0?0???1???????????01??11???1?????2???01?00??01?2?
??00?01010021?1120??0?010100111210?10121?0?2111?????0?????????[23]??4????????1?2??00?2
???[12]?0?2?????????010?
0?1??0?11??1???00?10011[12]01?11?10??10?????1????010???10??????11?00???????1?0?0??????
??????0????????00?????????000??????1?000?0?1???1???0????????0??????????11?0?00?????????0????1
1???2????0??????????1??0????????1??????????????????????00?????1?00????11???0?????????????11??0
??1?????0?????0?0??????????000?????1?0000???01????0???0?0????????02????????0???????0?????0??
?????????????????0??????????00??????????0?????????????????0???????????????????????????????????
????????????0????0????1?????????????0???100???0?
11???????????????????????????1????????????1?????????????0??0?
Nothronychusgraffami ??
???
????????????????0?1??????????????100?????00?11?1??0?01?00?0??????????221100????10??1??1?0

?0010200010[23]12?00?001???????10010??00110002021 0?101?0???2?02?????04?01001?[23]0[
01]???????00000?110?0??????2????01?2?20?2?0????011?22?0?2110001?110?0222001???????0
000?1???0???11[23]?0000?0?00000 11000?002000010??11?????????0?????????0?0??????100?00?
0??0?1????00?0?????????0??0????2??20?0?????????00?20???????000?0?200???????111?0??0????
1??1?1?1???????????0??1000?0???????????????110??????001?????????????1???1????00?????1??10
???001???????????0??0?0?????????1???????????????????????????1???0???1?0?????????0?????????0
0???????????????????0???????????????????0?1????????????0?1???110???0??????????0???????1??
???????????0??????????1???0201????????????????????????????????????0????????????????????1?????
?????????????????00???1000????1?????????????????0??????0???????????????0????0?
Nqwebasaurus ???????????????????01?1??010103?00?1?0?????????????0?????1??????0???0
11?001?00??????0?????????1????????????1?0?0??????????1?????????????????1??1020??????????
??????????????????0?????????1??1?10?10[12]1?10?01?11?0?0????1??????????0??211000000[01]?
0000?11000000[12]??0?03????0?00100010??100110110100?1[12]0[12]?0?0020[01]???2000????
???0[01]?[01]?1???????????????022
00010???0???1???1?011?1?123?0010001010001101110??0?1100??11?????????11??1?1??0110??
0??0????0?11?1??0?10?0?010?????0???0????????0?1?2??????00120????00?00?0????0??0?00??
0??0?1????2?21?1?[01]????????????0?000??0???????????????00????????1?0????????1?????0??0??
?0??1??01??00???00????????????0001??1???????0?2?????????0?????????????????000000????01?1?
???10??????????01??????0?1???????????0????0??????????0?0?1??????????0?11??1?110??0????10??
?????0???????1???01???????????????0??1??????????1????????0?????0???????????????00????????
?????0?????????????????????????????0[01]?????001?????????????0????0???1111??1??01??200???
???0000?01
Ornitholestes
1000010100?1?210?111101210?0102001011010?00?00?001011?0[01]12111100000?0??0100?01
0??0000200010???002?000??001?1?0??10???0?1?????????1???000000100[12]0?000000001?1113
00???11?001??10?????????????1120001?101????01100?00000001??11????01?1?????????????????
?0000100010311??????1?????????01?00010???1????????????????000??????3100???0?0021???00
1010?0?0011010101001010 12010?2100110 1100??020001010100100100?????10???0?0?0??????
??????????01[01]?0?0000010101?0?????1?0??1?0????????0?01001?0???00?0000?0?10???1100?
1?0???01000?10?1???010?1?0???000000000?0111100??0??00?0?11100???00??00?00??0??200?0
?00?0????????0??1?000000?00?01??0????0?00?10???000?0111?00???0?000010000?1??0?000001
01000?0??0???0?0????????[12]00??0?0?11?0000000000??0000???1???0???0????10?0?0?000????
1000100??0?0?????10?10?000???0?000?010???0???1???0???00??211?????00?00???00000000??
?00001???00????????1?000000010?010000????????00????0????001???0100??1000??????????00?1?
00???00000??00????0000?00??0????????????0?10011??0???0??0?????0?00???????001???0????0?
00???
Oviraptorphiloceratops ?1?????[01]1????????????11???01111??0???00??2?2????????01?00102111?
?000??????11????????0????0??????????????????????????????????1???1????????0?1010???12????????12
11010???0?00?1???11?1??211?????????
????????????1???0?????????0?00??21??1201???0101?0120?10?11100?1?2102??????????????????
??
??0????1?????00?????0??????1?00?????????????
???????????????????0???2????00????????10????????????????0???0???????????01?0?00?????????
????0?0??????0??1?0?0????10?0???????1??10???????????????0??0??00?01100?0??11????????????0

?00??????????????0???0?????????1????0??1?0??00??????????????0???????1?0??0??0????????0???
?1?1??????????????0???0??1???????0?????????????0??1100100000000???010?????11???????????
????????????????????????????00??????????????1?????????????????1???11??0001?100?001???1??
0?00??0100001?????0?????????????????????????????01??????????????0?

ParvicursorinaeindetIGN1009 ??????????????????????????10??????????????????????????????????
???0???????????????0
0?????????0????????????2??????????????0?1?0??????????11?????????1???0???????2????????????
??????????????????????????????02???101?011?11?11????11?????0???20?0???????2??????11?2??
??00?010?000?1???????????????0??????01???0???0?0?????1?????????????????????1??0????2?2?
??0??13?001??0?2?10011???????01?00100110??????????0?1?????01???????????2??1?1???????0
?????????0?1?0?????????1?1?????????0??0????0????01?0???0???????????????1????2?01????????2
??????????1????????0????????????????????0???????????????????0??????1???1????????????????
????????????????1?????0??????????????????????????????0????????1????????????2?1?????????????
????0???????????0?11??0?????????????????????0??00????????????????????????1???????????????????
??0?????????????1?2?????1???????????2??0?????????????????
?1??????????????????????????????1????????????0??1????????????1?

Patagonykus ???
???
??????????????0?????????????????????1?????0???0?001???11??11??1?????????????100000?11?000
11?00?11[34]11002100[01]??020?0??1100011????0???????????????00???????[34]20000?0?????
??00?001?????0??0?0100????1000?11?1??????0?02211220?10???0?0?????[12]?0?0201010?000?
??1?0?[12]?00123?10??0??010??????????????????????1????????1??11????0?1??????0??????????
??????1??0????1?0000?0????1??200??????0?????00??????????00?1?0?1?????????????????????0??
??????01?????????10?0???0????????????1??0???0???100????????????????00??????????0?1?20?????
???????????????0??????????10?2??0???????????????????????0?????1?0?????????1??0?0???0?0??1??
????1????????0??????1?1?0111?11??0?0??????????0????????1???????????????0?000000????00?01?0
???????????????????01?01??????2?????1?????0??????????????00????????????????????000????1?
???????????????10????????????01????111?????0?00?????010??????11??????????1??1?

Patagopteryx ??0????????????1?
00???0?????????2??1?1???11???????????????????0??????????????????????????????????????0?????????1?
????0??0?0????0??????????????0[01]0?10[12]?????0?01?0???01??01?0?0??1???2211111111110002
?02??000?010?010311?10?00????2??????0??0??????1??[01]01?1??01?????????111???7100???????
????01???00?0??0??1100000110?00002????00???0?000?0221?2011[01]1101??10?200010??11??
?0?1?1?112????????12100110?0?011?0?10??0?1??010?10???????????1??????0?????????0????0??0?
???????10?????????10???1???????1211???????0???0????????????110????????????0??????????????2?????
?????0??????????21??000?0??????0??????????????????1??????01??????????0???????????????????0?1????
?????????1????????????????????????1??????????????????????11?01???100???????????????????????1?0???
????????????0??0??????0?00???????????1?1???????0???2?1???
??????????????????????????????1???????????????????????????????????????01?????1??1???????????????????????1????
???????0?????????????????????????????1?????????1?1???????????????0??????????????

Pelecanimimus ?0?1?00000???210?0001?1000??1?32000?2000000????00010011101????0?
??011?0??????????0?0?20??????????????????????[01]?1??????????????????????????????010110110212?0??
0?00???01?0?????????0????0?0?0??0?1?1??10?00?0?1?101?0?1???00???????1??????????????????0???0
??????1???????????1????0?00?10000100??10210??0001001?0211?1110002?200000000?????????

??
??00?????1??????0???0?0???1??????????01??????
????0????????0???010?????000??????00?1??1???00????????????????????0?01???0?????1?10?0???
?????2??0?1??????0??0??????????1??0???????0?00????????????????100?????1?0??1?0?0?0?0???
???????01??1??00????0???????000???????00????????00000?????0??0???0????????00???????????
???0????0?0????????????????0??10?0????111???????????1??001?????00???????????11????????
???0??????1?????????1?????????0????0??????????1?0??0??????????????0???1?0??????????????0?
??00???00?????001????????0??????????????0??????0?0020??????????0??1?
Pengornis
0010?[01]010000?200?0???0?111??20[23]0100?0?0[01]00?????0?101?0?1????1??0????????????
???????01???1??010?1?010212????0??????????????
?????????0???????????????1??????????????????0??????0??????0??201?111??1?0?2??1?2011102?
10101011?0?010?1021??????????????1???10????[01]0?????2????????1[56]???????41??3????????
???11????????????????????????????????1?0?20????????????????????????????????????1?[01]?1?????
???1111??????111?1?1???1?1??1?001???????????????????????????10??1?2?1???0??1???0?1?0???
???????????1???11??????????0?????????02????0???????????0????????1??1?????1??0???????????0???
???0??2????0??????????0????????????????????????????0?0??????????????????1???0??????????????
01??1??????????????00????????????????1??00??????????????0???0?????01?????????1?????1????0?
????0?1???????????????11?????00?????????0????1????????????1????????0????0????????????????????
?????????1????1?????????????????????????0011?11???1??????0????????????????????????????????????
111??????????1?????????????1??11?1????????002???1???0??0????
Protarchaeopteryx
1????0??0001??10?12?????????????0?????????0??
???0???0??00?110002???0?????????????0???????????????0?
????????????[01]???0[12]??1??0????1????0?????????????????1???00???????????1?0?00??1????02?
???000101?021?0110??0?01010012021??111[01]1?1?2??1?????0????????2?1?1??????0?0????01
?010?0?0010??020????10?11??????1?0?100?????00??0????2??????????????????????1??20???[12]??00
11???1???011?011??????1100?011?1??100?00????????0?0??????000???0?11?21??????0?0?????????
?????10??????0??000?????001??0????00?00?0??1????????????????????1????2?01?0?00?????????1??0?
000??0????0???0??????1?0??????????0???????????????????????00??????1??00???11?0?1???????????010
?1??1?????????????????????????????0?????????0?0?0??0????1???0??????????01????????????????????0?
??????????????????????10??????00??2??????0?????????????????????0???????1?????????0?????????????????
???????????????????????????1?0?12???1???0??0?????????????????????1???????????????????0???00??
0??00??????????1??????????????1?????????0?00??????????????????
Protopteryx
001??1??0010???0????1??11?????????????0???0???????????????0?????????0?????????????????????0[01
]2??0?0?010??0?0?102?0????0??????????????????????????
????????????????????0?????101????????????0??0????1?0??2011111??1?1?2?02?10011011?0101??
0?0?0101?021?1130????210100101210?1112????20011112015????1??412?30???????????11????1
?????000???????????2????0???1?0?20????02??????01?0???11????1????????0?[01]?[12]????????111[
01]???????111?111??21?1101?011?111?1010????????????0??????10??1?0?10?0????????1?1???????0
???1???0?1??211???????????00?????????12000?0????????????????????????1??102?01?0?00?????????1????
000??2???0???????????1???????????0?????????????????????????00??????1?000???11???????????????10??
?????????????????????????????????0???????11?00?0???1??????????0???0??????01?????????1??????0????0?1

???0?00?????????????11?????00??2?????0???????????1??1?11???1???0??????????????????????????
???????????????????????1???????????????1?1???101?00??11
1?????????1??????????11??1???????????0????1???????0????

Rahonavis ???
??
?????????????0????????????????????????????011?11000?0??1??11??1?01201?111?1?????????????
??????10[01]11??01010???40210??0?12[23]2???10
01?0?0?0021010100200000120101112012?10121?0210?10002210100110?0001?0?10??00?1011
112?01?22?10101010?01011?0100120111001101 0?1?????????1?????0?????????11?02???????1??
02?0?1????1?0010?0?1????021??????1000?????0??1???000?1?21?0????0????????1????2???????
?01???????????2?0?00??????????????0??????0???1??????????????0???0??0??????????1?00?1??10??
??????100????0????????00?0???1???????????????????1???????011???????1010?????0?0100????
0???????0?0??0???0??1101?????00???1?????????????2?1???01????10?0?1?1101?11011100101????
????1201????0?01??0000??????0?????0???????1???????????????2????????0???????????111??????1?
?????????????1????????????????1???????????????101??????1???????001?1??00??????

Sapeornis

0110?1010000?20??110???11?????301???0???000111????????0???????1??????0???1???10?????10
0??????????1?0?10???????????????????????????????????0011112????????010????????1??00?????0?10
??0?????????????0???10????010?????????0???0????????0?0?1201101???1?0020?1?1001000000100
11000101010021?1130?0??2101101[01]?111?0110????2001?????05??001??[34]12?3??0???0????
110??010000000000120[12]10?11012??0021?02200201022????01?[12]20?110???00???????1?0?1
???????111[01]01?0?00011?0110?2101????011???????1???0?1?????0?0???????0?01?0?10?01???0?
???1???????000????10?1??21?0?????0?001???0????02000?0??1?0???????????????1??0?2011?0?00?
1?????????0?002??200??0????????210?0?0?????0?01?????????0?????????00?????11000???11???0
???????1??101????11???????????????????????????1????100?00?0??0????0???0???0?????01???????
?1????00????0?????0?00????????1?0????01????0????1???01???011?0?1?111111??111????1???01
????2???????????????????????????????????0????1??????????????0?10100?00????0??00????????????1???
???1???????1?00??11000?????1?0?00?????????1?101??????1????00?0?11???00?0??0?

Saurornithoides ?00110010001???0???010?001101?21010?10000001?100??010??01??1???0???
???1??????????????????????????????????001????11???????1110????0?0????00??0001?1111???010??
?????1?0????????????????????????????????1??????????????????10???0?????????????????????????????
???41?0?????????????1?0??
?????0????????????????????????????????00???2???0??1?00?1000?22001????2???????????????????????
???1????2??0011?2??????0?1??011?1?????????????????0??????0??100??0?02??1???11????0??10?1?
???????0?0????21??00????????0?????1?0???0?1?2?????????????????????1?0???0????0????????????2??
??????0??00?0????????????????0??????????010????0?????0?????????11?????0?0???????0?000???0??0??
?0?1???1????0???????0001??????0???????????????????????0??1????????????????????????????1????????0??????
?0??????????????0??????1????????0??????????1???1??0???????????????????????????????1???1????????1?
1???????????1????????????????????????????????000??????????????0??00??0?011?????????1??0???????????
???????????????????????????????????????1000????????002?????????11?0????

Saurornitholesteslangstoni ??1??????????????
???1201???01???????????????????0??1????????0?????000???????
??0?????01?11?1[12]0??????????1???0???????????????
??112????????????????????????

???
??00?????????????????????????2?????????????1??
?????????????????????????????????0??????????????????0??0??????????????2???1?1???????????
??????????????????????????????001????????0????????????11????0????????1???0?????????????????
?????????????????0???????1??0
??????????????????1?0???
????1????0?????????????????????????0?????????????????0??????11????????????????????????00?????00
??
Schizooura
0112?2?101?0???0?????0?101??1???010?0010000?10?1?101?0?21010?1?0????????0????0???????
?20???0???010??12?????????00?0???????10???????????
???????????????????????????00?????????????????1??????????201??????1110?0?2?00?1??20?0??1???
???01010?21?1130????210110101101?111?????2000101?11[67]???01??4???3????????????11????
?????0??0????0???????????????0??????????1120????0?????????????????????????012?????????2111??0?
0?011?0?0???111??0?011????10???0?1??1???0?0?0???10??1?0?11?11???????0?1?0?????????0???0
??????10??????????21?????????13100?0???????????????????1??1?2121???00??????????????2?0?0??2?0
?10??????????10??????????????2???????????????????00??????0?0?0???11????????????????1??1???1????
?1??????????1?1????????1??1?10??100???1????0???0???????????10???????????????0??????1?????????
?????????????11??????00?????????01???1?????????????1???
???????????????1?????????????1????1??????????????00???????????????0??????????11??????110??????
???1????????????1?01?????????????02??11???????0????
Sciurumimus ?00??0?1?0??1??0?00?100?00??002??1?0?100?????0?00??1???0011??1????
0????????????01??0?110??0?????0210???0?0?000???000?01?0?0
11??1?00???1000?1???[12]???0??0?2????21?1??????0??0?11???0??10???????10?0??2100?001?0?
??0??01???0002?????3??????001??????01111???11?1????02?1?11101?1?2???????????2????????0?0
0[01]???0?0000?000?0???1??12110000200?0?00?11??000????0????10100??11?0???????[12]??0?
???????1?0?????????????1????0????11?0??0?2?11000?0?1010000?0?0????00????00????0?00?12?1
1?100??0????0??0?????????????01???10??0??0???????0??1????000?0????10??????????????10?1?0??2
001??0?????????????????0???0??00?0???????0????0?0????????11?0???1??????0???0??????11?011??0?
???0???0??1?????????0???????10???????????00?1?01???????1?0???01??????????????0?0??????0????????
?0??????????????????????01??????????????0??????1????????????????????????1?0??????????????????????????
?1???0???????????????????????????1???????????????????0???????????????0???????????????0?0??????????????????
????0??????0??????00??????0??????????????0??????????0?????0??????????????????????????????????????
Shanag ????????????????????01011?1101?2010?1[01]010??????????????????????????????
???010?00000201?0??000?
??????0?1??1??
??
??
??1????0??????????????0?0???????????0?0????????
??????????????0?????????????0????????????0??????????????????????????????????????01???????????????????????0?????????????
??0?0????????0????0????????????????2???????????????0??????????????1???????????????????????0???????????0??????00????
???0???????????0??????????????00?????0????????????????????1?1????????????????????????????0????????????????????????????????
?????0??1??00??????????????????????????????
1??0??????????????????????0???????0???????????????????????????????????

??????????????????111????????12????????????????
Shenzhousaurus
000010000100???0???010?0001011??000?2001000101?0??01000010?????0???01???000??2??00
0????0???????????????????????????????????????0????0101102??2???0?0??001?1??000????0???
10?????????????????????????????????0??01??00??0????1????????????????????????????????
????????0???????????0??????00??021??10[01]0002?2000??????3??0000??01[12]00???0?0?010?0
01?010101011000?201???01111?11021?1?00?10101??10?100???001???00??1?????????????????
??10?1?????0??00??0??00?1?????010?????????0??????1
???010???110??00??00000?11?0????10???0?101??????00??0??????0?????1?10?0?????????1?????
??00??00?0???0????0???0???????0000???????????0???1?00????010????1??????0?0??????0????0?
???00????0????00?00?????????000???????0?0??????????0?????00??????????0??????????????0????0
???1???????????????10????????0???2?1????????1?0????????????????????0????????????????????
??001????1???2????0?0??????????????????1????????????????????0????0??????????????????????00??
???00???????????????0???????????00???1?0??02??????????????0?
Shuvuuia
00011001??001??0?0??1001?110??32000?1000000101?0000000001011?000100000?001000100
0000110000101101001?1001101[01]?01?10020011?12?10?1???1010 11101020?0?00??000???1?1
001???00????02?????????[01]?01?[01]01102?0101000 1110?00100?100001?0???[01]?110000?010
000001000001120001141100210 02???????0???0??????????0?????????2??????????10000?520000???
?1220?00?0?10?0??010?1010000?0?000020020[01]111?00100?0220?2 11100000?0?0220001000
1?0?00110212?2000113010?200121100 11001?00??????????????1???????111?11010??0000?100??
?000?0????1011?10100?1100?1?0010?1??21?110??0000?1???0?0?10[01]0?11???1?00?0?00???1
?00??0??21????0??00?1???02?0??0?00010??10??00????0?0?0?????1??00100??00???0101?0?1??
10?00??02111000?0?0110??100?1?0??0??110??20010????000??000?0??0????00????01?0????0?
??010000?????12???000?0????00??0?100???01?0?1?11?0?0?0?0???0??000??2?1?????0??1?0000
0100000???00?????011??????????0?0??100????????????1??20???0?0????0??????????????00???????
???????001?1?0???????1??????00?1?110??11????001010?11?0??0?0???????0100?????0???0211?0
?0??0010011
Similicaudipteryx
11120?1100?1?210?1201 1?010??1?3000000?1?0?0?00????0?00?2111?11?010?0?0?111???1???0?
00???????????01?100???????????????????????????????????001??01??21??0?1?1010???0?00?????001
1???0?0?????????????1??10?1???10??1?11?10???00?11??00??0121?0?001??????0?1????0????????
31??????0?1?02?????2?????01???01????????2?0??1??1?1???0?031?????13??2?0?????0?0?011?0?0
1000?311000020100?11?11110?1????0001???100?10010[12]?0????????0??0???????0?1????2??001
2?0?1??0?11???1?0?011?000?11????????10????11????????????0??00?0??1??0???0??0????????????
????10????21???2?0????0?1??0??0?00??0?0??100????????????0??1????200???????????????1??0??
0??????0?0????0??????10????????0?1?????0???????????0?1?????????100?100110???????????????00?
??????????????????11?????????00?????0?????1??0??1?0???0???????10021???0?????????0????0??
??????0??????????????11?0?????0??2?1???0??1?0?000?????????????????0???00????????1???0?????1
??????????????1?????????1?????0???????????00????????????0????00????1??0?????????0?1???0????????
??0????????????????????????????1?????1????00??????????????????
Sinocalliopteryx
0000?0010001?0???20??01[12]00??10200100100?000100?01?1101?011??0??0???????0??0????2
00001??????????????????????[01]????0???????????????????0????10?0??011000???0???????11?0?1??

1?00?1????00?0???????????0?00????000????1???0???1??0???00?0?02110?001?000?20?1?0??0??
[12]??0??31???0?00010020?11101???110100120210?1220101?2001?????1????????00[01]110???
??0?00000?1?010?0?0111??02010??10?11????02?[01]??0?1011100??0110?????????????????????
0?1???????0011???0????11?0??0?[12]?11??0???1?100000000??1??1?00?0?0??0??10?10?11?010
??0????000?????00??0?10????????????????0?0??0????000?0?0?110???????????1001????2?01?1
000??????????????0????0?00?0??????????1?000??????010[12]0???1??1???????1100?????10??0?0?
00???0?????????0?010?00?10??0?0?1???0?000??????0??????1000000????0???11???0?0??????00
10???0??00???0???????0?0??0???????0????0???10????????0??2?1???010??1???0????????0?????????
???1???0???1??????????0????????????????0????0????0?????????????0??0?0????????1??00????0???
????????000?00?????00??0??00???????????0??0?????????1?0???10?0?002??0?????0?0??0?
Sinornithoides
10?????100???2???2011????11???21???0??0?0?0????01???0?001??????0???0?????????????0????
?????0????1??100?????????????0?????1??????????????0?0?00010111110??000????????1?????????????
?0???????????????10??0?1??0????1?11?????????01??10?0??[12]1?0010?1100?2001000??0000001
?210??0?0011?021??120???0101001202[01]0?10100?2?2012??????[34]????0??21232???10?1?
1?0?0021?0?0????????????1??????1??00?0[01]?0?100101020?010011???011???1???0??1?1?012?
0??23??011?01210001102100?2011110111111?????????0?1?????????00?????000?02?11?0?1??01?1
?0?0?1??00???0???0???2?00?0??????00?20?????00000?1???0???0????00?00?1????2?01?0?00??
???1??2??0?010?00?????????????0??10?0???????0??????????????????????100?0??01??00??0110??1?
?????00??000?????0?????0?0?1???0?????????000??????0?000?0??0?????????010???????02????1?0
?110?????????1?0????????1????0???0?10?????????????1???0???????00?0?000000010???10???????0
??????1????????1????0?????1?????1???0????1??????????????0??0?0???????????????????00??????1???
?????????????00?????00???????????????????0???1?????????0?????????????00?0??0?
Sinornithosmillennii
0010?00100?0?2?0?20110101???10200001200?000100?0?10100001??????0??0020?011010?0??
0001??0000??10??011??????????0?1??????????????1????????012000000[12]01001100001???1[01]1
1????00011??1???????????????????1???????????????????????????????????0?20110111111002002?100
1?00??0??2????0??1[01]1?0?1?11201?0?010100111210?1012110?20110010003?0?00??????????
?????2???????0000???1???0120[01]1?2?0?12110020?0210?1?1021101001????11??????????????
?????0???30001?0111110011?21001001100112000?????????00?1?01??0?01000010?1?01?11?001
??0??1?0?001?1??1??0??0???????00000??10?0?02?1?01?00000?1?21????0??0??0???01?0??1001
?0000?????????0????110002?0000???00????1?00????????01000???0?????0???0??00?0??11?0?0?1
1111??0??????1??1?10??00?10????001???0?000??10??1000????10?0000?0?1??100??101??0????0
01??100?001?????0??1?0??1?0????????????0?0?11?1????00??2?????01???1??00?0100100001002
1??00?0??1???01???0?00??1???00??????1????0????0?0??110?12???11??00?0?0????????00??000??
??????0??????100??000?0100??10?11?????2?0?00?100???00?01001??1??0?112???1?0??0?0100
0
Sinosauropteryx
000000010001?200?00?10?000??1?200101101?00?0?01010010?0[01]0?1????01100?0???????1?
000?0120?1???00000???0????????????????????????????????????0?0?0000010?00?00?0?????????????
????????0?0?0??????0??10?00?0?1000100?10??[01]0?01??01??10?000211?010?1000??0?10000
0002?001041??02?01[12]?0020?11210??0111101010210100101?20010??????????????00110??
??0?000?10001001000000101020[01]0??[01]1?11??0?02?0??0010111100?001002[12]0?02[01]?
??0??110??1?001?0??1300011?01000000011?01100201110 0?0110100000001??1???000?100???001

0?10?11?100??0??0?0?000?1?00??0?1001???00100??00?00001?0??0?00000?012?0???0???100?
1001?0??2?01?0010????????0??0?0?0001?00?0???0????000001??????0101???????1??0??0??100
?1??01000001?0000?0?00????0010?0?00?11??0?00???0?0000??????0001????100000?0?001?1?
0???000?0????0010?0000000???1000???0?0??00?0?0????0?0?1?10??????001?2?1???0?00?1???
000000000?0?0??010??0?0??????1???0??0??0???00?????1????00?0?0????0???0???000??000000
0?????000???0????0????000????000?00?0?0?00??0??00????01???00?00????0???1000?01000?00
20?00????0?0??0?
Sinovenator
10001001001?20010?110?100101?[12]10100[12]00000???0?011?????????????00???0???011010
?????????2??1?0???0????????111?1[12]?00100010001??2??????????010?000102?200??0?10????1?
1???0?10???????0????????????1?10?10?1???10??0?1???00??10???11????012111?1111100?2?01?1
0????????01???????????[01]??????0???1?????0???1????????????0???0?????????310000??21[12]????
?11??0??00?0?1000000000??002021?211?11110001?02100201?211?1000????0110?110?00?0?02
?0?2???12???0?1001110101102100 1[12]????0?11???????????????1?????0??0?1001??1?02?????01
?102?11010??1?00?????10????200?0[02]??0000??0?0??0??0?0?0?1?21??????0???????????0??2??
???????????????210?1????00000?0???0??0?0??0??0?????0101???????????1????010??1????10?011
0??0??0??????0??0?????10?10?0??0??????0001????0?000??0??1??????001??0??0?0??0?????0?0?
2?1??0???1????00????1????1??????0??0?????1??01????0??2?1?????????1?1?0?0100000001002?
10001????010000???0?0????0?0?0????1????01???0???????????????????0??0?0?????????0??00??1?
0????0?1????1?01?0???0???1?0????011??2???0??10????0???1011????0??002??11???00???0??
Sinraptordongi
0000000100011010000110000000000200001200200100000101121110010011100002000000 1111
010011000010101001001010011120?0?010110000101001001001000000000000000100100??111
00100100000001210110011211002101120111001001110010000010010?0000310??00000000000
00?00?????????????????????????0??????10????00??????01011????1200110000?30000?0????????
10?100?????0??0[12]112201100002[01]010100011?011022[01]10111010101001[12]0012010220
000011110012000110101000110010000010100100201100000110??????????000?001000?110000?
?0?101?11?000010100?0000110000?00000?00100?10000000??011000?00?00000?10?001??10??
02000101??20001?0??1?0?00?101011???00?000000??001000??010001200?1011??00001?000??
0?0??0?110?01?0000?001?0101010??0?????00110000 1000???0?0000000000000000 1?0??00001
1000010000???????0?0?0101000000??1?10???100?110?0??001000?0??01??00?00?0112?110001
10100??00000000?????????10?0001??000?1??00000010?0?100?000?011?0??0?0?0??0??????????
01000000?????0000?01001?101100000???00100?001?0?000??????0101000?00??000?0?0?0001
0000010?0001?00000??0??01?0
Sinusonasus
1000?00100?1???0?2?110?100??1021000?201000?1?0?0??0100002??1???0??????????????????
??012?000102?100?0??????????1??????????
??
???3?0?????21[12]320???1?1?0?000?2????????
???????????????????????????????????0200??001?220??????1????????1?012????????011??1[12]10
??11?2?00?2011110 11?1??????????????????????00???0?00?0???1??010????1???000?1?????????0?
???21??0??????????0??????1?0?000?1?2?0??????????????1?0??200???0?????????????????1?????00
?0???0????????00?????????1010???0??????1??????????1?????0?1??????0?0??????0??0???0????10???
?0???110?00????????000?????????????????0????0?????0??????0?2????1?0?????????????????????????

????0?0???1?????????????????0????1?00??????????????????????????????????1??????????????????
????0????0?????????????????0????????????????00????????????????1??0????????????????????????
???0???0?????????1?01???????002????????00??????
Tanycolagreus ??0?????0002??1??20?????0????????????????00????0??001?1[01]10?1??????
???????????????0?1???100??010??1010???????????????????????????????????0????????????0???
??21??????1?????????0?????0??????0?????????????0?????0000?00?0?1?????00211000001000020
?10000000100010310?000001??0200011010??0111001202100122?10?22001?????????0????????
???????01????001????????????????????????????0[23]11??01????????????01201??00000?01100
01[12]001?10113??01100?00000 1110110?2?1110000110?????????0?????0??010?0????1????0?11?
1????1000?0??0?1?0000?00?0?1??1000?0??????00?21???0??0000?0???10??1?0??001???1????2?
00?10100?????????1??1000??00????????0?????10?0000110?010?01?01?1?0????0???10????000?00
???11???0?0?????0?101?????1?0100?00??????0???00??????????110?000?0?001??????10???0???0
001?00???0?1???0?0?0??0??11?000000?????0?1?????0???0?????1?1?01????0?00????????0??????
?10???00??????????0??00????0?????0???01?????01?????0???0?????????0???00?????????001??????
0??????0???????????????00?????00??????0??????????????00??????1000?0??00???????0?0??0?
Tianyuraptor ??0?????00???0???0?11?0?????1??0?0?1?0??0?0??????????????????????????0?
????0????????0?0??0???000000000?????????
????????????????????????????????????10??????0?????1??0??00????????0??00201??11??110?2??1?0
0?0000??1??3????0?0010??21??120????0101[01]0110211?11121?1?21120??0?0?1??????[23]12[
23]?????0?1?2???0?2??1????00000?12010?211?12?1??011????????00200?10010???011???1?????
???????2?????????0?1???0??1?11??????0??????????1??????????0?????1?????1?????0?1?00?1??2?1
??0????0???1????1?1??1??0???1????????00?00???1????00??0???211??????????0????????????11???
00???????????????????0??00??????????10????????????????????????????0????001????????1??0?0?11???
????????11?0??????????????0???????00????????1???????1??0000????????????0????????1?010??????
????????????0??????????????????????11??????????0?1????????10???????????0??????00?????????
??2?????????01????????????????0????0??????????????????0?????????????0????????????????????0
1?????0??0????00?00????????????????????????1?0??????????????1??????????0?
Troodon ??????????????01????01?0??1????????????0???????????????????????????????0???0
1110110 11?????????????????????????????0001010 1110000??110012?????????00010001?[01]1[01]10?
0010???????110??1??????????????????????????1????????????????????????????????0??????????????
??????????????????????????0??0???02?????010??????????????????????????????????????4????????????
???1???????????1??????????????????????????????0???????????????????????????????????2??????????????
01?2[23]?001??0?[12]?11011?21??02011110 1111???????????1?????????1?0?11????????2??1??0????
0?01?1??1???0????????????????0?00????????0????01?0?000?0???1?0????00??????110??2?0??????
??0??2??2?1??01????????0?????01????0??????????????????????????0????????????????????1?0?1?0?0?
????0??????0??0?1????0??????????????0????001?0??1???????????0101?0??10????21?1?01????1???
???????????1??0?1??????0????0?????????10??????????0?01???1?????????????????????????1?????????
?????????1????????????00????0?0???00??0???????????1?????????????????????00000????????01??????
?10?????????0???????000???1??0???0??0?????1000????1??002???10??11???0?0
Tsaagan ?00000010001000000011000101010201001110000010010 1101000[01]1011
1110111020?0110?111010000200000?0100100200??0011?00110?101010?1?????1?01002100000
101?00000000???1?01?001?001???10???0??0?2?0??10?10?????1???01[01]???????????????????
????0????110?1??
??

??0???010????100000?????????0?0?
??????0?0000?1??????0?01????????0????????0????0?00????????????1???0???10?0??1???0????0
??0??????00?0?????????00?00??00??????00????????1000???0????00?????0???0?1???0??0?0???
0?0?001????0?????01?101???0?1???0?0010?100??000???????????????0100?0??????????0????1
001?0????00????0?0???0???????1??0?????????1???0?0???????????0?1??0?2???????????????????0
???101?????000?0????????????1????0????0?0???????????????00????????????00??00??0????0000
????0000??00???0??0??????01????????0???10????????000????1??112???0?0?????????0
Tugulusaurus ??
??
??
???????????????????1?1?11000?10???0?????????????????0???????????????1?0??????????0?1??????
??0110?0211000?01?1?012??110?101???????
????0????????0?????????????0????????0???0?????1????????????????????????????0???????????0?1?0??
?????10?1???????????1????????????????????????0???0???????????????0????????????1??10?????0?
?????????????????0?1???0?????????????????0???0?????0????????????????????????????????????0??
???0??????????????????????????????0?1???1???????1?
????0?1??????????????????1???????????????????????????????10??????????????0????????????????????1
??????1??0????????????????00????????????????
??????????????0????????????????????????????????0?
Tyrannosaurus
00010001000210201201100100101020000121000101000011011110111101111011200111021101
011102011000010021010100001100002101000021110010100100000000000000000000001012100
010110001100111001011120?10?20011200100011 01[12]110100000100100000022110000010000
1001000100000010041000100002?01?00011010000[01]01000?0??0?122?????20010????0301100
001012110100100110000100101112112101200002011110111131221011011110100000120002 21
000111100111000100123000110012110011100100101100000 1100??????0101000011100 01100
100?10101101000111000010011 0000?0011000020000210000000010001000400000121 10010?0
01001002100??20010000000000?101011000111000?00?1001010010000011000101100 0100100
11000100010100111010000000100 11111?000???0?0021011100000000?010001000000000?10000
00?0?0010110111000 00??000000001000010011100001000110000000000000000001??0000000112
11010011011000000000000000000010?0001?0000?1?001111111020001?0??0?1200??010?0010?
?????????01000000??0??00000110010111100000?00000000000?0?000000?000101010000 00010
1100?00100011110?000200000 11?0?01100
Unenlagiacomahuensis ???
??
??0??1??010??011??1???1??32?1??11??????????????
0010[01]0?0??2??0???41??????????????1??
??????????0101002000?0120111?12?02?1002210200?100122??1?0110??0?100?10??0????1???1
???1????????0?1?1???????0??????????
??1???0????1??1?020??????1001?0???1??????????????21?0????????1????????????????????????1?1?????
???????000???????????????0?0??0???1???????????????0???0??0?????????1?0??0?1?1????????????
???0???1??????1????????????????????????1????00?????????????????
????????0??????????????1????????0???1?1??????????0??0?0110?0????????????1??0?????12????????
01??????????????2?????0???????1????????????????0??????????????????????????11???????????????????????0???

????????????1????????????????????????1?????????????0??0????

Unenlagiapaynemili ??
???
??2??010???1???11?????????????????????????0?1
000?010???0???12??????????????????????????????????
?????????0???????????1?1??0[01]??012[12]11200???
??????????????????????????0???????????????????1?????0????????0?????????????????????????????
????????1?0?????????0?1?1??????1??????????0?0???????1?????????????????????1???0????????
00???????????????0?0????????????????????0????????1?????[01]???0????1?????????????????????
???1??
??????????????????????????0??????????????????????????????????????1???????????????????0?????
???1?????????????????????????????
???????????????????????????????1??????????????????0????

Utahraptor ??0????10001101???0?????0???
???????????????????????????????1??1????????0?????????????
???0??1??10?2????1???11????02?????????1??????1?0??0
??2??????????????0????????10???1?0?
0???0?00???1????????1???1??1??1???[12]?1??0?1010?10?00???[12]200?????????????011??00??1
23??????1?0??1????????0????????????[01]???????????0??????????????????0?1????????0????0?????
?0?????0?0??0????20????0???0????2??????????????2?10?????????????????????????0???0????????
1??????????0??????0???????????????????0????????????????00????????0?????????0????0???0????
???????10????????000????????????0????????????????0????????????0??????0?????????1???????????
?????1?????????????0???1???10???????????0110???????
????????1????????????????????????????????0??????????????0???????????????????????????????00?
???????????????????????????????????????1?????????????????

Velociraptor_mongoliensis
0000000100011?001001101010101020000110010001001011010010101111001101201011010100101021000000001002002000001111?00110010101?010?1101111002100000010[01]000000000011
1?01100????1?0??0?0??????2?11120?1021?10101?1?11?010?0011111110?0122110011111110020
?10100010[01]000102????000011??21??12010?0011100120210?1111112?2112001000410000010[
12]024200010?102?0000210100[01]001100012011121001211000 1?[01]2100101020001011112O
0120?1100000?1?1?01200112301010011010101100100110110101201 0???????0001?010011010
0000010?021110001?102?0000000010010100100100200001?0001000020100100000000102 1100
?0?001?010001010?2001?0000?00010?00?00?00000200000??100?0?01000000?11011000???00?
0?00???0001100100110000111110?01000100?011000002101?000010?00?0010010001000?11?1
0?00000?0011110001010000??11001?0100100 1100000?0100000100100101?0100000 11?10?1100
??201?0001?0?1000020100100001002 11010000?0101011000000?0110000??1??1?12?0?00?0000
01??????????0?00?000?????00001?00000101?00001?00000010000?00000101000011102 00?000
100?0?00001000??1?00?112000000000?0?000

Xiaotingia
0112??11?0?0???0?0?11??00???2?3000?10?110?0?1?????01?0??1???1??01????????????????????
?????0??0??000??0012?1??0?1??0??0??1010?????001?0?
00?0?????????????????21???10?????????0???1??0???10??00200??101?11???????????1?????????20??
???0101??21??1[23]??????101?012?21??1002?12?01?2??????03????11??????????1??????????????0

?1??0100??120101?1??1110??2???????2?????1???0????????????????0??????1??????????1??????
???????????011?1?0111??1???1???????01?????0????1??0?00?11?001??0[02]?0?0?0??????????
1??????????2????0?0?0??0??1?00???0??2?????????0??1????1??1?2?11???01????????1??????????
????0??????1??1??????????1?????????????0??????11??????10??01??11?????????????10????????
?????????????????????01?1??1???0???0?1??1?0???0??????????10??0?????????00???1?????????
??????0?????10??0????0001??????1???0?10????1???????????0???0?????????2????????????????
??????????????????????????????0???????????00???00????1??????????1110???0???0????00?????
???????????0?????????1?1???1????001??1????????????
Yixianornis
0????211?11??2??0?0???001???????????
???1???1????????????????????????????????????0?0?101?021????200???????????????????????0?
??????????????????????????1?????0???????1????2?00?201?111??10102002?1011??2??0??11??10
10001102 1?11300???21011210 1101?121?????20011??2017?2?????????????????????11???10????
0000?????????11?11?0??00?0?20?2010201??00?0???00?0??1?????????2?2?2????????1210???111?
011???00???11?00?011?11?100?0??????1??00?0??????0?0000?1??10???0??0?0???????1????1?1??
???2111?2???0??10???0??????3110?0????????????????????????012101?0?00?1??????????0?000??20?
??1???0????010?0?2??????0??????????????????????00?????1??00?1?11???0????????0?110??????1??
???????????????????????????1011101?100????0???0?0?1????001?????????1?????0?0??0????11?0
0??????11?0?111?????00??2?1???01???1??????1??????1?1?11?0?????????0???????????????????????
???1??????????00???1?????????????0?11?1?00010?????0??????????????1??????????1?11??00??10???
???????2??0???????14??1?1????????002??11???????0????
Yutyrannus
0000000100?20220?20110010???20200001211 1211?00?01111?10111101111101?20??11?11?001
101110?1?0????021010????????????????????????????????0000000000000?100100101?10101????
?001??10???0??1?2?1??2??01??????0????1???2???00?0????0?00?211?000??0???10?100??00?2?
?0?0310??1?0011?010??1101???0110000??21?111101?1?2001?????????????12?0?1[01]0????00??
10??010??011?3112??020?00??1?10?1?101?2?[01]??00?1??0?0?10?1????21??0?1?11??01?0?0??
?12??001001?0??0?11?0???02??1??0?0?10?1?0????00???00????101??00????1?011101???12???0
?000????0?????1?0??10??00?001????0????1??00000?00?10????010????002??0??2001??100????
????0???????0?1??00?0???10????1001???????01??1???1????10???0100001 1?0110?0???00???001
0??1?00?0???0??10????0?0??00?0100?????????0????0???0????00??1?0???0??????00?00?010????
????11????10??00???00??????0????????????????0??????????0??0????0?????????0????????1???000??0?
??????1?10????????????????0???0?????????10??????????????00???????????010??10????0????????????0
00??0?????00???????0???1????00???01000????100110???00012?0???11???0????
Zanabazar ?0011101000102[01]01??010?001101?21010?1001010101 00?1???0?01?1?1?
00??00011011010100?0101????????????????????101012111?00???1100?2??????????0001000101111
0?0010????????110??
??4101?0???????
???111????????0??
?0??23010?????2?1????1?1??????????????????????????0???0?10???00?100???????1??0?0?1?????1?
?0?1??00???00?0?????????00?????????0????0??0???0??????1??????0???????0??0???0???????????????
??2?1??????????00?0??????0???????0????????010??????10??00???0????11?????0?11?0???0?00?1???
??0?????10??1???00110???0001????????01??11??????????????1?1?0??1?????????????????0010?0????
10???????????1??????????000??????0??0??0???????????????1??0??????????????????????????0???1??????

?????1?????????????1????0?1????0?0?????????????00??????????0?0?100??0??????001???1??0??
0??????????????????????????????????????1000???????002???1????????0??
Zuolong
0000?00100?110100001101?000?10?000???0?1???????????0???0?10?????0????20??111111?1?0?
0????010?0100?1000???????????????????????????1??????0?????[01]0[01]0???????????????????
????????0?0?0????1??111000?2001??0?0??1???0????????????????2???????????????????0??1??
0003?????0000????????????????????????????????????01??????30000010?0????010?10??00??0
????01?10?????02??0011??11????0??0?001?????????????01100?200000??101?02????????1????0?
??01000101?0?1?0????????????????????0?????0??0??00??0?1????0????????1?????0?0?01?????
????0??1000??000??0???0??0?00??0???0??0?1?0????20?????00??0????????0????????????1?11??
00?00?0??????????0???0??0011?????0??1?001??0?????0???00??????0???00?11???1????00?????
??11?001?????001?0????10?????0?00??????????0?1???0?????0??0??????01????????????0???1??0?
???0?100??????0????0?????0???????1?1??????0?1??1?????????000?????10??001?????????00???0???
1??1??0????00?????????????0????????????????????????????000???????????????????????????????????
???????????10????????????????????0????????????2???????????????
Eosinopteryx
010??001?0?1?2???00?????0???????0?????????00??1??0?????0?22???1???????????0??????????????
???010?100102?2???????????????1????????0??????
?????????????????????????10?????0??0??????????????0??201??1???11?????1?0??0????????[12]???
???0001?0?1?0122??0?0101?0120210?111?1???2111?????0?????????31223??????1?0??00?2????1
??200???000?0??11??1???10??0????1011200?11010??????????????????????????2?????????01[01]???
1???011?0?00?2?11??0???1??1000?120?????0????0?????1?10?0??11?001??2????0?????????????
??????????0????????0?0????????00000?0?2?0???????????1??1????2?01???00?????????0???2????00
?0??0??????????1?????????????1?????????????????????00??????1?0???0?10??????????????0??????10?
????????????????????????0???????1?0000????0????0???0???????????2????????????????????0??0?????
????????????1?????????????????????0???????0???????????0????????0????????0???2?1????????1????????
??1???????????????????0????????????00???????????1?0???0????0????????????????1?0????0?0??00?10??
?????0?0??????????????1?????????????02??11??????????0?
Aurornis
0010?0010??0?2?0?00110?110??1???000?000?00?110?0?101?0?02???1??0???0?1?0???0??????0
02????0???????00100??010??00?02?2?0?00????0???01????????0?0
???????????????????????????10???????????????????????????201??11??????????????0??0??????2??
????0100?021??120????11010?1?02???1110????20????????2?2?0???21223???1??0?0?00001?00
01??001???020?0??01?11????01??????1000?0??110?0?????????????????????????2??????????010???1
????11???0???011?00?0?1???????????0????11?00?000???1??0?0??11???1??0????0?0????????????1
0?0?????0?????????0?????????????00?0?2?0????????????1??0?????001??0???????????????????01??02?
1??0??????????1?????????????00?????????????0???????0??1???1?????1?10????????????????0???????10??
?????1??1??0??????????????1?????????0?????0??1??????0?0???????????1??0???????????0????????????????
?????0??????1?????????????2?1???00?????????????????????????????0?????0???0???2????0??????1??????????
????????????0?????????????????????????0???????????0?0??00?????????????????????11???0????????????0??0?????
?????????????0????????????1?????????12??1??0??????????
Jeholornis_curvipes ??1???????????????????????001
??01100???????????????????0???01??12????????100?1010???
?10????0?0?10?11??????????????????????????????0???00????0???0??01?????????0011000110001?02?0

0?1??10?110[01]10???0?001???1???3????1210110111100?101?1????0?2?????1?????????1?4??0
??101?010??0??00?????002????01???10?12?0?02[12]?0?????0002?1?000101????0???01???01??
??102???1???1111?1?00?000000000210110112010?????????0???????0?0????????????2?11?0???
???00?1????????0??1?????????1????2?0??21??0??00???000?10??0???0?00??0?0??1??0?2?1101?
00?1??01???????000??20?????1??0?1?01???01???????????10????1??0?????00???001??100??11?
?????????0???1?????????0???????1??????????????0????11??10??100????0???1?0????????2?0???
????0?????????0?10?1??00?0??0?1???0??????????????1???010??????1?12?0?01???????????????
1?????0?0???0????0?????0???0???????????1??10???2??????????0010???????????000??????0?011?
11????10????1?00??10?00??????00??0?0?????1??00?????10????0??1?1??????0??1?
Jeholornis_prima ?????????1[01]????0??0?????????????0???????????????????1?0?????????0???0?
???0?????????????????????????0??1????????????02????????
???????????????1??1????0????0?0???01?0????100?[12]1?2?
??1???200?100?[12]00?0?[01]000??1?0?21????211?1010?[12]10???1?????????2?????14????[01]?
?3??[34]???????1?1??00?010???0?2000111202020???1002???001?0?20?1010221??????????1?????
????????1[12]??1????21?10?1?0?00????????????1?1???1??1??1?10??????0???????0????????1???
??1??[01]1?????????1??????0?0??0?1???????10?????????[01]1?????0??100?0?1??00?????????????
?1??1???10???00?0????????????0?0?12[01]????1?????0??1????????0????????0??0??1???00???00?
??0?1???000?1????????????0??0101??????1???0?0?0?????????????????1????10?000??1?0????0??1
010?1??????100??????????????10??0??0?10?00?????????0???0?????????1?1???0?????00????????
01?????????????????????1?????????0????????????0??????????1??????1?????????1?00?0??????????
?000???????????0?1??????0??0???????10??0????????0??0????????1???1???????0?0??1???????????0
????
Jeholornis_palmapenis
0?1??????10????0??????????1??????0?0?????????????????0?????????????0???0??????0????????????
???1??12??????????0?????????????????????????0??
??????????????????????????????1???[01]???????????0?0??
???3??[23]?????????[01]1?01?11?0??0
?20100002011??10?12?0?000?0??1?10?[01]?[12]????0?0????1??????????0???0?1[01]2????????0
11?0?00?0011?0?10?2101??????1????10????????????????????????1??????0??1???????????????0?1
0?0?????????????????0???????0??0????0?0?1??0???????0????????0????2?1?????????????????0????
?????????????????????001???????????????0?????????0??????????????0?0?????????????0?????1????
????0????????????????????????????????????0??0????0????10?????????2???????????????0???0??0?1
?????????????????????????????1?1???01????0?????????????????????????0??????????1??0???1????????
??????????????0???0???????????????????1?0???????????????000?????????????1??????????????????????
??????0????0???????????1???????0????1??1???????0??????
Jixiangornis
0002?1?1?1?0???0?????????11???????0???0?100????1???????????????1??0??????????????????????
???001??12????????1[12]???10???????????????????
??????????????????????????10????????????????1???????????????111?1?0????2?10011?1??0??0????
0?0?01?021??130????21010011?110?101?1???20011?10005???01??[23]1??30??????????00?????
?????????????????????0????????????1??1???????111[01]???0
?0?011?0?00????1?01?101?1????????0???????????0?????10????0??0?01??????1?1???????????10??
0???????00?????????10????????10?00?0??????????????????????1???02?01?0?00??????????????0?0???2?
0?0???????????1??0????????0?????????????????????????00?????1?0?0???11???0???????????10?????????

??????????????????????0????????000???0????0???0?????????01???????????????????????????????
????????????1??????00????????0????????????????1???????????????1??????????????????????????
???????????????1???????????????0???10??????110???????
?????????????0???1???????????0???1????????????
STM9_9 ???
???
??????????????????????????????????????1???????????????????????0???0?10????1?00???2?100
1??1?001011???01?00?1??100?210???21?11?1??0?0?101????????1?????1[56]????1????????????
???0???????????????????????????????????00?0?200????????????00?????0???????????0?111????
???1111?1?000001100110021?1100?111??1????1??????????0?0??????0????0?1?????????01?1???
???00?0?1????1???100?????0??00??0???0??2000?0???1??????????????1??0?2?01???00???????
?????000??20??????????????1??????????????????????????0???????????0???0???11??????????01?
?10??00?????????????0???0?????01??????????????0?0
??0??0??0?00??????01?????????????????????01????0??????????1???????0???????1???????0????
??????????????????????????????1??1????????????????????????????0?????????????????????????????
0???0?????????00??????????????01?????1?????????0??????0?0????

Matrix on coelurosaurian phylogeny after ref.20.

Dromaeosauridae
0000000000000000000000000000000000000??0000000000000000000000000000000001????1
?00?0000000000?000000000000020?[01]??00100000000000010000000000000100000000??000
?000000000000000[01]0000000[01]000000000000010000[01]00?000000000100000000000000000
0000[01]000000000000?0000000000[01]00000
Archaeopteryx
0000000000000?0010010?00?000??0?000???0000000?1000?100000??00000?00020001????0?0
0?000000000?0000?0?0000000????????????000?0001100000000000???0000000??001000001
00000000000000200000001?0001?010002?10?00000?0001?0??0??00000000000000?0?00?000
100?0100000?00100010
Jeholornis
[01]?23111?????????????0??????????????00?????000?00?1??0????00??10000????010000??????01
001?000001??1201?0??1??00[12]??????????????0000000000?0[01]00??01011110?000001211001
000010002000010013000000000011?101?010001????0???0?0??0[01]0?10??1100000000??00?020
0?0010?00?1?001001010000?0
Sapeornis
1010010[01]?0??????0010?1?0??????????1?0????00??0??1????100[01]01??10010?0024000000?0
0?00?011000000??020?0???10002????????????????00??0001100000???0100111????00??21100001
000?0020010101030000??0?11010111?001010??0100?1000?0?111?1100000000000?0000?001
1???01?0000000101010
Confuciusornis_sanctus
10231111?0?????00010110000120???11?1121010?1100?101?101[01]01112001000024000?00001
?01?0?0?0000???020?0?00000002[12]10??01121000000100021 0?00000101000010021000121 0?
000?100?00000002100200000000?101011110003101120101?1?0010000?111000010001?210000

00001100?00?0000000000110
Changchengornis
1?231111?0????????1??????0???????1?11??0?0??100???[12]?????????2??1???024??0?0000??01
?0?0?000????020?0??0?000021?0??01??10?0?0????02??????????????01?????0????0?0000?0??0
00000021002???0???????????[12]???03???????????0?????????110000?0?0??2??0100?0011?0?00
?0?00?00100110
Eoconfuciusornis
[12]?231?1[01]?0??????0????????????????1?01??000??100????0?0?00???0??1???0241???0??0??
00?0???000000?020????0000?02[12]???????????0?00000?100?0???000????01?????0??01??0000
1?0?0000000210020??0??0?????????1000[12]??????????0??????10????0?000000002?00200?001
000???0??00010100100
Jinzhouornis
0023111[12]?1??????00???1????????????1?11??010??10???????0??0?1110??????240[12]0[12]?00
0??0??0????????????00?????000?02[12]10??0?1[012]10?00000??021020[01]0???11?0101??[01]?
??0?01????01?00?000000021002?????????????????????0??012??10??100??0010??110000[01]00??
0?1?0200?0?10??????0??001000011?
Confuciusornis_dui ??23111????????????????0?????????1???????0??1??????????????????[012]?
???14?10?0??0??0??????????????????????????2[12]?0??0???10?0?0??00021?????????????01???
??0???1??0[01]00?00?000??0021002?????????????????????????[02]?????????1???????10????0?
00????0[12]00?0010?0????0??0?1000011?
Boluochia ?1[12][02]??
???2?????????1?????????????????????????????[12][12]?[12]??????20???????????????????????????
???110101???02??????????0???????????0[01]?100100
002??3200?0011??1?????????2?20????
Concornis ??01??11????????
0?01???????111110?02?11?00?1?????1112221222111?20?00111??0[01]0211??????1????1112???
?????????0??0???30010[01]0122????????????11?1?[12]??00?0??????????1111???????1110?0010??0
[01]??0000?001???0113 1110?0?2??1??
Eoalulavis ??00111200??2?0???????
????????0??111110102111000000 10?111?312?0?0010?40?00111100102110010011111111?21?1?
????????0??1?01?001????2???0?????1??????????????1?1??????????????????????????????????????
????1??1121111?????????
Cathayornis
[12]0[12]0??0??????????????????????????????000????00?????1??1??1??0???11?2????240?0?0111??
?1111?0??20???000001?0111?322122311??20?0011?1?010211[01]010111110 11????110?3110001
0?[01]000130010101220000 00??11??????100????12????1????[01]?????1?1????????????????????
??????0?1111111????????
Eocathayornis ???0???2???????????????????0???0?????000????00?????1????????00????????????
???????????11?????02????000000??0????221213 11??20??01??1?000???[01]????11???10??????0
?31??0010??00012001000 11???
???????0?111111?????????
Eoenantiornis
[01]010??02????????????????0?????????00????????????1???????????????[012]????24020?????1?
0111110101?11000?001???11122[123]?2??110?2000?1??1?000??????????????11?????10?31??000

0?00001200101012 2???0???????????[12]??001????1?????????????????11??0?10??????0??1?0?1
110011[23]1011?[01]?1?0??2
Gobipteryx
10[12]3101200021?10????????0???0?1??1?[01]0???10???10??????1?????????[012]10??[12]401?
????????1111?010?1???0??0010??111[23]?????????????0??????0??????0????????11???111?????
????0??0?01?00??????20???????????????10?03???????????10[01]10?011?110010?100??11?0??1
?1??????????2??1????????
Longipteryx ?122111???????????????????????????000????00??01?1??1011?????????2????
24??0?01?11?01?1110100????000?000?111132[23]?21111??20??0110??0002??[01]?0?011?1?11?
?????0?01100000100?012001000113 0000????1111011[12]11001?0?[12]1?10????0????0?1?1?0?
1000000001?32000001???1?1211?1?2?2????0
Longirostravis
[01]022??1????????????????????????????00?????00??01?1??1?????0?????01????24??0?01110?011
1????00?2??000000??0111[123]2[123]?224110?20??01??1??00???[01]?????????10??????0?31??
10?0?[01]01012001??0122??0?????1??10?1?11????0?1??????1?00?????1?11[01]?10010??0?2?0
100?00????0?1??111?1?1????0
Neuquenornis ??0???11????????
???????????1?1????021111000????1?1111230[12]?1?1?????0????11?????1?01?01????11?1??11?
?31??10?0?[01]0001????????????????????????????????????1110???????1????????10??0?1??1?2?00
?011001[12]????1??2[12]0???1?0???
Pengornis
00[01]01101?0??????0??????0?1???????000???0?0?????11?2??1??0?1?10?1????240?0[12]???0?
?01?11???00?????0??02???11?2??????????????001?0100101??[01]?100?1???11??????1[02][23]11
1???0?01001?0010?0??3???????????????????02?01???????1??[01]?01[12]11?010010010010120
0201?00?????011?101?[01]011?021
Eopengornis
0000110[012]?0??????????????0?????????00000?000?????1??10?0??00??????????24??0?1?????0
1211101000???000?02?00111321?110?1??10?0010010000??10?0001????10??????0??1[01]?00?0
?010012001010103???????????????????000???????0????100??????0???0001????1??0101?0012?0
00111101?1011102?
Protopteryx
[12]?[12][02]1??[012]????????????????????????????00??????00????1???????10?????01????14????0
??1???1111?0?01?[12]??200?00??0111322111011?[12]10???1????010?????????????11?????????
1??000??[01]0001100000010???00????11?????????0[12]????1?????0????????????00??0010??00?
?0[01]01?001110011?0100?[01]101121?
Rapaxavis
[01]022??1[012]??????????????????????????000???0?0???1?1?????1??01??11?1???024??0?0?110
?01????0?00?[12]??0??001???11?322122411??20??0100??0000??[01]0???1111?10???1??0??11?
00?010000130011?01230000?0??111101?111?0100????????0?0[01]?0?11??100?10000??002?01
00?0011??0?121011?10110210
Shanweiniao
[01]?[12][23]??1?????????????????????????????0????00??01??????1???????????????24?????1?1?
?01?11???000[12]??000000??011?22[123]?211?[01]??20???1????00????????11???11????1?????
????????????20?11???22??????????1??????1????0?????[01]0??1??????????110??0000????1?[01][

01]?0?0?1[01]?10?12?111?1011?210

Vescornis ?0?????2???????????????????????????0000???00[12]001?1??21?1??0???1??2?? ??2????????00??01?1??0?02?[12]??000001??011?322120111??20?00110??0101?1[01]0???111111 1??????1[02]011010?00[01]0?0130010[01]01220??0???????????????????????????0??[01]??????? ?00??0010??011?[01]201?1011??11121[01]11?[01]1110?00

Vorona ??? ?? ??10112011?0001110111111[12]00010001 1?21000000? ????????????11???????

Schizooura
0123111[01]????????????????????????11?0????10??01??0?[12]1?00?00?1??0[345]?1??[12][34]? [12]0[12]?000??01011?0?1001??[01]2?00????11012[23]0[12]?011??1??1?000??0[12]0010[01]0? ??01?0?10?????00?31??0000?001?0[23]001010103?0??1[01]1???0101?1?0103?????[12]?0?????[12]???????11?010?00001[01]2?011000010?1?01?1110?10000001

Jianchangornis ??1??????????1?????010?200[234]0?? ?????????????0101110010020?0200?0?0121?02[23]031001??[01]??01000000100100000001?011 1?????0???1??01?0000000210101010 3??01?[01]?????????[12]?000????????00???0??0?10??1120 ?00?0??????0100?00???01010001110000?0?1

Archaeorhynchus
1013111[12]?????????????????????????1?00??00?00??1???0?????0??1???2000??[34]0111?????? 01011?00?0?20?[01]20000?0?21002[23]122211?[12]5???1000??010????0???0110?10????101?01 ??000??0??0?21010???230000????101100?[12]10001??????????0?0[12]00????211[01]010000?? ??0?0100?00????0010101100000?01[01]

Songlingornis ???[01]??????????????????????????????000?????0?????1?????????????2??????? ?????????????10?????101???2????0???21?02301231[12]??51?????????????????????????????????? ??0??? ??????1?10?01??????????

Apsaravis ??????????????????????????1[01]?????1?1????1????????021?1?201??00?4?? 0?24?10????????10111000011111211020 01????[12]30???11?????110010001011100001111111 0 121101?31100111011 0???1????????100011111?111012??113101[12]?110??1???20121??11[12]?1 11001?1?200100?00??????1??12110??0????

Yanornis
10[12]11102?1?????????1??????????????1000???1?00?01?1??0?????01??2003??0??????????????01 011110100?0?220001??121?02301121[12]??51011001??010110[01]????0100011?0?11?0?311100 0000001021010101231010 0?11?10100?11?003??1???????1?0??0??0??111011?00[01]??12?0100 ?0011??0?101001000000000

Patagopteryx ????????????????1?11110001111????????????0??000?11211011001 10003110 1??120?????????1011100000?0?1210010???????2[12]????10?????1001???1000?0?00000100?0000 ?0?0??????????0??1?1?20?00???20?0001000001110121?013?0??20????1001?0?011111201010011 10000?00?0001????10??110100000?0

Yixianornis
1121????????????????????0???1?1???0?0???110?????1??1?1??2011?1003??0?24????1000[12]001 01110010010?220001001210?2301121[12]0?51??10??11??0?0?0?11001?0?100021?0??31110101 00001?210101010 2100?1??00?01??11??003??1?2?101?100?????????11[12]01?0001110?0010??0

011?110101011?0000001?
Gansus ???121?1?2011110040010
24?20210000?1101110010020?320???0012100230210100?10?11001??010130?1???01001110011
1?0?312?01110001?131010001321?0011110101011210003001[12]?1101?100[12]2?0?0?2112011
10010102?020000011??001?10110?010000?
Ichthyornis
21[12]???0?????????111111??1111???01001???10021011111[01]11012011120?[34]?0102401021
000???101111010010?2210110012?002300??1212?0111001100101202 10?1010001100211 01?31
21012101001031111????310?01011??1101121?11?10122 1101?10022 1110?2112011202?01?2102
00000??????10101001?1????0
Hesperornis
11231103?1021011?111111000111?1?10100??100010101?02100121111200401 0?130202100010?
0?01?00?0010?020???000200?21?0??0011?00??????0?????1000000??????????????????????????
???????????1000121101110012101131021222011101221111121120112?21013223220102??????
???01?020?0?0?0
Parahesperornis
1123??0???0?10??1?111???00111????01????1???????1?0210012111200?????1?????10001??0?0
1?0??0010?0?????????????2???????1??
???????????????????????????10212220111012211 11???1201?2?2101?223220002?????????????2
0?0?0??
Enaliornis ??????????????????????????11???????????????????????21?0??1???2??????????
??
?????????????????1??????1???????2?????101221201110122 1111???1201???1101???0120000???
?????????02???????
Baptornis_advenus
1??3??????????????11???00?111???????0??1?0?10101?021001?11112004010?13020?100011?00
01?0010010?0?????0??????2[12]????0112??0????????????1000000???00000100??????????????
???????????01000121101110012101131011212011101221111211201?2?1101322 1120001??????
?????02???????
Baptornis_varneri ??021?01?1??1???????????
??
?????????????????10????1???1100?210113????2201?10122111 11211201?2?1101?221220001??
????????????2???????
Vegavis ??2????????????[56]???
??????????????1011?00?001??[01]20?00????????????????????1?0???????????????????????????0
??????????????????????????01???1?????01??2???????0112110??1?02?2?1????12011???3?1????1?
??0???????10?01??????????
Anas
21230113101111111111111111 2112111?102100001011?1021101211110006012024020210002 01
101111010020?22010000121002311100113401110011110003011111010000100111 01231201321
11111031011??12311011111010111121011310112110111002220111211201120130132102 20000
101110101121010100010
Gallus
112311131112111111111111111 2112111?102000001011?10211012111100160120240202100020 1

121111010020?2211000012101231101011220111001111000301111111000110011101131201321
02101131011??121110112110110111210113101121101110022201112112011201301322012010010111010112101010010

Fortunguavis ??????0????????????????????????????????????00???0????0?110200??1?000??02
[34]??0??1110?0111110100111002 0????0111122??2??1???300000??10000??1??0001?1??11?????
1122110000010010120010 00?120?????????0101111001 2?0????????1???10???1?110010010000?
0?02?100011???11?10?1?11100111

Qilianiа ??
???
?????????????????10????00?10101?2?0003?0??11100?00?110000?11100?001001?11?[01]?20?0?
1[01]??????????1?120021?

Shenqiornis
[01]0101102????????0??????0?1???????000???000???1?1???11?0?01??11??????????0???????01?
11?0?01?[12]??010101??011?[23]2[123]?[12][12]3?1??20??01101?000?????????1???11????????
01??0010100101200101011300 00??????01?111???01??????????0????????????0?1011???0???[01
]?0?0?1???[12]111211[12]01??2?0?21

Sulcavis
[01]010??0?????????????????0??????????000???0?0?????1?????11?????11?[12]????24?1010110??
01????0?010[12]??0[01]0001?0?1113[12][123]????????????01????010?????????1???11??[01]???
0?[02]1??0[01][01]0?00?0120010101120?00?[01]?????????[12]10?????????????0?00?0?01??110
010010??0[123]??0101?0011???11211[12]011110012?

Bohaiornis ?0[12]01102?0??????????????0????????1000???000??0011????1?120?1?110[0
123]????24000?01101?0111110?01020?0[01]0101?0?11122[123]?21311??30?001??100001?10?0
?011???11?????10?0110000010010120010101230000??????01011[12]??000?0?1????0?0??0?0??
10?1100100100?01?[01]0101?0?11??101210211110 00121

Parabohaiornis
[01]0201102?0??????????????????????000???000??0??1???01??200??110[12]01?124??0?01101
101?1110?01?11?010101?0111132[123]?20311?[345]30?001??10000101010001110111?????10?0
1??00001001012001010123000 0??0?010101 1[12]1100000??????0?0?00?00010?100010010??01
200101?0?11??1112102111100022?

Longusunguis ?0[12]01?02?0???????????????????????000???000??01?1??[12]?1??20??111
0[123]????24?00?0110??01111101020???010001?0111?32[123]????1???[23]00001??10000??10?
00011???11?????10?01??0000100101?00101012200????????01011[12]??000????1?100?0?00?00
010?100?1???000?1??010100?1????1121020 11?100221

Zhouornis
[01]0101?01?0????????????????????0??1000???000??0??11?[12]?1??20???110?????24??0??1?0??
011111010101??020101?0111132[123]?2?3110??0?000??10000??10?00011???11?????10?31??00
0010010120010101220000???????????[12]??00000??1?1?0?1?00?0?01??110010010??01??0101
?0?11??11121?2011110012?

Piscivoravis ????????????????????1????111?[01]????????????0?????0??12110110111210[34]
????24020??0001101?11?0??0????[23]20????0121002301221???51?0?0????1????0??????1?0?11
?????00?31??00011000102101010112101010????01011210003?01121?01?10?22????1?1120111
00[01]1?0200110000101100 1?01???0001000?

Hongshanornis ?020?10??????????1????????????????00???101??0????????????????1???????2

4??????01?0101110010????[01]211?0?0121?12??2201[12]?210?110?????10???2????0????11???
?????31??00?0??00?02100010101??????????0??01[12]????3???????????0?[12]???????1120?0?0
0?0?0??0200?0011110010101100 1100001
Longicrusavis
10[12]01?0?????????????????????????????00???101??01????11?11?0???20???????????????????10
11100??????[12]21100?01210?23?120110?10?110?01?1101202 1100010001100???0??31110??00
010102100010101???????11??1???[12]??0?????????01?10022[01]121?1112011100??10100100?
0011??0010101100110000?
Archaeornithura ???[23]???0[
12]4??1110001?0101110010110?221100?0?2101230[12]?111???????0??10010??02????0100????
????0??[23]1??0[12]?0?000?02100010101?0????????01001[12]???03????2??0??1?0????2?0?111
0?1?01???[01]??0100?001111?0101011?01100001
Parahongshanornis ???1?1????????????????????
??????????1????0?10????[01]211???012101230220011??10??10??1?010??0?????01???11????????
31??01?0?00010210001010 1?0?????????10??[12]??003???????0????00????????1???11?0????1??0
100?0011??001?1011?0110000?
Tianyuornis
1020??0??????????????????????????000????01??01?1????????????????????4????????11?10???
0?10????22??????1210123?22111??10?1100???010??0?????01?0?11????????[23]1??0??0?00010
2100010101???????????????????????????????1??[12]??12???111011?01???[01]??01?0?0?11??00
1?1011?0110000?
STM9_9 ??00????????[01
]???????00???????0??0????000???000??????0000????????????001??00010??00?0??01?0?10???
?000?21??000000100010 01????[01]30??????????????[12]??000?111?0????1?001??00??1100000
000002??0100?001????00?1?[01]111?10001?

8. Supplementary References

1. Xu, X., You, H., Du, K. & Han, F. An *Archaeopteryx*-like theropod from China and the origin of Avialae. *Nature* **475,** 465–470 (2011).

2. Chiappe, L. M. in *Mesozoic Birds: Above the Heads of Dinosaurs* (eds Chiappe, L. M. & Witmer, L. M.) 448–472 (University California Press, 2002).

3. Godefroit, P., Cau, A., Hu, D., Escuillie, F., Wu, W. & Dyke, G. A Jurassic avialan dinosaur from China resolves the early phylogenetic history of birds. *Nature* **498,** 359–362 (2013).

4. Mayr, G., Pohl, B., Hartman, S. & Peters, D. S. The tenth skeletal specimen of *Archaeopteryx*. *Zool. J. Linn. Soc.* **149,** 97–116 (2007).

5. Chiappe, L. M., Ji, S., Ji, Q. & Norell, M. A. Anatomy and systematics of the Confuciusornithidae (Theropoda: Aves) from the Late Mesozoic of northeastern China. *Bull. Am. Mus. Nat. Hist.* **242,** 1–89 (1999).

6. Zhou, Z. & Zhang, F. Anatomy of the primitive bird *Sapeornis chaoyangensis* from the Early Cretaceous of Liaoning, China. *Can. J. Earth Sci.* **40,** 731–747 (2003).

7. Dececchi, T. A. & Larsson, H. C. E. Assessing arboreal adaptations of bird antecedents: testing the ecological setting of the origin of the avian flight stroke. *PLoS ONE* **6,** e22292 (2011).

8. Wang, M. *Taxonomical revision, ontogenetic, ecological and phylogenetic analyses of Enantiornithes (Aves: Ornithothoraces) of China* (University of Chinese Academy of Sciences, 2014).

9. Dyke, G. J. & Nudds, R. L. The fossil record and limb disparity of enantiornithines, the dominant flying birds of the Cretaceous. *Lethaia* **42,** 248–254 (2009).

10. Wang, M., Zhou, Z., O'Connor, J. K. & Zelenkov, N. V. A new diverse enantiornithine family (Bohaiornithidae fam. nov.) from the Lower Cretaceous of China with information from two new species. *Vertebr. Palasiat.* **52,** 31–76 (2014).

11. Zhou, Z. & Zhang, F. A long-tailed, seed-eating bird from the Early Cretaceous of China. *Nature* **418,** 405–409 (2002).

12. Zhang, F. & Zhou, Z. A primitive enantiornithine bird and the origin of feathers. *Science* **290,** 1955–1959 (2000).

13. Lefèvre, U., Hu, D., Escuillié, F., Dyke, G. & Godefroit, P. A new long-tailed basal bird from the Lower Cretaceous of north-eastern China. *Biol. J. Linn. Soc.* **113,** 790–804 (2014).

14. Zhou, Z. & Zhang, F. *Jeholornis* compared to *Archaeopteryx*, with a new understanding of the earliest avian evolution. *Naturwissenschaften* **90,** 220–225 (2003).

15. Zhang, F., Zhou, Z., Xu, X. & Wang, X. A juvenile coelurosaurian theropod from China indicates arboreal habits. *Naturwissenschaften* **89,** 394–398 (2002).

16. McGowan, C. Tarsal development in birds: evidence for homology with the theropod condition. *J. Zool.* **206,** 53–67 (1985).

17. Elzanowski, A. in *Mesozoic Birds: Above the Heads of Dinosaurs* (eds Chiappe, L. M. & Witmer, L. M.) 129–159 (University California Press, 2002).

18. Chiappe, L. M. & Walker, C. A. in *Mesozoic Birds: Above the Heads of Dinosaurs* (eds Chiappe, L. M. & Witmer, L. M.) 240–267 (University California Press, 2002).

19. O'Connor, J. K., Sun, C., Xu, X., Wang, X. & Zhou, Z. A new species of *Jeholornis* with complete caudal integument. *Hist. Biol.* **24,** 29–41 (2011).

20. Wang, M., *et al.* The oldest record of Ornithuromorpha from the Early Cretaceous of China. *Nat. Commun.* **6,** 6987 (2015).
